# A validated, modifiable proteomic score from the EXSCEL trial predicts cardiovascular events in diabetes

**DOI:** 10.1172/jci.insight.204463

**Published:** 2026-07-02

**Authors:** Kristin M. Corey, Maggie Nguyen, Michael Y. Mi, Megan E. Ramaker, Ilya Zhbannikov, Harald Sourij, G. Michael Felker, Naveed Sattar, Jennifer B. Green, Pamela S. Douglas, Robert E. Gerszten, Robert J. Mentz, Adrian F. Hernandez, Rury R. Holman, Bruce M. Psaty, James S. Floyd, Svati H. Shah

**Affiliations:** 1 Duke Molecular Physiology Institute, Duke University School of Medicine, Durham, North Carolina, USA.; 2 Cardiovascular Medicine, Beth Israel Deaconess Medical Center, Boston Massachusetts, USA.; 3 Cardiometabolic Trials Unit, Division of Endocrinology and Diabetology, Medical University of Graz, Graz, Austria.; 4 Duke Clinical Research Institute, Durham, North Carolina, USA.; 5 Institute of Cardiovascular & Medical Sciences, University of Glasgow, Glasgow, United Kingdom.; 6 Division of Cardiology, Department of Medicine, Duke University School of Medicine, Durham, North Carolina, USA.; 7 Diabetes Trials Unit, Radcliffe Department of Medicine, University of Oxford, Oxford, United Kingdom.; 8 Cardiovascular Health Research Unit, Department of Medicine, and; 9 Departments of Epidemiology and Health Systems and Population Health, University of Washington, Seattle, Washington, USA.

**Keywords:** Cardiology, Endocrinology, Atherosclerosis, Biomarkers, Molecular epidemiology

## Abstract

**BACKGROUND:**

Adults with type 2 diabetes mellitus (T2DM) are at increased risk for stroke, myocardial infarction, and cardiovascular death, yet individual risk is heterogeneous and incompletely captured by clinical models.

**METHODS:**

In the Exenatide Study of Cardiovascular Event Lowering (EXSCEL), adults with T2DM were randomized to a GLP-1 RA (exenatide) or a placebo and followed longitudinally for major adverse cardiovascular events (MACE). High-throughput discovery proteomics was done in plasma collected at baseline and 12 months. Proteins associated with time to MACE were identified using multivariable regression and incorporated into supervised machine learning models. A multi-protein score was developed and externally validated in 2 independent population-based and trial cohorts.

**RESULTS:**

The proteomic score showed incremental improvement in cardiovascular risk discrimination beyond clinical factors alone, and several proteins were consistently prioritized across modeling approaches. The protein score and a top-ranked protein, tetranectin, were modified by GLP-1 RA treatment, and a decrease in protein score was associated with improved outcomes, supporting modifiability of MACE risk.

**CONCLUSION:**

External validation confirmed generalizability across cohorts with and without diabetes. Together, these findings demonstrate that plasma proteomic signatures can enhance cardiovascular risk stratification and identify treatment-responsive biomarkers in T2DM, supporting their potential role in precision prevention strategies

**FUNDING:**

The EXSCEL study was funded by Amylin Pharmaceuticals. This research was supported by contracts HHSN268201200036C, HHSN268200800007C, HHSN268201800001C, N01HC55222, N01HC85079, N01HC85080, N01HC85081, N01HC85082, N01HC85083, N01HC85086, 75N92021D00006, and grants R01HL146145, U01HL080295, U01HL130114, R01HL172803, and R01HL144483 from the National Heart, Lung, and Blood Institute, with additional contribution from the National Institute of Neurological Disorders and Stroke. Additional support was provided by R01AG023629 from the National Institute on Aging.

## Abstract 2

Blood proteins help predict heart disease risk in diabetes, and GLP-1 therapy modifies these proteins, improving risk assessment beyond traditional clinical factors.

## Graphical abstract

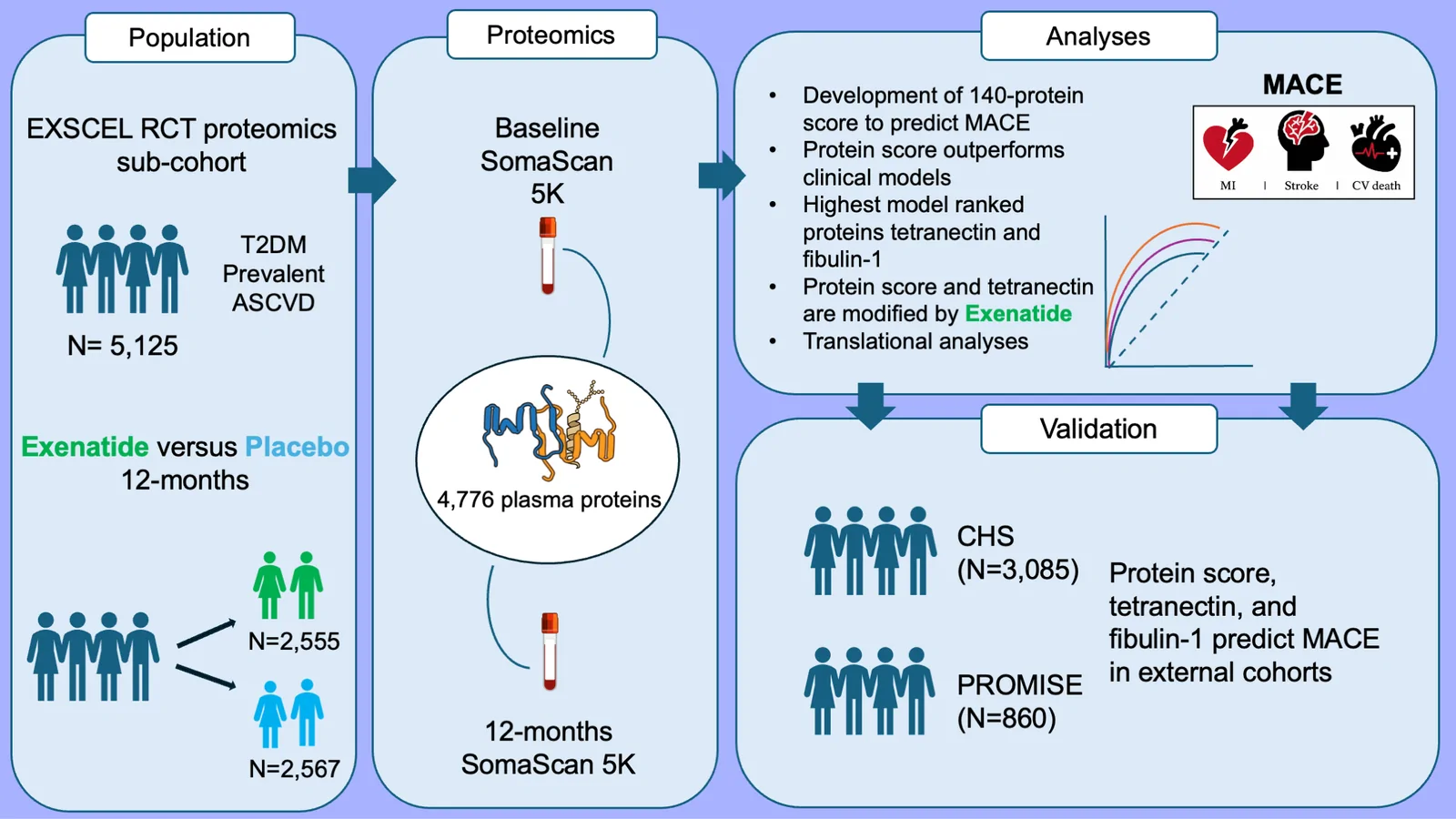

## Introduction

Cardiovascular disease remains the leading cause of mortality in the world, accounting for nearly 1 in every 5 deaths annually. Despite advances in prevention and treatment, the burden of major adverse cardiovascular events (MACE), including nonfatal stroke, nonfatal myocardial infarction, and cardiovascular death, remains elevated, largely driven by an aging population and increasing prevalence of risk factors such as obesity and type 2 diabetes mellitus (T2DM) (1). Unfortunately, despite widely accepted high-risk stratification tools such as the Atherosclerotic Cardiovascular Disease (ASCVD) risk calculator, Framingham risk score, Systematic Coronary Risk Evaluation 2 (SCORE2), and ADVANCE score (with specific T2DM clinical features), the accuracy of these clinical prediction algorithms is incomplete in identifying individuals who will develop MACE (2, 3). These clinically utilized prediction scores consist of traditional comorbidity-based risk factors including age, T2DM, hypertension, and smoking status, among others. However, there remains marked heterogeneity for risk of MACE, making it difficult to accurately stratify this population based on clinical comorbidities alone.

Furthermore, the growing recognition of the significant health benefits of glucagon-like peptide-1 receptor agonists (GLP-1 RA) to provide additional cardiovascular protection for people with T2DM as well as obesity have made high-risk MACE stratification even more urgent (4, 5). Multiple large meta-analyses and randomized controlled trials of GLP-RAs have demonstrated up to 14% reduction of MACE in patients both with and without T2DM (6–8). Although it is well-established that GLP-1 RA treatment prevents MACE in individuals with T2DM and obesity, the potential pleiotropic biological mechanisms of the treatment effect are incompletely understood. Clinically, these incretin mimetic therapies can result in significant weight loss and improve glycemic control by slowing gastric emptying and optimizing insulin and glucagon secretion (9, 10). However, clinical trials have demonstrated a reduction in MACE with GLP-1 RA therapy despite minimal weight loss, raising the possibility of direct tissue effects on atherosclerosis (11, 12). Further studies have demonstrated improvement in systemic inflammation, endothelial function, and attenuation of platelet aggregation (13–15). Given the high cost of and significant demand for GLP-1 RA therapy, identifying particular patients who may benefit most from these medications is a priority (9, 16).

**Figure 1:**
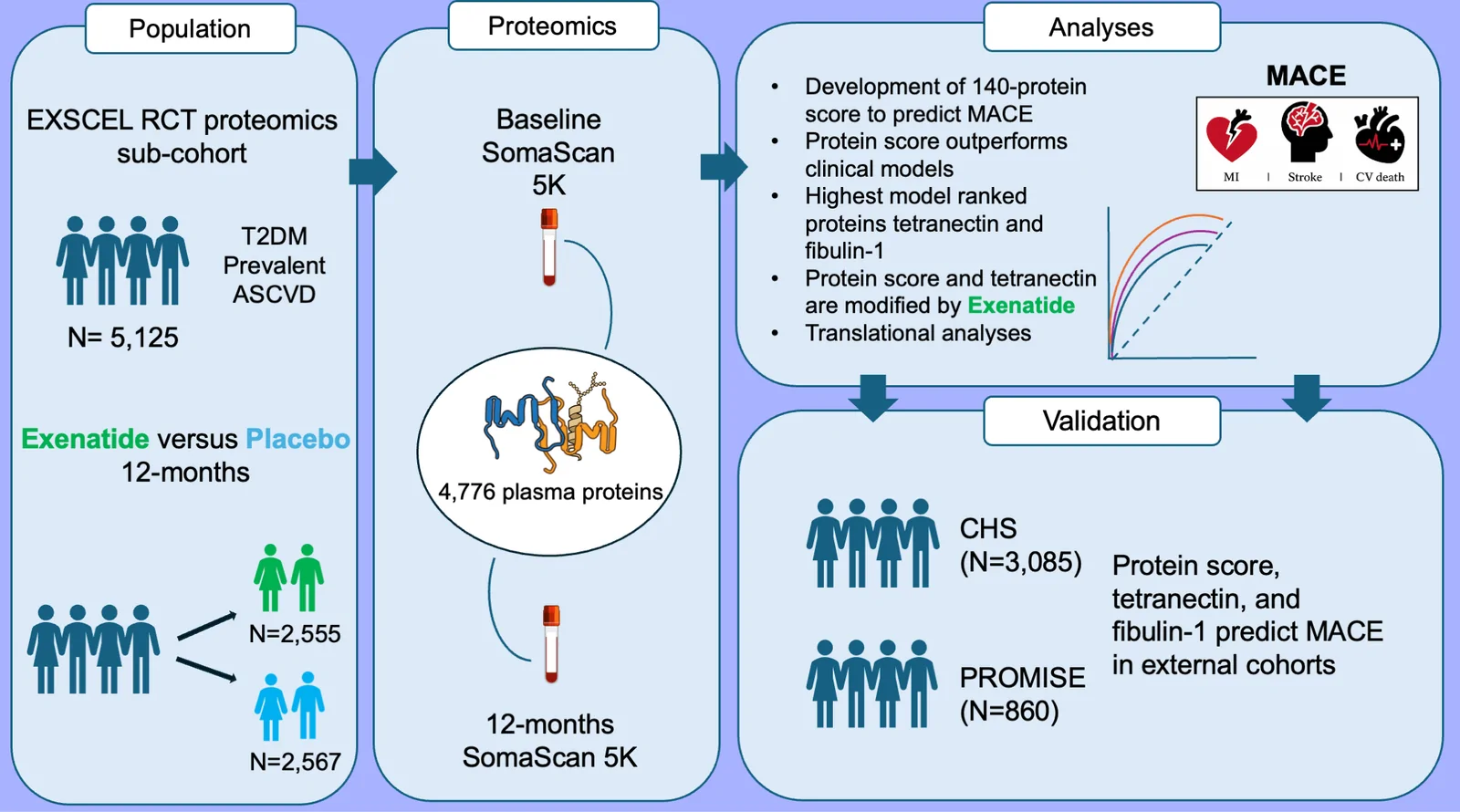
Study overview and analytic workflow. Overview of the EXSCEL proteomics subcohort, proteomic profiling, analytic framework, and external validation strategy. A randomized EXSCEL subcohort with available plasma samples underwent SomaScan 5K proteomic profiling at baseline and 12 months. Baseline proteins were evaluated for association with MACE, and prioritized proteins were incorporated into supervised machine learning models to develop a 140-protein risk score. Additional analyses included treatment responsiveness, biological contextualization analyses, and external validation in the CHS and PROMISE cohorts. MACE included nonfatal myocardial infarction, nonfatal stroke, and cardiovascular death. ASCVD, atherosclerotic cardiovascular disease; CHS, Cardiovascular Health Study; EXSCEL, Exenatide Study of Cardiovascular Event Lowering; MACE, major adverse cardiovascular events; PROMISE, Prospective Multicenter Imaging Study for Evaluation of Chest Pain; RCT, randomized controlled trial; T2DM, type 2 diabetes mellitus.

Recent developments in high-throughput discovery proteomics offer a promising approach to predicting poor health outcomes (17, 18). This technology has the ability to quantify thousands of molecules from a single plasma sample and the potential to not only identify otherwise unknown biomarkers but shed light on biological underpinnings that give rise to the heterogeneity of health risks for deleterious outcomes such as MACE (19, 20). Thus, the primary objective of this study is to use high-throughput discovery proteomics with machine learning, external validation, and assessment of therapeutic modulation (Figure 1) to identify circulating biomarkers with potential clinical utility for MACE risk prediction, including evaluation of performance above clinical factors and potential therapeutic modifiability by a commonly used medication class (GLP-1 RA) with beneficial effects on MACE. These secondary analyses were intended to interpret the prioritized proteins rather than to comprehensively map the causal or pathway architecture of MACE, which remained beyond the scope of this predictive study.

## Results

### Baseline participant characteristics.

The Exenatide Study of Cardiovascular Event Lowering (EXSCEL) discovery substudy cohort had a high prevalence of previous cardiovascular disease at baseline (72.2%); 14.7% (*n* = 758) of participants experienced MACE during the study period (placebo 380/2,560 [14.8%] and GLP-1 RA 378/2,565 [14.7%]) over a median follow-up time of 3.2 years (IQR 2.2, 4.4). Participants who experienced MACE tended to be older, male, and with a higher prevalence of traditional risk factors, including history of ASCVD, duration of T2DM, and smoking, but were similar in BMI, LDL cholesterol (LDL-C), HbA1c, and creatinine concentration to those who did not experience MACE (Table 1). The EXSCEL proteomics cohort and the parent EXSCEL trial cohort had similar demographic characteristics, although the proteomics cohort had a lower prevalence of individuals from self-reported non-White races (Supplemental Table 1; supplemental material available online with this article; https://doi.org/10.1172/jci.insight.204463DS1). Supplemental Tables 2 and 3 list baseline participant characteristics for the validation cohorts, the Cardiovascular Health Study (CHS) and Prospective Multicenter Imaging Study for Evaluation of Chest Pain (PROMISE), respectively. Having established the baseline clinical characteristics of the EXSCEL proteomics cohort, we next evaluated whether circulating proteins could improve prediction of MACE beyond traditional clinical factors.

### Protein model and protein score development.

**Figure 2:**
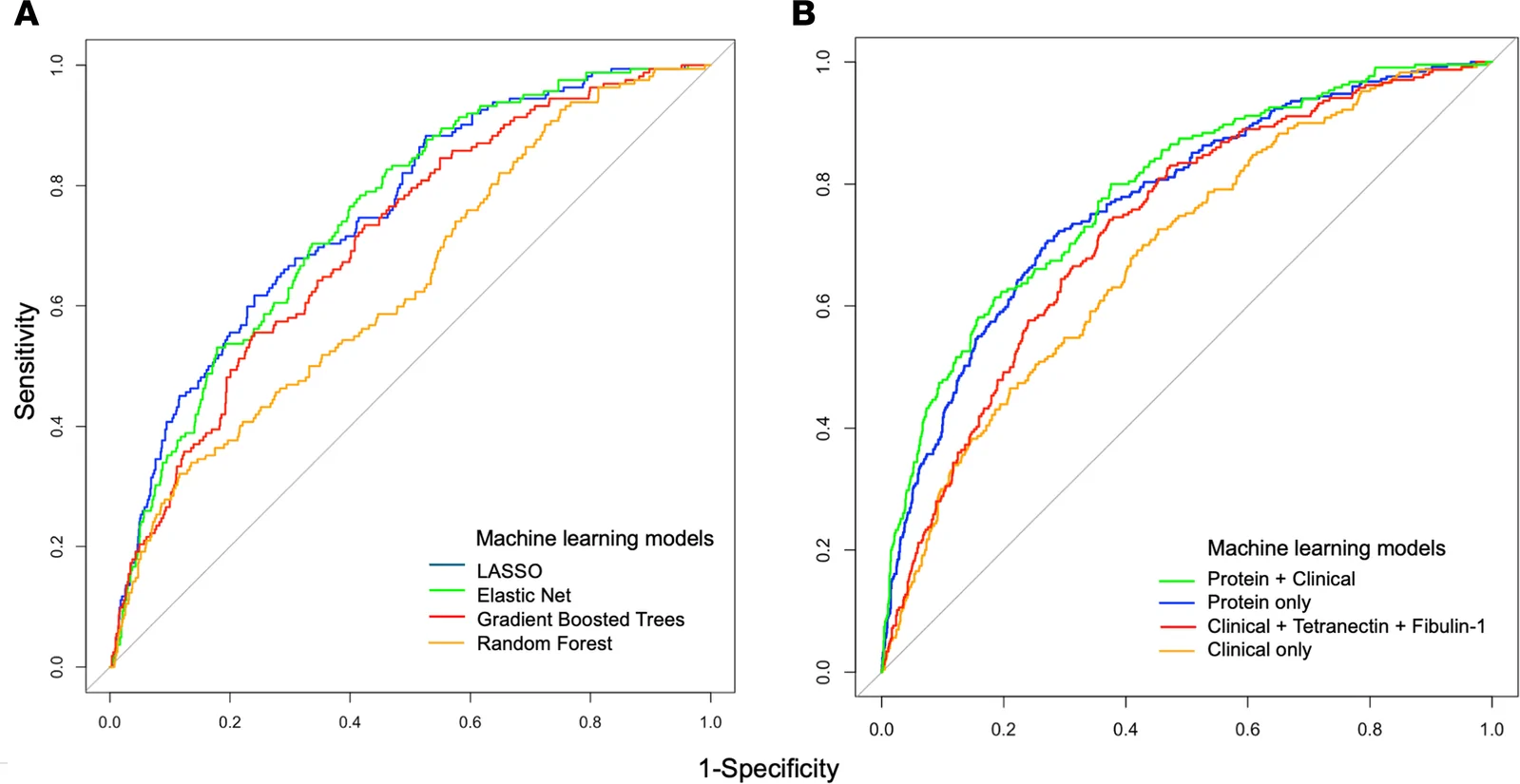
Receiver operating characteristic curves for proteomic and clinical prediction models in the EXSCEL proteomics cohort. (**A**) Receiver operating characteristic (ROC) curves for proteomic machine learning models (LASSO, elastic net, random forest, and gradient-boosted decision trees) for discrimination of MACE. (**B**) ROC curves for protein-only, clinical-only, and protein plus clinical models. Clinical features were derived from the SCORE2, ADVANCE, and ASCVD Risk Estimator models. All ROC curves were evaluated in the held-out test set of the EXSCEL proteomics cohort.

Baseline proteins associated with MACE (FDR *P* < 0.05) in Cox proportional hazard models were used as input variables in prediction models (Supplemental Table 4). Penalized regression models showed higher AUROCs than decision tree–based models (random forest and gradient-boosted decision trees) in discriminating MACE events and overall demonstrated high predictive performance for MACE (AUROC 0.75 [95% CI: 0.72–0.78] vs. 0.64 [95% CI: 0.60–0.66], respectively, *P* < 0.001) (Figure 2, Table 2, and Supplemental Table 6), prioritizing 140 proteins in the held-out test set (Supplement Table 5).

In assessing discriminative capability and improvement over a clinical model, the LASSO model demonstrated higher discrimination of MACE than the clinical model (AUROC 0.75 [95% CI: 0.72–0.78] vs. 0.69 [95% CI: 0.66–0.73], respectively, *P* < 0.05), and added to a clinical model (clinical plus protein: AUROC of 0.79 [95% CI: 0.76–0.82], *P* < 0.05). The area under the precision-recall curve (AUPRC) of the protein plus clinical model was higher than the protein-only model (AUPRC 0.40 [95% CI: 0.37–0.44] vs. AUPRC 0.46 [95% CI: 0.40–0.52], respectively, *P* < 0.001). Both of these models had higher AUPRC compared with the clinical model (AUPRC 0.25 [95% CI: 0.22–0.29], *P* < 0.001) for difference in AUPRC of clinical-only versus clinical plus protein models and clinical-only versus protein-only models (Figure 2, Table 3, and Supplemental Table 7). Consistent with the reclassification patterns shown in Table 5, the protein score demonstrated an overall categorical net reclassification index of 0.21 and an integrated discrimination improvement index of 0.023 relative to the clinical features–only model, indicating clinically significant improved risk reclassification beyond clinical features alone.

A protein score was then developed from the LASSO protein model weights. The protein score was predictive of MACE in the held-out test set (EXSCEL protein score HR per 1 SD increase (standardized score) 2.24 [95% CI: 2.16–2.32], *P* ≤ 2.25 × 10^–7^ and AUROC 0.70 [95% CI: 0.69–0.71]). A Brier score of 0.091, Hosmer-Lemeshow *P* value of 0.93, and risk stratification table (Table 4) demonstrated appropriate model calibration. Across prespecified risk strata in the held-out test set, observed MACE rates increased from 0.4% (<5% risk) to 10.0% (≥20% risk) for the protein score, demonstrating clear risk separation. In cross-classification with the clinical model, 42% of individuals categorized as 20% or higher risk by the clinical model were reclassified into lower protein-score risk categories, while the highest protein-score stratum retained the highest observed event rate, consistent with improved specificity and risk stratification (Table 5).

**Figure 3:**
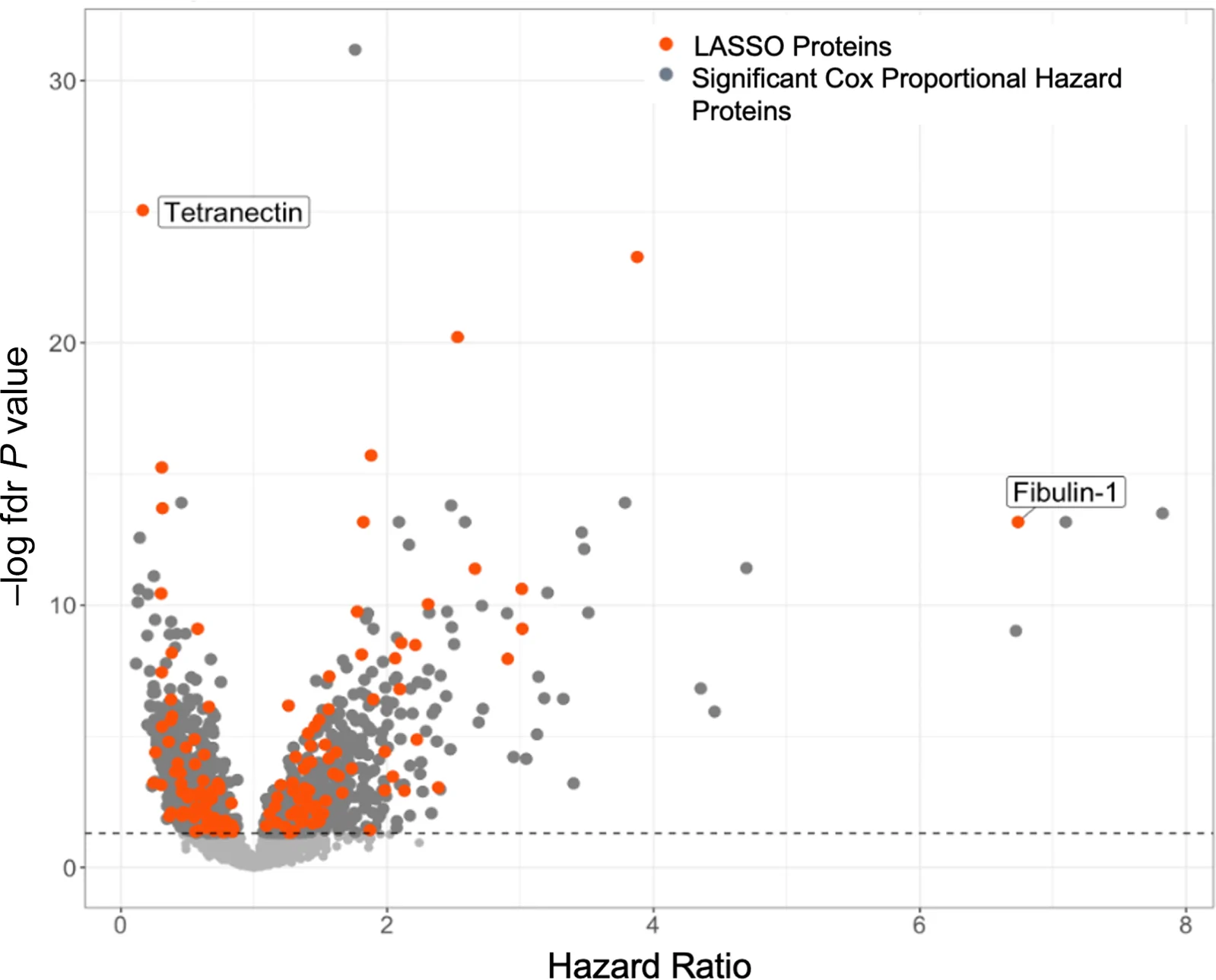
Volcano plot demonstrating model-prioritized MACE proteins in EXSCEL proteomics cohort. Multivariate Cox proportional hazards models identified 1,278 individual proteins that were significantly associated with MACE (FDR adjusted *P* < 0.05) in the EXSCEL proteomics cohort (gray); 140 of these proteins were prioritized in the LASSO model (red). The 2 proteins with the absolute highest LASSO model weights were tetranectin and fibulin-1.

To better understand which individual proteins were driving performance of the proteomic models, we next examined protein prioritization across the Cox and machine learning approaches. The individual protein Cox proportional hazard models and proteomic machine learning models, including tree-based and penalized regression models, all prioritized tetranectin (tetranectin: Cox model HR 0.11 [95% CI: 0.07–0.16], FDR adjusted *P* = 2.33 × 10^−10^) and fibulin-1 (Cox model HR 6.48 [95% CI: 4.13–10.19], FDR adjusted *P* = 3.14 × 10^−6^), demonstrating concordance in protein prioritization across multiple statistical methods and robustness in the protein association with MACE (Figure 3). Not only were these 2 proteins consistently prioritized across multiple models with the highest Gini index–based importance scores, they also were assigned the highest absolute LASSO model weights amongst prioritized proteins (tetranectin, –0.11; fibulin-1, 0.10) (Supplemental Figure 1 and Supplemental Table 5). In bootstrap stability analyses (1,000 replicates), the 2 most consistently prioritized proteins were very frequently reselected (tetranectin, 91.3% and fibulin-1, 89.6%), supporting the robustness of these proteins.

Given the strong importance of these specific proteins in predicting MACE, we tested the prediction strength of these proteins alone. Tetranectin and tetranectin plus fibulin-1 showed decent MACE discrimination (AUROC 0.61 [95% CI: 0.59–0.64] and 0.65 [95% CI: 0.62–0.68], respectively; and AUPRC 0.31 [95% CI: 0.28–0.33] and 0.35 [95% CI: 0.32–0.38], respectively). Further, the addition of these 2 proteins to the clinical model improved discrimination, yielding an AUROC of 0.74 (95% CI: 0.71–0.78) and an AUPRC of 0.30 (95% CI: 0.26–0.35), with an increase in AUROC of 0.05 and AUPRC of 0.05 compared with the clinical features–only model (*P* = 0.017). Notably, the AUROC of the clinical plus tetranectin and fibulin-1 model was similar to the full protein-only model, though the AUPRC was higher in the full protein-only model (AUROC of 0.74 [95% CI: 0.71–0.78] and AUPRC 0.30 [95% CI: 0.26–0.35]) versus AUROC of 0.75 [95% CI: 0.72–0.78] and AUPRC 0.40 [95% CI: 0.37–0.44], *P* = 0.017, respectively), demonstrating the predictive strength of tetranectin and fibulin-1 for MACE. Both models had a higher point-estimate discrimination than the clinical features–only model (ΔAUPRC 0.044 [95% CI: 0.007–0.082], *P* = 0.017) (Figure 2, Table 3, and Supplemental Table 7). Because tetranectin and fibulin-1 were the most consistently prioritized proteins, we then evaluated their relationships with established clinical features associated with cardiovascular risk to better contextualize these biomarkers.

### Tetranectin and fibulin-1 were associated with history of ASCVD, baseline BMI, and HbA1c.

Tetranectin and fibulin-1 were associated with history of ASCVD in the EXSCEL cohort in multivariable logistic regression analyses (tetranectin: OR per 1 SD 0.30 [95% CI: 0.19–0.49], *P* = 0.003 and fibulin-1: OR per 1 SD 3.86 [95% CI: 2.40–6.24], *P* = 9.32 × 10^–4^). To better understand this relationship between tetranectin, fibulin-1, history of ASCVD, and MACE, we tested their correlation with baseline ASCVD prevalence in EXSCEL. Baseline tetranectin levels were lower (*P* < 0.05) and baseline circulating levels of fibulin-1 were higher in individuals with a history of ASCVD compared with individuals without a history of ASCVD (*P* < 0.05). Furthermore, subset analyses on cohorts with a history of ASCVD demonstrated that patients who developed MACE had significantly lower levels of baseline tetranectin (*P* = 0.038) and higher levels of baseline fibulin-1 (*P* = 0.029) compared with patients without MACE, providing further stratification for risk of MACE (Supplemental Figure 2 and Supplemental Table 8, Supplemental Figure 3, and Supplemental Table 9). Baseline tetranectin and fibulin-1 levels were correlated with other known high-risk clinical features associated with MACE, such as baseline BMI (*r* = –0.06, *P* = 0.023 and *r* = 0.14, *P* = 1.80 × 10^−7^, respectively) and baseline HbA1c (tetranectin *r* = –0.13, *P* = 2.48 × 10^−7^). We next tested whether the prioritized proteomic signature and top-ranked proteins were biologically responsive to GLP-1 RA treatment over time.

### GLP-1 RA treatment beneficially modified protein score and tetranectin.

**Figure 4:**
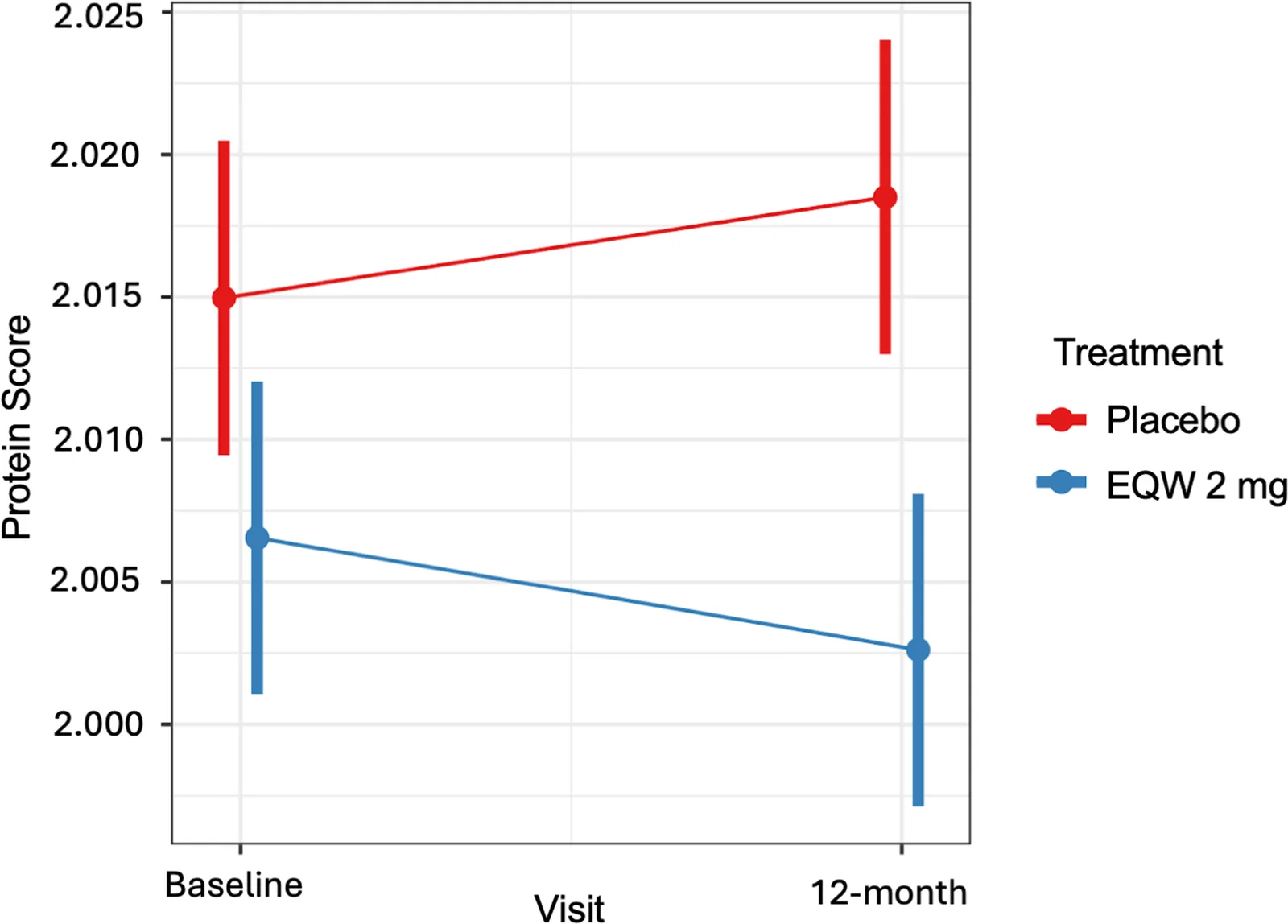
Differential change in MACE proteomics score with GLP-1 RA treatment in EXSCEL. Linear mixed model adjusted for change in BMI and HbA1c demonstrated differential change in the MACE protein score with GLP-1 RA treatment over 12 months (*P* = 0.005).

Linear mixed-effect modeling adjusted for change in BMI and HbA1c was used to assess whether there was a differential change in high-risk proteins in participants randomized to GLP-1 RA treatment versus a placebo. Model results demonstrated that the protein score decreased and tetranectin levels increased in the treatment arm compared with the placebo arm (*P* = 0.005 and *P* = 0.002, respectively) (Figure 4). Visual inspection of raw protein distributions showed only modest shifts over time, consistent with the small mean changes observed in each treatment arm (Supplemental Figure 4 and Supplemental Table 10). Fibulin-1, however, did not show significant modification by GLP-1 RA treatment after 1 year.

Given these results and the known strong weight loss effects of GLP-1 RA, we next evaluated whether treatment-associated protein changes were mediated by weight loss from baseline to 12 months. Of the 140 proteins in the proteomic score, 47 were significantly modified by GLP-1 RA (*P* < 0.05) (Supplemental Table 5). Among these 47 treatment-responsive proteins, 24 showed statistically significant mediation by weight loss in the same direction as the direct effect. However, the magnitude of mediation was generally modest, with 15 of these 24 proteins showing less than 15% of the treatment effect explained by weight loss. Together, these results suggest that most GLP-1 RA–associated protein changes primarily reflect weight-loss–independent mechanisms (Supplemental Table 11).

### Longitudinal change in protein score for incident MACE.

Having established that higher levels of the baseline protein score were predictive of incident MACE, we next tested whether change in the protein score from baseline to 12 months was also prognostic of subsequent MACE. In a 12-month landmark analysis (*n* = 3,742; 409 events), a decrease in the scaled protein score from baseline to 12 months, modeling a directionally beneficial decrease, was associated with lower subsequent MACE risk (HR per 1 SD decrease 0.68 [95% CI 0.59–0.79], *P* < 0.05). These findings indicate that a decrease (i.e., improvement) in the protein score over time is prognostic of improved incident MACE outcomes.

### Protein score and individual top-ranked proteins validated in CHS and PROMISE cohorts.

**Figure 5:**
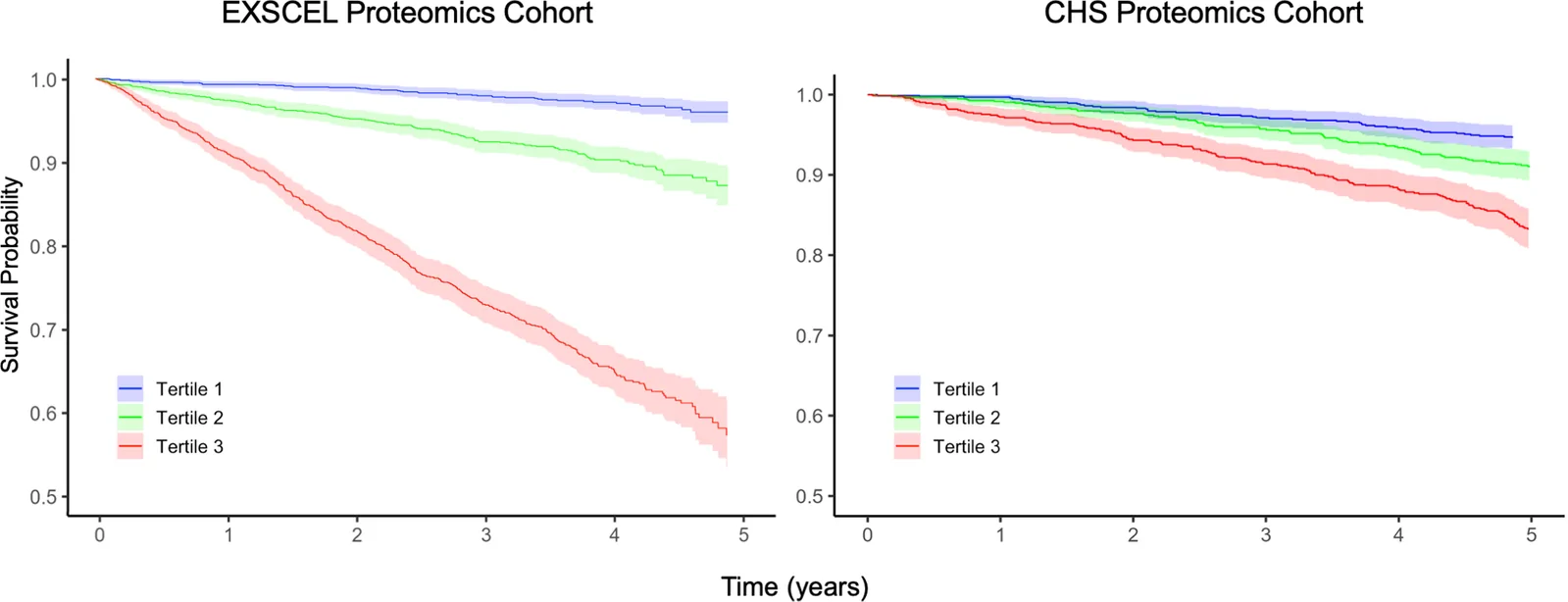
Kaplan-Meier survival curves comparing tertiles of the MACE protein score in EXSCEL and CHS cohorts. Survival curves demonstrate multivariate Cox proportional hazard models for time to MACE and tertiles of protein score representing high, medium, and low risk of MACE across the 2 cohorts.

The model weights from the protein score were applied to matching proteins in the CHS cohort (T2DM and non-T2DM). The protein score’s ability to predict time to MACE was validated in the CHS cohort with multivariable Cox proportional hazard models using held-out test sets (CHS protein score HR per 1 SD increase [standardized score] 1.33 [95% CI: 1.17–1.52], *P* = 1.6 × 10^–5^ and AUROC 0.68 [95% CI: 0.65–0.72]). Association of protein score tertiles with time-to-MACE risk was performed for visualization (Kaplan-Meier curves) in both EXSCEL and CHS, demonstrating a linear increase in the risk of MACE across increasing tertiles of the protein score (Figure 5). Although tetranectin was associated with time to MACE in the overall CHS cohort (tetranectin HR 0.82 per 1 SD [95% CI: 0.73–0.93], *P* = 0.002), fibulin-1 was not.

The full protein score could not be assessed in the PROMISE cohort due to differences in proteomics platforms. However, in multivariable logistic regression models, tetranectin was associated with MACE (MACE OR per 1 SD 0.88 [95% CI: 0.80–0.98] *P* = 0.013) in the overall PROMISE cohort, but fibulin-1 was not.

### Protein score and tetranectin were associated with baseline ASCVD in CHS and PROMISE.

To further characterize the clinical relevance of the validated biomarkers, and given the known strong association of prevalent ASCVD with incident MACE, we then examined their associations with prevalent ASCVD and coronary disease burden in the external cohorts. The protein score was significantly associated with baseline ASCVD in CHS (OR 1.04 per 1 SD increase in score [95% CI: 1.03–1.06], *P* = 2 × 10^−11^). Furthermore, similar to the results found in the EXSCEL cohort, lower levels of baseline tetranectin were associated with history of ASCVD in multivariable regression analyses in both validation cohorts: (CHS OR 0.98 per 1 SD [95% CI: 0.97–0.99], *P* = 0.003; PROMISE OR per 1 SD 0.80 [95% CI: 0.68–0.93], *P* = 0.004), and lower levels of baseline tetranectin were associated with history of coronary artery disease (CAD) in multivariable regression analyses in both replication cohorts (CHS tetranectin OR per 1 SD 0.99 [95% CI: 0.98–0.99], *P* = 0.04; PROMISE tetranectin OR per 1 SD 0.80 [95% CI: 0.68–0.93], *P* = 0.004). Fibulin-1 protein levels were not associated with ASCVD or CAD in either cohort (Supplemental Table 12). Furthermore, Pearson’s correlation analyses demonstrated significant negative correlations between tetranectin levels and CT angiography–adapted Leaman scores in the PROMISE cohort (*r* = –0.20, *P* = 4.36 × 10^−4^). Taken together, these findings indicate that lower circulating tetranectin is consistently associated with prevalent atherosclerotic disease across independent cohorts, supporting its role as a marker of underlying cardiometabolic risk rather than a cohort-specific signal.

### Protein score and top-ranked proteins’ association with MACE were not specific to T2DM.

Because our protein score was developed in a T2DM cohort, we sought to evaluate whether its strong predictive abilities were unique to MACE in people with T2DM or MACE in general. Thus, we performed analyses in T2DM-only and non-T2DM subsets in the CHS cohort, which demonstrated that the protein score remained associated with MACE in multivariable Cox proportional hazard models regardless of T2DM status (T2DM CHS *n* = 381: HR 1.32 per 1 SD [95% CI: 1.02–1.71], *P* = 0.04; non-T2DM CHS *n* = 2,382: HR 1.33 per 1 SD [95% CI: 1.15–1.55], *P* = 2.1 × 10^–4^). Tetranectin and fibulin-1 were also evaluated in T2DM and non-T2DM subsets in the validation cohorts. Tetranectin was associated with time to MACE in multivariable analyses in the subset of participants without T2DM (CHS non-T2DM *n* = 2,382: HR 0.82 per 1 SD [95% CI: 0.71–0.94], *P* = 0.005; PROMISE non-T2DM *n* = 681: HR 0.85 per 1 SD [95% CI: 0.76–0.94], *P* = 0.002), but fibulin-1 was not (Supplemental Table 12). In the CHS T2DM subset, tetranectin showed a similar magnitude of effect for time to MACE, but was not statistically significant possibly due to sample size (CHS T2DM *n* = 381, tetranectin: HR 0.85 per 1 SD [95% CI: 0.65–1.10], *P* = 0.21). Interestingly, baseline tetranectin in the PROMISE cohort was also significantly lower in patients with T2DM compared with those without T2DM (OR per 1 SD 0.08 [95% CI: 0.05–0.11], *P* = 5 × 10^−5^) (Supplemental Figure 5 and Supplemental Table 13). Overall, these results demonstrate that the protein score and tetranectin levels are not unique to T2DM as they predict MACE in individuals with and without T2DM. To explore whether the top-ranked proteins might reflect causal mediators of risk rather than biomarkers of underlying disease burden, we next performed Mendelian randomization analyses.

### Mendelian randomization analyses suggest that tetranectin and fibulin-1 are not in the causal pathway for MACE or CAD.

We focused these analyses on tetranectin and fibulin-1 because they were the most consistently prioritized and most stable proteins in our study, reselected in 91.3% and 89.6% of bootstrap replicates, respectively, and ranked highest across the Cox, tree-based, and penalized regression models. Given their dominant contribution to model performance, we sought to determine whether they were likely to act as causal mediators of risk or instead as biomarkers of underlying disease burden. Inverse variance–weighted Mendelian randomization analyses did not demonstrate an association between tetranectin with MACE and CAD (nonfatal stroke: β estimate = –0.032, SE = 0.022, *P* = 0.12, nonfatal myocardial infarction: β = 0.01, SE = 0.19, *P* = 0.5, cardiovascular death: β = 0.087, SE = 0.067, *P* = 0.21, CAD: β = −0.082, SE = 0.15 *P* = 0.6). Similarly, fibulin-1 was not associated with MACE or CAD (nonfatal stroke: β = −0.14, SE = 0.31, *P* = 0.9; nonfatal myocardial infarction: β = −0.074, SE = 0.33, *P* = 0.8; cardiovascular death: β = 0.031, SE = 0.02, *P* = 0.2; CAD: β = 0.044, SE = 0.049, *P* = 0.4). These results suggest that neither tetranectin nor fibulin-1 are likely in the causal pathway for CAD or MACE. The Mendelian randomization analysis null findings therefore reflect a deliberate causal test applied to our most reproducible biomarkers, and supports their interpretation as markers of residual atherosclerotic and fibrotic risk rather than as causal targets.

### Gene set enrichment analysis identified enriched protein complement pathway.

Finally, to place the prioritized proteins in broader biological context, we performed pathway enrichment analyses using proteins associated with MACE in the discovery cohort. Because enrichment was evaluated at the aggregate gene-set level across the full ranked list of nominally significant proteins, rather than relying on the stable selection of any single protein, these results are relatively robust to the prioritization instability of individual proteins and are presented as hypothesis-generating biological context for the MACE-associated proteome. Pathway analyses were conducted on proteins that were nominally significant from the initial discovery multivariable Cox proportional hazard models (1,278 proteins; Supplement Table 1), highlighting enrichment of the complement pathway (FDR < 0.05) and top-ranked KEGG pathways of fatty acid metabolism (nominal *P* = 0.01) and cardiac muscle contraction (nominal *P* = 0.04).

## Discussion

Current methods for predicting MACE in high-risk patients are largely limited to clinical feature–based models (21). Leveraging multiple machine learning approaches in a unique, large randomized controlled trial of GLP-1 RA therapy in patients with T2DM as well as disease-based and population-based validation cohorts, we developed a protein score by identifying individual proteins associated with MACE at FDR less than 0.05 and highlighted the power of 2 specific proteins, tetranectin and fibulin-1, in predicting MACE. Supporting the generalizability of the findings, we found these results were consistent in patients with and without T2DM. The protein score and a high-risk individual protein, tetranectin, were beneficially modified by GLP-1 RA treatment (exenatide), and a favorable decrease in the proteomic score was associated with lower subsequent MACE risk. Importantly, the protein model added discriminative capability for MACE and resulted in clinically significant net reclassification over a clinical model, supporting the clinical utility of the results. To our knowledge, our current study is the largest proteomics-based analysis developing and externally validating a predictive MACE risk score in patients with T2DM.These results in combination support the potential clinical utility of the identified protein score not only for MACE prediction but for monitoring change in risk, which can be targeted by a now commonly used therapy for preventing MACE.

Although our primary goal was identification of clinically relevant protein biomarkers, at a biological level, pathway analysis of these proteins demonstrated upregulation of complement, fatty acid metabolism, and cardiac contractility pathways consistent with underlying inflammatory, cardiac, and metabolic dysfunction in patients at high risk for MACE. Although neither of the prioritized proteins are likely in the causal pathway for MACE based on our Mendelian randomization results, they may reflect high-risk residual atherosclerosis that is difficult to capture clinically.

Clinical feature–based models used to predict MACE, such as SCORE2, ADVANCE, and ASCVD Risk Estimator Plus, utilize the presence of many high-risk comorbidities (2, 3). When evaluating some of these high-risk features within the EXSCEL cohort, however, there were no statistical differences in baseline BMI, LDL-C, total cholesterol, systolic blood pressure, HbA1c, and creatinine concentrations between those who developed MACE and those who did not, highlighting the limitations of utilizing only clinical data to discriminate high-risk populations. Using these same clinical features, we demonstrated that our protein score predicted MACE with higher accuracy and with improved clinical discrimination compared with clinical features alone in the EXSCEL cohort, which had a high prevalence of prior ASCVD. This protein score also demonstrated accuracy in predicting MACE in a population-based cohort (CHS) with and without T2DM, further supporting the generalizability of the score across patient cohorts.

Previous studies have used a similar approach of combining clinical features and high-throughput proteomics analyses applied to T2DM cohorts to better prioritize molecular signatures that more accurately predict MACE within this population (17, 18, 22–27). There was no overlap between proteins prioritized by our study and these other studies, likely due to smaller sample sizes and differing targeted proteomics methods utilized by the previous studies, limiting their power and validation (22–26). The proteins prioritized by our model did, however, overlap with other large discovery proteomics studies predicting ASCVD risk in generalized cohorts. For example, in a study by Ganz et al. (28) that led to the development of the SomaSignal CVD test, a 9-protein model was identified that outperformed the Framingham Risk Score. Four of the 9 proteins in their model (MMP-12, angiopoietin-2, thrombospondin-2, and cathepsin H) were also prioritized by our machine learning models for MACE prediction, providing supportive external consistency for our study (i.e., external validation) and highlighting how biomarkers can focus on important subtypes of individuals at risk of MACE. Similarly, Helgason et al. (29) developed a 70-protein LASSO model to predict MACE in a generalized cohort. Five of those proteins (MMP-12, receptor-type tyrosine-protein phosphatase delta, cartilage intermediate layer protein 2, coiled-coil domain-containing protein 126, protocadherin gamma-A) were selected by our machine learning models as well. These 2 studies, combined with our own, demonstrate the potential importance of MMP-12, an endopeptidase, in the prediction of MACE across T2DM and non-T2DM populations. Despite conducting large exploratory proteomics analyses in a T2DM cohort (EXSCEL), our protein score was generalizable to a population-based cohort with and without T2DM (CHS). The overlap in important proteins between our study and prior large cohort studies further supports this generalizability.

We identified 2 individual proteins, tetranectin and fibulin-1, as having the largest influence on MACE prediction in our discovery T2DM cohort. This finding was consistent across decision tree and regression-based models and was further validated in 2 large external cohorts: CHS and PROMISE. Tetranectin is expressed in adipose tissue, vasculature, and myocardium, where it is involved in adipogenesis and promotion of proteolytic activation of fibrinolysis by binding to plasminogen (30, 31). Previous studies have also demonstrated that, in the setting of myocardial fibrosis, high intracardiac levels of tetranectin are associated with subsequent low circulating levels of tetranectin (31–34). Similar to our protein score, tetranectin was also beneficially modified by GLP-1 RA treatment and shown to have negative associations with prior ASCVD and CT angiography–adapted Leaman scores in CHS and PROMISE, respectively. Subset analyses in PROMISE demonstrated that individuals with T2DM also had lower levels of circulating tetranectin compared with individuals without T2DM. Furthermore, in participants without T2DM, those who developed MACE had lower levels of tetranectin compared with those who did not, highlighting the generalizability of tetranectin as a biomarker for MACE. Similarly, Ferrannini et al. demonstrated that patients with T2DM at high risk for MACE had lower circulating amounts of plasma tetranectin compared with patients who were not at high risk for MACE (27). This pattern is consistent with other studies reporting lower circulating levels of tetranectin in patients with higher atherosclerotic plaque burden compared with healthy controls (30, 31, 35). To better elucidate the relationship between tetranectin and MACE, we used Mendelian randomization and demonstrated that tetranectin is not in the causal pathway for MACE or CAD. We hypothesize that patients with lower circulating plasma levels of tetranectin may have residual CAD that is not captured by traditional CAD phenotyping, leading to a higher risk of acute thrombotic activity. This residual CAD can trigger a systemic fibrinolytic reaction and, in turn, cause greater systemic tetranectin consumption and intimal accumulation, therefore indicating that those with lower levels of circulating tetranectin are at higher risk of developing MACE. We also hypothesize that tetranectin has high intracardiac accumulation when fibrotic tissue is present due to its ability to bind to extracellular matrix and promote fibrinolytic activity. In this study, we demonstrated that tetranectin is a marker for risk of MACE by potentially reflecting increased atherosclerotic burden and intracardiac fibrosis in generalized populations.

Fibulin-1 is an extracellular matrix glycoprotein that is found in elastin fibers and basement membranes of vascular endothelium. Prior studies have also demonstrated its presence in atherosclerotic plaque (36, 37). Despite the strong association of fibulin-1 with MACE in our discovery cohort, it was not associated with MACE or CAD in the generalized validation cohorts and T2DM subset analyses, though likely due to small sample sizes of the T2DM subsets in CHS and PROMISE. We determined that fibulin-1 was not in the causal pathway for MACE or CAD, nor was it modified by GLP-1 RA treatment. In the EXSCEL cohort, additional analyses demonstrated that baseline fibulin-1 was higher in individuals with a history of ASCVD who developed MACE compared with those without a history of ASCVD. These results align with prior studies that demonstrate upregulation of fibulin-1 in individuals with T2DM, largely reflective of endothelial dysfunction and subsequent arterial stiffness and vascular fibrosis (38, 39). Because of fibulin-1’s strong association with MACE and history of ASCVD in the EXSCEL cohort, we hypothesize that it is a biomarker for endothelial dysfunction, particularly in T2DM cohorts who face a higher prevalence of vascular disease overall. Our results demonstrate that use of tetranectin and fibulin-1 levels as biomarkers may have actionable clinical benefits for patients, and further studies are needed to better elucidate this potential.

Because the discovery cohort for this study was a randomized controlled trial of a GLP-1 RA treatment with longitudinal sampling, we were able to assess and demonstrate that GLP-1 RA treatment beneficially modified our protein score and tetranectin. Particularly in the context of cardiovascular disease risk, beneficial effects of GLP-1 RA treatment are not fully understood. Prior studies have demonstrated, however, that GLP-1 RA treatment reduced atherogenesis and increased plaque stability by suppressing foam cell formation, reducing epicardial fat, and decreasing inflammation (40). We demonstrated that baseline proteins associated with MACE were enriched for complement pathways, and that lower levels of tetranectin were highly predictive of MACE in generalized cohorts. Because our protein score and tetranectin were both demonstrated to be beneficially modified by GLP-1 RA treatment, we hypothesize that these drugs modify inflammatory activation, thereby reducing atherothrombotic progression. Mediation analyses further showed that although some GLP-1 RA–modified proteins were mediated by weight loss, the proportion mediated was generally modest, supporting predominantly weight-loss–independent biological effects. Although exenatide is no longer clinically used, these results suggest that the proteomic pathways we identified may mediate the improved cardiovascular outcomes seen with contemporary GLP-1 RA therapies in patients with and without T2DM and highlight the potential clinical and biological relevance of the proteins identified. More broadly, this work illustrates how high-throughput proteomics can be embedded within clinical trial populations to bridge biomarker prioritization and precision medicine implementation. Rather than focusing on isolated protein associations, we applied a structured analytic framework — feature selection, model development, external validation, and assessment of treatment responsiveness — to identify biologically coherent and clinically relevant protein signatures.

Our study has several strengths, including the use of a large, well-phenotyped clinical trial of a GLP-1 RA for discovery proteomics, alongside two validation cohorts: PROMISE, a symptom-focused cohort with no known ASCVD, and CHS, a population-based cohort. PROMISE in particular represents a population far earlier in the spectrum of disease development, and validation in this cohort is therefore of particular interest. Use of diverse cohorts supports both the robustness and generalizability of our findings. Importantly, we demonstrate that our protein score can be modified by a class of medications increasingly used for both primary and secondary prevention, highlighting its potential clinical utility. Although discovery was conducted in individuals with T2DM, our findings demonstrate that our protein score may also serve as a MACE biomarker in individuals without T2DM and in those without established ASCVD.

Key limitations include the strict exclusion criteria of the clinical trial, which may limit generalizability. Because this study was designed to identify biomarkers for MACE risk prediction, mechanistic inferences from proteomic associations should be interpreted as hypothesis-generating, and causal pathways cannot be established from these analyses. High-throughput aptamer-based proteomics also has inherent limitations. Platform coverage is broad but not comprehensive, and structural variants or posttranslational modifications may affect aptamer binding and measured signal. Additionally, circulating protein levels may not fully reflect tissue- or organ-specific biological processes. These considerations should inform interpretation of individual protein associations and pathway analyses. We also acknowledge that selecting significant proteins from single-protein Cox models is a screening strategy that may exclude proteins contributing only through conditional associations, interactions, or nonlinear relationships; future work could evaluate more efficient end-to-end penalized or nonlinear models trained on the full proteome. This limitation pertains principally to proteins we may have overlooked rather than to the validity of the proteins we identified, which were repeatedly prioritized across methods and externally validated. Accordingly, the Mendelian randomization and pathway analyses are interpreted as hypothesis-generating context for these prioritized proteins, while recognizing that additional causal candidates or pathways may exist among proteins not captured by our initial screen. Because our models adjust for clinical factors that may also function as mediators, some protein associations may be attenuated; accordingly, these findings should be interpreted primarily as biomarker associations for risk stratification rather than causal mechanisms. Although this study did not directly evaluate the mechanistic roles of the identified proteins, many have been previously characterized in prior studies (32, 34–37).

In conclusion, in a large, randomized controlled trial of a GLP-1 RA in patients with T2DM, we identified a novel protein score highly discriminative of MACE, which outperformed the use of clinical factors alone. This protein score and tetranectin alone was also predictive of MACE in broader populations and modifiable by a GLP-1 RA, supporting the clinical utility in using this score and biomarker to identify high-risk individuals who may have greater cardiovascular benefit from this class of medications. This study demonstrates the power of nesting high-throughput proteomics discovery in a large clinical drug trial to enable risk stratification and biomarker prioritization.

## Methods

### Sex as a biological variable

Our study examined male and female participants, and similar findings are reported for both sexes.

### Study population

#### EXSCEL trial.

The discovery cohort included participants with T2DM with biospecimens from the EXSCEL trial, a multicenter, double-blind, randomized controlled trial of once-weekly exenatide, a GLP-1 RA, versus a placebo. Trial design and IRB approvals were previously described (41). The trial enrolled 14,572 adults, 73% with a history of ASCVD. The primary outcome of the trial was time to first event of MACE (nonfatal myocardial infarction, stroke, or cardiovascular death). The EXSCEL trial demonstrated that exenatide did not reach superiority compared with a placebo for MACE but showed nominal 14% mortality reduction (42).

#### Proteomic profiling.

Plasma samples for proteomics were obtained at baseline and 1 year from a randomized subset of EXSCEL participants (*n* = 5,125) who had provided consent for future biomarker analyses and had available archived plasma at baseline and (when applicable) 12 months. Samples were collected according to the EXSCEL trial protocol, and participants were not required to be fasting. Proteomic data underwent SomaScan standard quality control (QC); samples failing QC metrics and proteins failing QC filters were excluded prior to analysis. Samples were stored at −80°C and assayed using SomaScan V4, which utilizes protein-specific aptamers that bind with high affinity (43). Protein levels were log_2_-transformed and standardized. After QC, 5,125 participants with 4,776 unique proteins remained.

### Validation cohorts

#### CHS.

The CHS was a prospective, population-based, longitudinal cohort study that enrolled adults 65 years or older in 1989–1990 to study risk factors for stroke and cardiovascular disease. Plasma from 3,188 individuals (1992–1993) was profiled with SomaScan V4, in which samples from 3,085 individuals were eligible for analysis (44). We validated the protein score in participants without ASCVD (*n* = 3,085), stratified by T2DM (*n* = 381) and non-T2DM (*n* = 2,382). Follow-up time was truncated at 5 years. The primary outcome was a composite of definite myocardial infarction, stroke, and cardiovascular death (45).

#### The PROMISE clinical trial.

PROMISE was a pragmatic trial of 10,003 outpatients with stable chest pain and no ASCVD, randomized to coronary CT angiography or usual care. This nested MACE case-control cohort (*n* = 860) conducted profiling via mass spectrometry–based proteomics. Outcomes included MACE and obstructive CAD. Protein associations were analyzed in T2DM (*n* = 178) and non-T2DM (*n* = 681) subgroups (46, 47).

### Statistics

#### Supervised machine learning models for prediction of MACE.

Approximately 5,000 individual baseline proteins were first assessed for association with time to MACE in multivariable Cox proportional hazard models adjusted for age, race, sex, treatment arm, BMI, estimated glomerular filtration rate (eGFR), systolic blood pressure, HbA1c, history of heart failure, and history of cardiovascular disease (ASCVD) in the EXSCEL discovery cohort. This initial screen was used as a dimension-reduction step to mitigate overfitting and improve stability in a high-dimensional, correlated proteomic feature space; downstream machine learning models were then trained using cross-validation within the retained feature set. We selected this conservative, interpretable screening strategy recognizing that it is one of several reasonable approaches and that, by relying on marginal associations, it may not capture proteins contributing primarily through conditional, interaction, or nonlinear effects. Proteins associated with MACE at FDR less than 0.05 were used as input variables in supervised machine learning models. LASSO, elastic net, random forest, and gradient-boosted trees were trained on 4,031 participants (79%) and tested on a held-out set of 1,094 (21%). Ten-fold cross-validation was used to tune hyperparameters. Hyperparameters were selected using cross-validation within the training set; all reported performance metrics (AUROC/AUPRC/calibration) are from the held-out test set. CIs for AUPRC were estimated using nonparametric bootstrap resampling of the held-out test set. To assess stability of protein prioritization in penalized regression, we performed bootstrap resampling (1,000 replicates) and refit the LASSO model in each replicate; stability was summarized as the proportion of replicates in which a protein had a non-zero coefficient. Analyses were conducted in R (v2024.04.1+748) using glmnet, caret, RandomForest, and xgboost (48–51).

#### Model protein importance analysis and protein score development.

Top-ranked proteins from machine learning models were evaluated across all model types, including their singular predictive performance with logistic regression. Logistic regression was used for individual protein prediction analyses to preserve mathematical alignment with the LASSO models, which estimate linear predictors on the log-odds scale; this approach enables direct comparison of single-protein predictive strength with the multi-protein LASSO-derived score without introducing differences attributable to time-to-event modeling. A protein score was developed using the sum of the LASSO model protein weights multiplied by protein levels and calculated per individual in the EXSCEL cohort. After computation, the protein score was standardized (*z* scored) within the relevant cohort (mean = 0, SD = 1) prior to association testing; hazard ratios therefore reflect risk per 1 SD increase in the standardized score. The protein score and clinical features were evaluated on the held-out test set using multivariable Cox proportional hazard models in time-to-MACE analyses.

#### The relationship between top-ranked proteins and BMI, HbA1c, and history of ASCVD.

To explore the biological link between top-ranked proteins and high-risk comorbidities for MACE, we analyzed their associations with baseline BMI, HbA1c, and ASCVD history in the EXSCEL cohort using Pearson’s correlation. One-way ANOVA was used to compare protein levels across ASCVD and MACE status groups, with post hoc analyses conducted via Tukey’s honestly significant difference testing.

#### Assessment of incremental risk prediction capabilities of protein score.

Clinical variables that aligned with prediction tools were used to evaluate the added prediction value of proteins (2, 3). These features included age, race, BMI, systolic blood pressure, diastolic blood pressure, duration of diabetes (years), ASCVD, history of atrial fibrillation, history of retinopathy, smoking status, HbA1c (%), total cholesterol, LDL-C, HDL cholesterol (HDL-C), high-sensitivity C-reactive protein (hsCRP), troponin I, eGFR, and current treatment with blood pressure mediation, aspirin, or statins. Models with input variables of protein only, clinical features only, and both proteins and clinical features were developed. The predictive contribution of top-ranked proteins was assessed using AUROC and AUPRC on the held-out test set.

#### Effects of exenatide on MACE proteins.

Linear mixed-effect models adjusted for change in BMI and HbA1c were used to assess differential change in top-ranked protein levels between baseline and 12 months in participants randomized to GLP-1 RA treatment versus placebo. We also performed a 12-month analysis among participants who were event-free at 12 months. Time-to-first MACE after 12 months was modeled by Cox regression with follow-up re-zeroed at 12 months. Protein score change was scaled using the baseline score mean and SD, and hazard ratios are reported per 1 SD decrease from baseline values. Models were adjusted for age, race, sex, randomization treatment, baseline BMI, baseline systolic blood pressure, baseline eGFR, baseline HbA1c, and baseline history of congestive heart failure and CAD. To assess whether treatment-associated changes in GLP-1 RA–modified proteins were mediated by weight loss, we performed causal mediation analyses using change in body weight from baseline to 12 months as the mediator. For each protein, we estimated the mediation effect, direct effect, total effect, and proportion mediated.

#### Validation studies.

Protein score validation was assessed using LASSO-derived weights from the EXSCEL cohort to compute protein scores in the CHS validation cohort. Associations with time to MACE were assessed via Cox proportional hazard models adjusted for clinical covariates (age, race, sex, diabetes, BMI, smoking, systolic blood pressure, LDL-C, HDL-C, and eGFR). Score tertiles were used to stratify Kaplan-Meier curves in both cohorts. Protein score performance was also evaluated in CHS T2DM and non-T2DM subsets as well as top-ranked individual proteins in both groups using adjusted Cox proportional hazard models.

Validation in the PROMISE study was limited to top-ranked individual proteins from EXSCEL due to differences in the utilized proteomic platforms. Associations with history of CAD (logistic regression) and time to MACE (Cox proportional hazard models) were assessed, adjusted for age, sex, race, hypertension, hyperlipidemia, smoking, eGFR, and statin use. Pearson’s correlations with CT angiography–adapted Leaman scores and 1-way ANOVA testing across T2DM, non-T2DM, MACE, and non-MACE subsets were conducted. Tukey’s honestly significant difference test was used to test post hoc group differences.

#### Mendelian randomization and gene set enrichment analysis.

Potential causal associations between top-ranked proteins and MACE components (nonfatal stroke, myocardial infarction, cardiovascular death) and ASCVD were explored using 2-sample cis-Mendelian randomization and published GWAS meta-analyses (52–55). Genetic instruments were selected at *P* less than 1 × 10^–6^ and clumped at *r*² < 0.1. Mendelian randomization estimates were generated using inverse-variance weighted models. Steiger filtering, outlier removal, and Mendelian randomization–Egger regression were applied to assess pleiotropy and multicollinearity. Analyses used the TwoSampleMR package in R (56). Pathway enrichment was assessed using baseline model *P* values to rank proteins (57). Fifty hallmark and 175 KEGG pathways were tested using the fgsea package (R v4.3.1) (58).A *P* value less than 0.05 was considered significant.

### Study approval

All study participants in EXSCEL, CHS, and PROMISE gave written informed consent for participation in the parent study and for use of their stored biospecimens for future use. The IRB for each site approved the primary studies and the Duke University IRB approved this biomarker substudy.

### Data availability

Data sets generated for these analyses are available from the corresponding author on reasonable request, subject to review and approval by the EXSCEL Study and Publications Committee. Data from the CHS and PROMIS studies are available on request to their respective Study and Publications Committees.

## Author contributions

KMC, MN, MYM, MER, and SHS designed the study. IZ performed and oversaw proteomics measurements in PROMISE. KMC, MN, and MYM performed statistical analyses. RJM, JBG, HS, GMF, NS, PSD, REG, RJB, AFH, RRH, BMP, JSF, and SHS provided expertise and oversight for EXSCEL, PROMISE, and CHS data. KMC, MN, MYM, and SHS wrote the manuscript; MER, HS, GMF, NS, JBG, PSD, REG, RJM, AFH, RRH, BMP, and JSF provided critical review and edits.

## Conflict of interest

NS received research support and Honoria from AstraZeneca, Boehringer Ingelheim, Novartis, Roche Diagnostics, Abbott Laboratories, AbbVie, Amgen, Lilly, and Novo Nordisk. PSD received consulting fees and honoraria from Amgen, Novo Nordisk, and UpToDate. GMF received research support and consulting fees from NIH, Bayer, BMS, Cytokinetics, Novartis, Merck, Otsuka, CSL Behring, Boehringer Ingelheim, Myovant Sciences, River2Renal, Roche Diagnostics, and WhiteSwell. SHS received research support from AstraZeneca and Lilly. HS received research support, consulting fees, and honoraria from the European Union, Austrian Science Fund, Amarin, Amgen, Bayer, Boehringer Ingelheim, Daiichi Sankyo, Lilly, NovoNordisk, and Sanofi. RJM received research support from AstraZeneca, Lilly, and NovoNordisk. AFH received research support and consulting fees from AstraZeneca, Bristol Myers Squibb, and Boehringer Ingelheim. RRH received consulting fees and honoraria from MitoRx, Novartis, Owen Mumford Ltd, Lilly, and Merck. JG received research support and consulting fees from AstraZeneca, Boehringer Ingelheim, Lilly, Merck, Roche, Bluedrop, Bayer, NovoNordisk, Anji, Vertex, Valo, Mineralys, and Corcept.

## Funding support

This work is the result of NIH funding, in whole or in part, and is subject to the NIH Public Access Policy. Through acceptance of this federal funding, the NIH has been given a right to make the work publicly available in PubMed Central.

- Amylin Pharmaceuticals to the EXSCEL study.
- National Heart, Lung, and Blood Institute (NHLBI) contracts HHSN268201200036C, HHSN268200800007C, HHSN268201800001C, N01HC55222, N01HC85079, N01HC85080, N01HC85081, N01HC85082, N01HC85083, N01HC85086, and 75N92021D00006, and NHLBI grants R01HL146145, U01HL080295, U01HL130114, R01HL172803, and R01HL144483,
- National Institute of Neurological Disorders and Stroke (NINDS).
- National Institute on Aging grant R01AG023629.

## Supplementary Material

Supplemental data

ICMJE disclosure forms

Supporting data values

## Figures and Tables

**Table 1:**
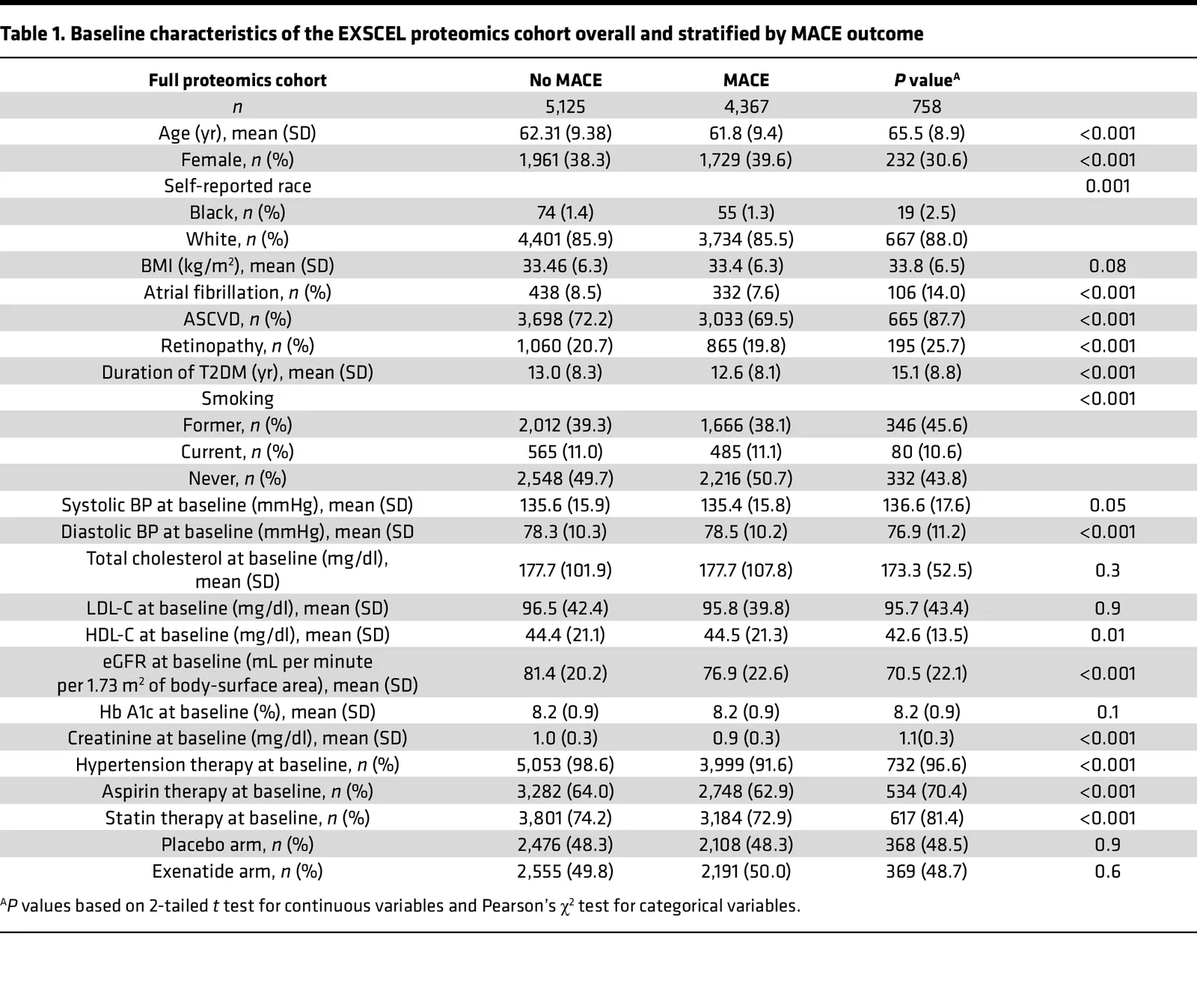
Baseline characteristics of the EXSCEL proteomics cohort overall and stratified by MACE outcome

**Table 2:**
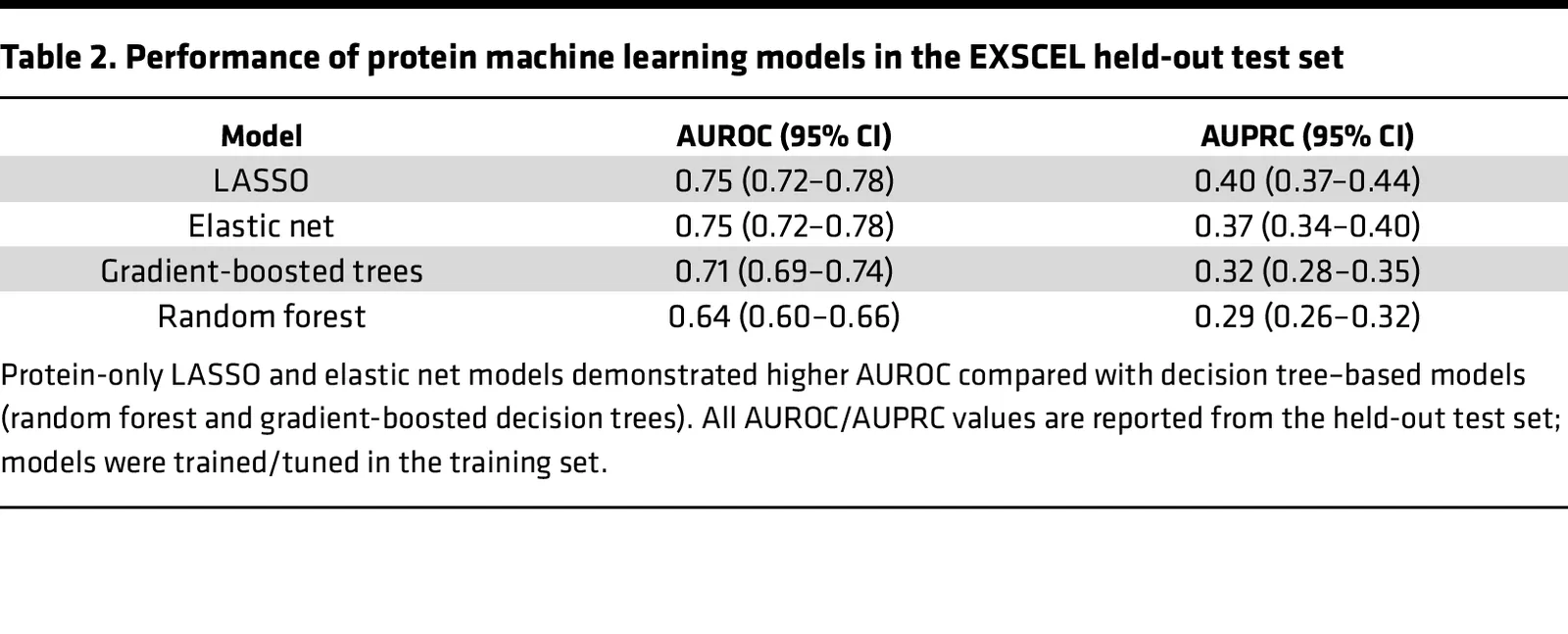
Performance of protein machine learning models in the EXSCEL held-out test set

**Table 3:**
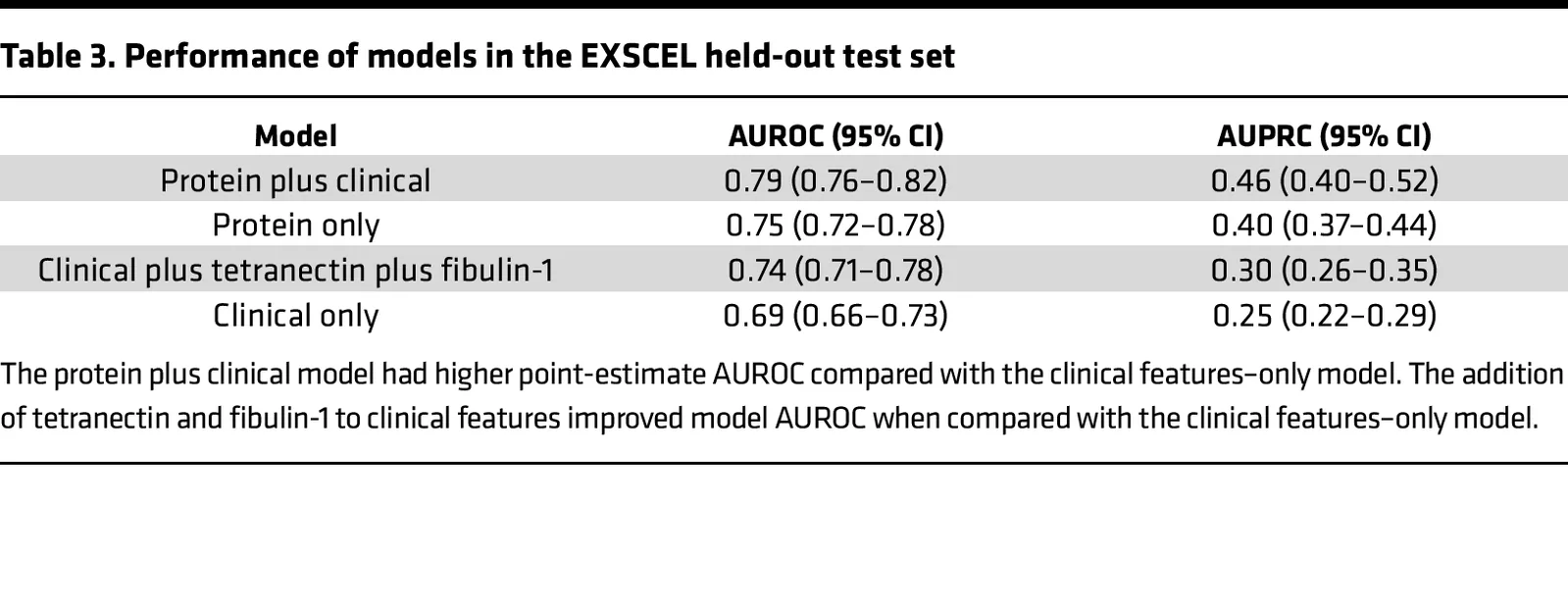
Performance of models in the EXSCEL held-out test set

**Table 4:**
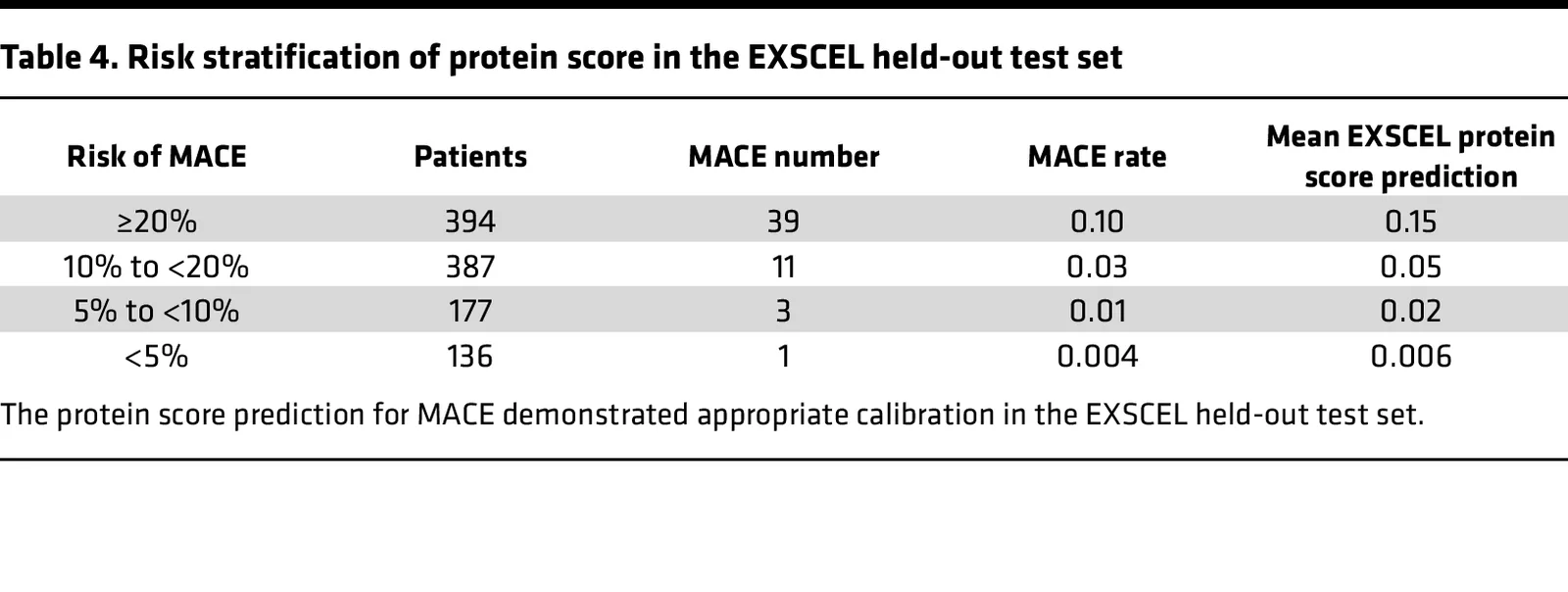
Risk stratification of protein score in the EXSCEL held-out test set

**Table 5:**
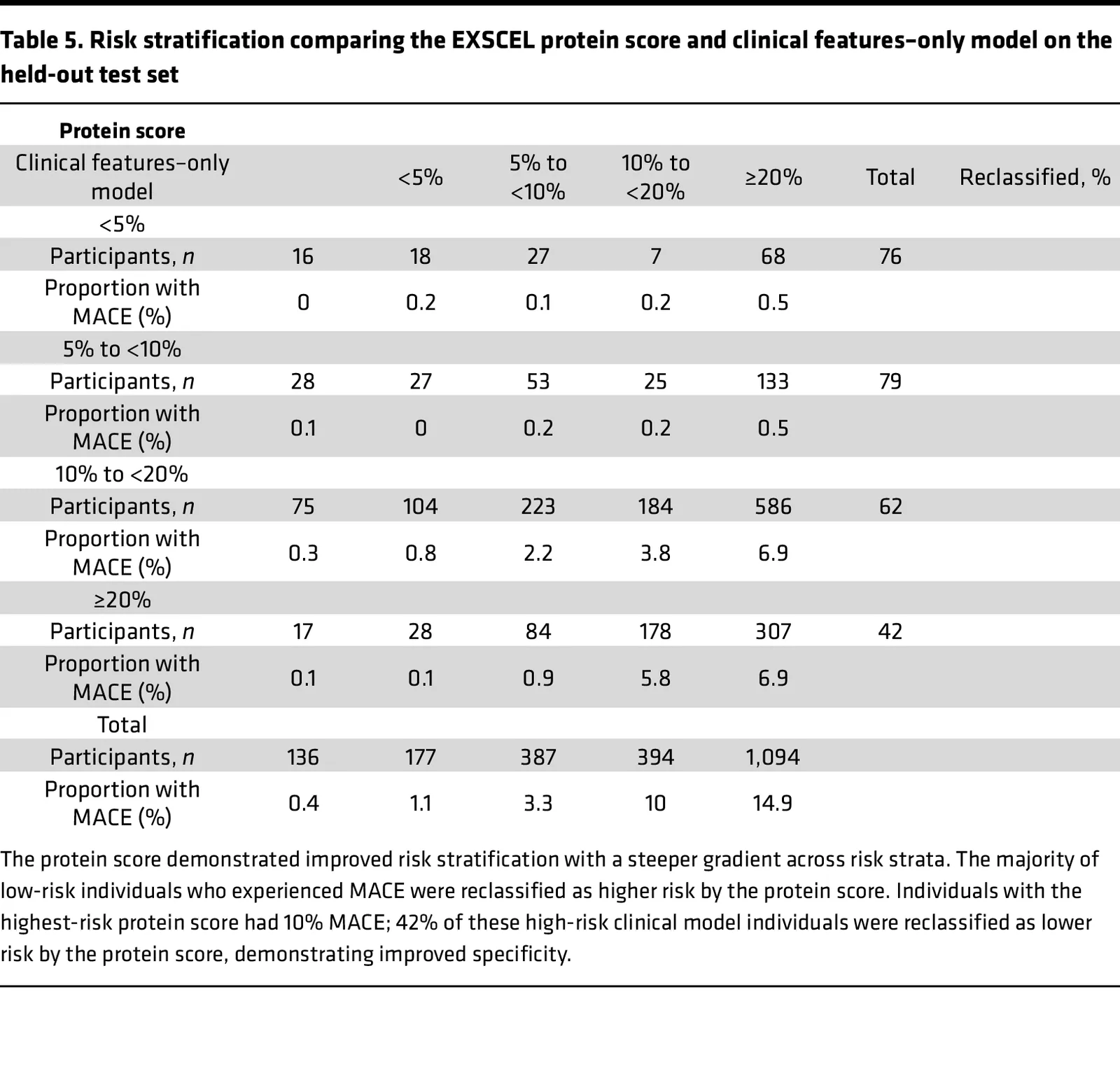
Risk stratification comparing the EXSCEL protein score and clinical features–only model on the held-out test set

## Version 1 07/02/2026

In-Press Preview

## Version 2 08/24/2026

Electronic publication
