# Supplementary material for: A validated, modifiable proteomic score from the EXSCEL trial predicts cardiovascular events in diabetes
Source: JCI Insight. 2026 Jul 2;11(16):e204463. doi: 10.1172/jci.insight.204463 (PMC13502179; doi:10.1172/jci.insight.204463)
Supplement: Supplementary file 1 [file jciinsight-11-204463-s062.pdf]

Supplemental Table 1: *Baseline characteristics of the EXSCEL clinical trial cohort that did not have proteomics versus EXSCEL proteomics cohort selected at random.*

	EXSCEL Cohort without Proteomics	EXSCEL Cohort with Proteomics	*p
n	9627	5125	
Age (mean (SD))	61.65 (9.40)	62.31 (9.38)	<0.001
Sex = M (%)	5985 (62.2)	3164 (61.7)	0.619
Pooled race group (%)			<0.001
Black	804 (8.4)	74 (1.4)	
White	6774 (70.4)	4401 (85.9)	
Treatment Arm (%)			0.004
EQW 2 mg	4789 (49.7)	2555 (49.9)	
Not Treated	33 (0.3)	3 (0.1)	
Placebo	4805 (49.9)	2567 (50.1)	
Body Mass Index (mean (SD))	32.26 (6.40)	33.46 (6.32)	<0.001
Atrial fibrillation/atrial flutter (%)	561 (5.8)	438 (8.5)	<0.001
Prior CV Event (for stratification) = Y (%)	7084 (73.6)	3698 (72.2)	0.065
Duration of Diabetes at BL (mean (SD))	13.13 (8.26)	13.00 (8.29)	0.347
Smoking Status at BL (%)			0.172
Current	1156 (12.0)	565 (11.0)	
Former	3779 (39.3)	2012 (39.3)	
Never	4685 (48.7)	2548 (49.7)	
Systolic BP at BL (mean (SD))	135.39 (17.36)	135.59 (15.95)	0.512
Diastolic BP at Baseline (mean (SD))	78.04 (10.26)	78.26 (10.29)	0.212
Total Cholesterol at Baseline (mg/dl) (mean (SD))	173.81 (140.93)	177.65 (101.94)	0.094
LDL at Baseline (mg/dl) (mean (SD))	94.14 (62.75)	96.46 (42.35)	0.032
HDL at Baseline (mg/dl) (mean (SD))	43.77 (27.92)	44.36 (21.12)	0.211
eGFR at Baseline (mean (SD))	81.62 (20.37)	81.40 (20.19)	0.54
Hb A1c at Baseline (mean (SD))	8.09 (0.97)	8.15 (0.94)	<0.001
Creatinine at Baseline (mg/dl) (mean (SD))	0.98 (0.29)	0.98 (0.28)	0.497
Hypertension Therapy at Baseline = Y (%)	9471 (98.4)	5053 (98.6)	0.347
Aspirin Therapy at Baseline = Y (%)	6098 (63.3)	3282 (64.0)	0.413
Statin Therapy at Baseline = Y (%)	7044 (73.2)	3801 (74.2)	0.198

*P-values based on t-test for continuous variables and Pearson's chi-squared test for categorical variables.

Supplemental Table 2: *Baseline characteristics of validation cohort CHS overall and stratified by baseline CVD.*

	Full Proteomics Cohort	No Prevalent CVD	CVD at Baseline	*p-value
N	3,085	2,763	322	
Age (mean (SD))	74.4 (4.9)	74.3 (4.8)	75.0 (5.1)	0.012
Female, N (%)	1,875 (60.8)	1,730 (62.6)	145 (45.0)	<0.001
Self-reported Race				0.024
Black, N (%)	495 (16.0)	423 (15.3)	72 (22.4)	
Other, N (%)	16 (0.5)	15 (0.5)	1 (0.3)	
White, N (%)	2,574 (83.4)	2,325 (84.1)	240 (83.9)	
BMI (mean (SD))	26.6 (4.5)	26.6 (4.5)	26.6 (4.5)	0.721
DM, N (%)	457 (14.8)	381 (13.8)	76 (23.6)	<0.001
Smoking				0.059
Former, N (%)	1,360 (44.1)	1,198 (43.4)	162 (50.3)	
Current, N (%)	291 (9.4)	264 (9.6)	27 (8.4)	
Never, N (%)	1,434 (46.5)	1,301 (47.1)	133 (41.3)	
Systolic BP at Baseline (mean (SD))	135.6 (21.2)	135.5 (21.0)	136.5 (22.7)	0.520
Diastolic BP at Baseline (mean (SD))	71.3 (10.9)	71.4 (10.8)	70.2 (11.3)	0.149
Total Cholesterol at Baseline (mg/dl) (mean (SD))	202.4 (37.2)	202.4 (36.5)	202.9 (42.8)	0.659
LDL at Baseline (mg/dl) (mean (SD))	121.1 (33.6)	120.8 (33.0)	123.6 (38.5)	0.386
HDL at Baseline (mg/dl) (mean (SD))	54.1 (14.2)	54.6 (14.2)	49.9 (13.7)	<0.001
eGFR at Baseline (mean (SD))	70.2 (16.3)	70.8 (15.9)	65.4 (19.2)	<0.001
Aspirin Therapy at Baseline, N (%)	90 (2.9)	69 (2.5)	21 (6.5)	<0.001
Statin Therapy at Baseline, N (%)	160 (5.2)	127 (4.6)	33 (10.2)	<0.001

*P-values based on t-test for continuous variables and Pearson's chi-squared test for categorical variables.

Supplemental Table 3: *Baseline characteristics of validation cohort PROMISE overall and stratified by MACE.*

	Full Validation Cohort	No MACE	MACE	*p-value
n	860	821	38	
Age (mean (SD))	59.7 (8.1)	59.6 (8.1)	61.9 (9.4)	0.1
Female (%)	286 (33.3)	276 (33.6)	10 (26.3)	0.4
Race (%)				0.6
American Indian, N (%)	4 (0.5)	4 (0.5)	0 (0.0)	
White, N (%)	771 (90.2)	738 (90.3)	33 (86.8)	
Black, N (%)	65 (7.6)	60 (7.3)	5 (13.2)	
Asian, N (%)	11 (1.3)	11 (1.3)	0 (0.0)	
Pacific Islander, N (%)	4 (0.5)	4 (0.5)	0 (0.0)	
BMI (mean (SD))	30.52 (5.7)	30.60 (5.7)	28.89 (4.0)	0.08
Smoker (%)				0.03
Never, N (%)	375 (43.7)	366 (44.6)	9 (23.7)	
Current, N (%)	185 (21.5)	176 (21.4)	9 (23.7)	
Former, N (%)	299 (34.8)	279 (34.0)	20 (52.6)	
History of T2DM (%)	178 (20.7)	170 (20.7)	8 (21.1)	1
History of HTN (%)	562 (65.4)	540 (65.8)	22 (57.9)	0.4
History of CVD (%)	32 (3.7)	32 (3.9)	0 (0.0)	0.4
History of HF (%)	46 (5.4)	44 (5.4)	2 (5.3)	1
History of HLD (%)	554 (64.5)	531 (64.7)	23 (60.5)	0.7

eGFR (mean (SD))	80.14 (18.9)	80.07 (18.8)	81.66 (21.6)	0.6
Statin therapy at Baseline (%)	366 (44.8)	351 (44.9)	15 (41.7)	0.8

*P-values based on t-test for continuous variables and Pearson's chi-squared test for categorical variables.

Supplement Table 4: *Significant proteins from multivariable Cox proportional hazards models associated with MACE in EXSCEL proteomics cohort (N=1,278)*.

Protein	HR	Lower Confidence Limit	Upper Confidence Limit	Standard Error	p-value	FDR adjusted p-value
**Tetranectin**	0.165	0.121	0.227	0.16	3.56E-29	8.86E-26
**Tumor necrosis factor ligand superfamily member 15**	3.877	3.032	4.957	0.125	3.19E-27	5.30E-24
**Angiopoietin-2**	2.53	2.114	3.029	0.0918	4.86E-24	6.04E-21
**Natriuretic peptides B**	1.879	1.638	2.155	0.07	1.99E-19	1.98E-16
**Lipopolysaccharide-binding protein**	9.003	5.564	14.568	0.246	3.58E-19	2.97E-16
**Coiled-coil domain-containing protein 126**	0.308	0.237	0.399	0.133	7.96E-19	5.66E-16
**Alpha-1-antichymotrypsin**	11.221	6.561	19.191	0.274	1.04E-18	6.46E-16
**Collagen alpha-1(XXVIII) chain**	3.786	2.783	5.151	0.157	2.38E-17	1.24E-14
**Palmitoleoyl-protein carboxylesterase NOTUM**	0.456	0.38	0.547	0.0927	2.50E-17	1.24E-14
**Protein jagged-1**	2.482	2.009	3.067	0.108	3.50E-17	1.59E-14
**Group XIIB secretory phospholipase A2-like protein**	0.313	0.239	0.411	0.138	4.87E-17	2.02E-14
**Hemopexin**	7.822	4.82	12.692	0.247	8.28E-17	3.17E-14
**Neuroblastoma suppressor of tumorigenicity 1**	2.088	1.751	2.49	0.0898	2.40E-16	6.72E-14
**WAP four-disulfide core domain protein 2**	2.586	2.061	3.244	0.116	2.18E-16	6.72E-14
**Fibulin-1**	6.739	4.275	10.623	0.232	2.10E-16	6.72E-14
**Ficolin-1**	1.821	1.579	2.1	0.0729	1.94E-16	6.72E-14
**Immunoglobulin G**	7.097	4.443	11.338	0.239	2.43E-16	6.72E-14
**Beta-2-microglobulin**	3.461	2.561	4.678	0.154	6.43E-16	1.68E-13
**Alpha-2-HS-glycoprotein**	0.142	0.088	0.229	0.243	1.08E-15	2.69E-13
**Sushi, von Willebrand factor type A, EGF and pentraxin domain-containing protein 1**	2.164	1.788	2.618	0.0973	2.09E-15	4.96E-13
**Follistatin-related protein 3**	3.48	2.552	4.745	0.158	3.18E-15	7.19E-13
**Complement component C9**	4.699	3.163	6.979	0.202	1.77E-14	3.84E-12
**Asialoglycoprotein receptor 1**	2.661	2.071	3.42	0.128	1.96E-14	4.07E-12
**Cadherin-3**	0.249	0.174	0.357	0.184	3.93E-14	7.84E-12
**EGF-containing fibulin-like extracellular matrix protein 1**	3.012	2.25	4.032	0.149	1.25E-13	2.39E-11
**Epidermal growth factor receptor**	0.135	0.08	0.23	0.27	1.35E-13	2.49E-11
**Collagen alpha-3(VI) chain**	3.205	2.35	4.371	0.158	1.89E-13	3.35E-11
**Cholinesterase**	0.303	0.22	0.416	0.163	2.08E-13	3.57E-11
**Extracellular tyrosine-protein kinase PKDCC**	0.204	0.133	0.312	0.217	2.30E-13	3.82E-11
**Zinc finger protein 18**	0.127	0.073	0.222	0.286	4.85E-13	7.78E-11
**Sodium-coupled monocarboxylate transporter 1**	2.308	1.838	2.898	0.116	5.87E-13	9.14E-11

**Ganglioside GM2 activator**	2.713	2.066	3.563	0.139	6.97E-13	1.05E-10
**Tumor necrosis factor receptor superfamily member 1A**	2.451	1.914	3.139	0.126	1.22E-12	1.78E-10
**Alpha-internexin**	1.775	1.515	2.079	0.0808	1.25E-12	1.78E-10
**Cystatin-C**	3.511	2.48	4.971	0.177	1.46E-12	1.96E-10
**R-spondin-4**	2.316	1.835	2.921	0.119	1.42E-12	1.96E-10
**Insulin-like growth factor-binding protein 2**	1.854	1.562	2.2	0.0874	1.62E-12	2.07E-10
**Fibulin-5**	2.902	2.16	3.899	0.151	1.58E-12	2.07E-10
**Copper transport protein ATOX1**	1.841	1.552	2.184	0.0873	2.66E-12	3.31E-10
**E3 ubiquitin-protein ligase DTX1**	0.258	0.176	0.377	0.194	2.99E-12	3.63E-10
**Syndecan-3**	0.378	0.287	0.497	0.14	3.60E-12	4.27E-10
**Selenoprotein M**	2.487	1.918	3.223	0.132	6.03E-12	6.98E-10
**Sushi, von Willebrand factor type A, EGF and pentraxin domain-containing protein 1**	1.896	1.579	2.277	0.0934	7.28E-12	7.97E-10
**Alcohol dehydrogenase 1A**	0.578	0.494	0.676	0.08	7.19E-12	7.97E-10
**Peflin**	3.016	2.199	4.136	0.161	7.36E-12	7.97E-10
**Serotransferrin**	6.723	3.888	11.624	0.279	9.05E-12	9.58E-10
**Growth hormone receptor**	0.421	0.328	0.54	0.128	1.23E-11	1.25E-09
**Surfeit locus protein 1**	0.487	0.396	0.6	0.106	1.22E-11	1.25E-09
**Plastin-2**	0.368	0.276	0.492	0.148	1.31E-11	1.30E-09
**Uncharacterized protein C3orf18**	0.199	0.125	0.318	0.239	1.49E-11	1.45E-09
**Matrix-remodeling-associated protein 7**	2.073	1.676	2.564	0.109	1.83E-11	1.75E-09
**Intercellular adhesion molecule 5**	2.106	1.691	2.623	0.112	2.98E-11	2.80E-09
**Endoglin**	2.504	1.909	3.286	0.139	3.40E-11	3.13E-09
**Transgelin**	2.212	1.749	2.799	0.12	3.73E-11	3.37E-09
**Cartilage acidic protein 1**	0.409	0.314	0.534	0.136	4.66E-11	4.14E-09
**Ectonucleoside triphosphate diphosphohydrolase 5**	0.383	0.287	0.511	0.147	7.68E-11	6.70E-09
**Integral membrane protein 2B**	1.808	1.511	2.162	0.0913	9.00E-11	7.72E-09
**Acidic leucine-rich nuclear phosphoprotein 32 family member B**	2.06	1.652	2.567	0.112	1.29E-10	1.09E-08
**Complement factor H**	16.573	7.037	39.031	0.437	1.32E-10	1.10E-08
**Spondin-1**	2.906	2.098	4.025	0.166	1.39E-10	1.13E-08
**Alcohol dehydrogenase 4**	0.677	0.6	0.762	0.061	1.46E-10	1.17E-08
**Shadow of prion protein**	1.668	1.426	1.951	0.08	1.62E-10	1.28E-08
**Delta-like protein 4**	1.968	1.598	2.423	0.106	1.89E-10	1.47E-08
**Uncharacterized protein C14orf93**	0.339	0.243	0.474	0.17	2.20E-10	1.69E-08

**Zona pellucida-binding protein 1**	0.114	0.058	0.223	0.342	2.30E-10	1.73E-08
**Growth/differentiation factor 15**	1.695	1.438	1.998	0.0839	3.26E-10	2.42E-08
**Ephrin-B2**	2.311	1.778	3.005	0.134	4.02E-10	2.94E-08
**tRNA (guanine-N(7)-)-methyltransferase**	0.219	0.136	0.353	0.244	4.60E-10	3.32E-08
**Chymotrypsin-like elastase family member 1**	1.886	1.544	2.304	0.102	5.01E-10	3.57E-08
**Hippocalcin-like protein 1**	0.308	0.213	0.447	0.19	5.25E-10	3.68E-08
**Kallikrein-11**	2.402	1.818	3.174	0.142	7.11E-10	4.91E-08
**Titin**	1.565	1.357	1.805	0.0728	7.85E-10	5.36E-08
**Inactive tyrosine-protein kinase 7**	3.137	2.178	4.518	0.186	8.19E-10	5.51E-08
**Periostin**	2.07	1.64	2.611	0.119	8.71E-10	5.73E-08
**Sodium/potassium-transporting ATPase subunit beta-1**	0.53	0.432	0.649	0.104	8.75E-10	5.73E-08
**DnaJ homolog subfamily B member 12**	1.83	1.506	2.222	0.0992	1.13E-09	7.11E-08
**Solute carrier family 22 member 16**	0.564	0.469	0.678	0.0942	1.13E-09	7.11E-08
**Biglycan**	2.057	1.631	2.594	0.118	1.12E-09	7.11E-08
**C-reactive protein**	1.467	1.296	1.66	0.0631	1.25E-09	7.77E-08
**Alcohol dehydrogenase 1C**	0.753	0.687	0.826	0.0468	1.42E-09	8.70E-08
**WAP four-disulfide core domain protein 1**	2.231	1.72	2.894	0.133	1.46E-09	8.86E-08
**Troponin T, cardiac muscle**	1.547	1.343	1.783	0.0723	1.60E-09	9.61E-08
**Latent-transforming growth factor beta-binding protein 4**	2.288	1.747	2.995	0.137	1.74E-09	1.03E-07
**Growth factor receptor-bound protein 7**	0.247	0.156	0.39	0.234	2.11E-09	1.24E-07
**Resistin**	1.987	1.586	2.49	0.115	2.46E-09	1.42E-07
**Alpha-1-microglobulin**	4.355	2.682	7.07	0.247	2.67E-09	1.53E-07
**Roundabout homolog 1**	2.176	1.684	2.811	0.131	2.74E-09	1.55E-07
**Alpha-N-acetylgalactosaminide alpha-2,6-sialyltransferase 2**	0.472	0.368	0.605	0.127	2.95E-09	1.63E-07
**Intercellular adhesion molecule 5**	2.094	1.64	2.673	0.125	2.95E-09	1.63E-07
**Lymphotoxin alpha2:beta1**	0.371	0.268	0.515	0.167	2.98E-09	1.63E-07
**rRNA 2'-O-methyltransferase fibrillarin**	0.259	0.165	0.406	0.23	3.98E-09	2.15E-07
**Protocadherin-10**	1.806	1.483	2.2	0.101	4.13E-09	2.20E-07
**Plasminogen**	0.243	0.152	0.39	0.24	4.16E-09	2.20E-07
**Apolipoprotein B**	1.749	1.451	2.11	0.0956	4.90E-09	2.57E-07
**Vascular endothelial growth factor D**	1.844	1.501	2.265	0.105	5.60E-09	2.90E-07
**Ephrin-A5**	2.444	1.809	3.303	0.154	5.82E-09	2.99E-07
**Twisted gastrulation protein homolog 1**	3.18	2.149	4.704	0.2	7.06E-09	3.59E-07
**Scavenger receptor class F member 2**	3.323	2.211	4.994	0.208	7.55E-09	3.80E-07

**Cadherin-7**	0.57	0.471	0.69	0.0975	8.06E-09	3.96E-07
**C-type lectin domain family 4 member G**	1.895	1.525	2.355	0.111	8.04E-09	3.96E-07
**Torsin-1A-interacting protein 1**	0.51	0.405	0.641	0.117	8.19E-09	3.96E-07
**Apolipoprotein A-I**	0.523	0.419	0.652	0.112	8.15E-09	3.96E-07
**Heat shock 70 kDa protein 1A**	0.377	0.27	0.525	0.169	8.42E-09	4.03E-07
**Selenoprotein W**	1.634	1.381	1.933	0.0857	1.02E-08	4.79E-07
**Trefoil factor 3**	1.993	1.574	2.524	0.12	1.02E-08	4.79E-07
**Ran-binding protein 3**	1.9	1.525	2.368	0.112	1.04E-08	4.84E-07
**Hexokinase-1**	0.362	0.255	0.513	0.178	1.13E-08	5.20E-07
**Trefoil factor 2**	1.66	1.395	1.975	0.0888	1.14E-08	5.21E-07
**Leukocyte immunoglobulin-like receptor subfamily A member 5**	2.042	1.597	2.61	0.125	1.20E-08	5.45E-07
**Thioredoxin-interacting protein**	0.412	0.304	0.56	0.156	1.37E-08	6.17E-07
**Mannan-binding lectin serine protease 1**	0.221	0.131	0.373	0.267	1.50E-08	6.69E-07
**Alpha-2-antiplasmin**	0.388	0.279	0.538	0.168	1.54E-08	6.77E-07
**Serum amyloid A-1 protein**	1.26	1.163	1.366	0.0409	1.57E-08	6.86E-07
**Rho GTPase-activating protein 1**	1.862	1.501	2.31	0.11	1.60E-08	6.90E-07
**Fructose-bisphosphate aldolase C**	0.517	0.411	0.65	0.117	1.61E-08	6.90E-07
**Kininogen-1**	0.228	0.137	0.382	0.262	1.72E-08	7.32E-07
**Potassium voltage-gated channel subfamily E regulatory beta subunit 5**	0.352	0.245	0.507	0.185	1.78E-08	7.52E-07
**Glycerol-3-phosphate dehydrogenase [NAD(+)], cytoplasmic**	0.663	0.574	0.765	0.0732	1.82E-08	7.63E-07
**Pyrroline-5-carboxylate reductase 2**	0.277	0.177	0.433	0.228	1.85E-08	7.67E-07
**CD27 antigen**	0.414	0.304	0.563	0.157	1.92E-08	7.88E-07
**Protein Wnt-3a**	0.48	0.371	0.62	0.131	2.10E-08	8.57E-07
**Vesicular integral-membrane protein VIP36**	2.72	1.915	3.864	0.179	2.26E-08	9.14E-07
**Adhesion G protein-coupled receptor E2**	0.618	0.522	0.732	0.0861	2.30E-08	9.23E-07
**Leucine-rich repeat-containing protein 74A**	0.296	0.194	0.454	0.218	2.32E-08	9.25E-07
**Tenascin**	2.362	1.747	3.193	0.154	2.37E-08	9.36E-07
**Macrophage metalloelastase**	1.56	1.334	1.823	0.0797	2.43E-08	9.52E-07
**Fibrinogen-like protein 1**	1.514	1.308	1.752	0.0745	2.61E-08	1.01E-06
**Valine--tRNA ligase**	0.476	0.366	0.618	0.134	2.67E-08	1.03E-06
**Ephrin type-A receptor 2**	1.996	1.564	2.546	0.124	2.74E-08	1.05E-06
**Follistatin-related protein 1**	4.46	2.628	7.57	0.27	3.04E-08	1.16E-06
**Liver carboxylesterase 1**	0.482	0.372	0.624	0.132	3.07E-08	1.16E-06
**Fructose-bisphosphate aldolase B**	0.69	0.605	0.787	0.0673	3.45E-08	1.29E-06

**Marginal zone B- and B1-cell-specific protein**	1.639	1.375	1.954	0.0897	3.66E-08	1.36E-06
**Iron-sulfur cluster co-chaperone protein HscB, mitochondrial**	0.397	0.286	0.551	0.168	3.70E-08	1.36E-06
**Endothelial differentiation-related factor 1**	0.412	0.3	0.565	0.161	3.76E-08	1.37E-06
**Carbohydrate sulfotransferase 12**	2.191	1.657	2.897	0.143	3.76E-08	1.37E-06
**Acyl-CoA-binding domain-containing protein 6**	2.341	1.729	3.169	0.155	3.79E-08	1.37E-06
**CD59 glycoprotein**	2.101	1.612	2.737	0.135	3.88E-08	1.39E-06
**Hemoglobin subunit delta**	0.345	0.236	0.505	0.194	4.08E-08	1.45E-06
**Regulator of G-protein signaling 8**	0.406	0.294	0.56	0.165	4.23E-08	1.49E-06
**Tumor necrosis factor ligand superfamily member 14**	0.327	0.219	0.488	0.204	4.38E-08	1.53E-06
**Protein delta homolog 2**	1.736	1.425	2.116	0.101	4.62E-08	1.61E-06
**Interleukin-1 receptor type 2**	0.386	0.274	0.544	0.175	5.06E-08	1.75E-06
**Aminoacylase-1**	0.708	0.626	0.802	0.0633	5.18E-08	1.78E-06
**Peroxidasin homolog**	1.594	1.347	1.886	0.086	5.98E-08	2.04E-06
**Apolipoprotein F**	1.569	1.333	1.847	0.0832	6.10E-08	2.07E-06
**Tyrosine-protein kinase transmembrane receptor ROR2**	1.811	1.46	2.246	0.11	6.46E-08	2.17E-06
**Alpha-1,6-mannosyl-glycoprotein 2-beta-N-acetylglucosaminyltransferase**	0.295	0.189	0.459	0.226	6.60E-08	2.21E-06
**SAM pointed domain-containing Ets transcription factor**	0.254	0.154	0.418	0.254	6.94E-08	2.30E-06
**Pulmonary surfactant-associated protein D**	1.49	1.289	1.722	0.074	7.19E-08	2.37E-06
**Cyclic AMP-dependent transcription factor ATF-6 alpha**	0.375	0.263	0.537	0.182	7.67E-08	2.50E-06
**Tumor necrosis factor receptor type 1-associated DEATH domain protein**	0.551	0.443	0.685	0.111	7.69E-08	2.50E-06
**Transcription regulator protein BACH1**	0.351	0.24	0.515	0.195	8.00E-08	2.59E-06
**Ras-related protein Rab-17**	0.467	0.354	0.617	0.142	8.08E-08	2.60E-06
**Lysozyme C**	1.766	1.435	2.174	0.106	8.19E-08	2.61E-06
**OTU domain-containing protein 5**	0.566	0.46	0.697	0.106	8.51E-08	2.70E-06
**Complement component C1q receptor**	2.689	1.87	3.867	0.185	9.46E-08	2.96E-06
**Inhibin beta A chain:Inhibin beta B chain heterodimer**	1.826	1.464	2.277	0.113	9.41E-08	2.96E-06
**Biotinidase**	0.376	0.262	0.539	0.184	9.94E-08	3.09E-06
**Guanylate-binding protein 6**	0.422	0.307	0.579	0.162	1.01E-07	3.12E-06
**Apolipoprotein A-IV**	0.668	0.576	0.775	0.0757	1.02E-07	3.12E-06
**Neuropeptide S**	0.513	0.401	0.657	0.126	1.15E-07	3.50E-06
**Endothelin-converting enzyme 1**	0.303	0.194	0.471	0.226	1.19E-07	3.62E-06
**Glutaminyl-peptide cyclotransferase**	1.655	1.373	1.995	0.0952	1.21E-07	3.64E-06
**Growth arrest-specific protein 1**	2.004	1.549	2.593	0.131	1.22E-07	3.67E-06
**Heat shock 70 kDa protein 6**	0.199	0.11	0.363	0.305	1.24E-07	3.69E-06

**Fc receptor-like protein 6**	0.314	0.205	0.483	0.219	1.26E-07	3.74E-06
**Syntaxin-binding protein 6**	0.504	0.39	0.65	0.13	1.36E-07	4.00E-06
**IGF-like family receptor 1**	1.54	1.311	1.808	0.0819	1.39E-07	4.07E-06
**Alpha-N-acetylgalactosaminide alpha-2,6-sialyltransferase 1**	0.398	0.282	0.561	0.175	1.41E-07	4.11E-06
**SLAM family member 8**	1.456	1.265	1.674	0.0714	1.47E-07	4.25E-06
**B melanoma antigen 3**	0.514	0.401	0.659	0.127	1.51E-07	4.36E-06
**Leucine-rich repeat-containing G-protein coupled receptor 5**	0.31	0.2	0.48	0.223	1.52E-07	4.36E-06
**Protocadherin gamma-C3**	0.658	0.562	0.769	0.0801	1.65E-07	4.58E-06
**14-3-3 protein gamma**	1.861	1.475	2.348	0.119	1.64E-07	4.58E-06
**Zinc finger protein 175**	0.257	0.154	0.427	0.26	1.62E-07	4.58E-06
**Interleukin-37**	0.49	0.376	0.64	0.136	1.63E-07	4.58E-06
**Cysteine-rich PDZ-binding protein**	0.397	0.28	0.561	0.177	1.64E-07	4.58E-06
**Histone-lysine N-methyltransferase EHMT2**	0.487	0.372	0.638	0.137	1.65E-07	4.58E-06
**Cytochrome c oxidase subunit 4 isoform 2, mitochondrial**	0.394	0.278	0.559	0.178	1.67E-07	4.60E-06
**Myelin regulatory factor**	0.309	0.199	0.48	0.225	1.71E-07	4.67E-06
**Carboxypeptidase E**	0.329	0.216	0.499	0.213	1.75E-07	4.76E-06
**Phosphoenolpyruvate carboxykinase, cytosolic [GTP]**	0.443	0.326	0.602	0.156	1.91E-07	5.17E-06
**Apolipoprotein M**	0.412	0.295	0.575	0.17	1.97E-07	5.30E-06
**Fructose-1,6-bisphosphatase 1**	0.691	0.6	0.794	0.0713	2.10E-07	5.61E-06
**Anthrax toxin receptor 2**	0.543	0.431	0.684	0.118	2.19E-07	5.79E-06
**Protein shisa-2 homolog**	0.381	0.264	0.549	0.186	2.19E-07	5.79E-06
**Retinoblastoma-like protein 2**	1.862	1.472	2.355	0.12	2.20E-07	5.80E-06
**Suppressor of fused homolog**	0.24	0.14	0.413	0.276	2.37E-07	6.19E-06
**Tenascin**	1.881	1.48	2.39	0.122	2.37E-07	6.19E-06
**Chymotrypsin-like protease CTRL-1**	0.435	0.317	0.597	0.161	2.44E-07	6.33E-06
**Metalloproteinase inhibitor 1**	2.292	1.672	3.14	0.161	2.48E-07	6.40E-06
**Fatty acid-binding protein, adipocyte**	1.685	1.382	2.055	0.101	2.58E-07	6.63E-06
**Rho GTPase-activating protein 1**	1.476	1.272	1.713	0.0759	2.82E-07	7.20E-06
**CD48 antigen**	1.962	1.517	2.538	0.131	2.85E-07	7.25E-06
**Beta-klotho**	0.565	0.454	0.703	0.111	2.92E-07	7.38E-06
**Thrombospondin-2**	1.406	1.234	1.603	0.0667	3.11E-07	7.83E-06
**Inactive gamma-glutamyltranspeptidase 2**	0.696	0.605	0.799	0.071	3.14E-07	7.84E-06
**Proteasome subunit alpha type-1**	0.375	0.258	0.547	0.192	3.17E-07	7.84E-06
**Hedgehog-interacting protein**	0.359	0.243	0.532	0.2	3.16E-07	7.84E-06

**Baculoviral IAP repeat-containing protein 5**	0.282	0.174	0.458	0.248	3.20E-07	7.90E-06
**Interleukin-36 receptor antagonist protein**	0.608	0.502	0.736	0.0974	3.30E-07	8.10E-06
**Noelin**	1.585	1.328	1.893	0.0905	3.51E-07	8.53E-06
**Complement component C7**	3.126	2.016	4.848	0.224	3.51E-07	8.53E-06
**Adapter molecule crk**	1.43	1.245	1.642	0.0706	4.04E-07	9.76E-06
**Heat shock protein beta-6**	1.731	1.4	2.14	0.108	4.07E-07	9.80E-06
**Neuroendocrine protein 7B2**	0.614	0.509	0.742	0.0962	4.12E-07	9.86E-06
**Discoidin domain-containing receptor 2**	1.619	1.342	1.952	0.0957	4.78E-07	1.14E-05
**Ficolin-3**	0.575	0.463	0.713	0.11	4.84E-07	1.14E-05
**PTB domain-containing engulfment adapter protein 1**	0.449	0.329	0.613	0.159	4.80E-07	1.14E-05
**Oncostatin-M**	0.568	0.456	0.708	0.112	4.82E-07	1.14E-05
**Kinesin-like protein KIF16B**	0.311	0.198	0.491	0.232	5.06E-07	1.18E-05
**Frizzled-10**	0.42	0.299	0.589	0.173	5.18E-07	1.21E-05
**Phosphatidylinositol transfer protein beta isoform**	0.345	0.228	0.523	0.212	5.29E-07	1.22E-05
**Coagulation factor Xa**	0.581	0.47	0.718	0.108	5.40E-07	1.24E-05
**Microfibrillar-associated protein 2**	1.806	1.433	2.275	0.118	5.49E-07	1.26E-05
**Radiation-inducible immediate-early gene IEX-1**	0.352	0.234	0.53	0.209	5.54E-07	1.27E-05
**Ras-related and estrogen-regulated growth inhibitor**	0.27	0.162	0.451	0.262	5.68E-07	1.28E-05
**U6 snRNA-associated Sm-like protein LSm4**	0.379	0.259	0.554	0.194	5.66E-07	1.28E-05
**Semaphorin-6B**	2.099	1.57	2.807	0.148	5.65E-07	1.28E-05
**Cartilage intermediate layer protein 2**	0.555	0.441	0.699	0.118	5.85E-07	1.31E-05
**Protein MENT**	0.546	0.43	0.692	0.121	5.87E-07	1.31E-05
**SPARC-related modular calcium-binding protein 2**	2.223	1.624	3.043	0.16	6.06E-07	1.35E-05
**Troponin I, fast skeletal muscle**	1.382	1.217	1.57	0.065	6.23E-07	1.38E-05
**Butyrophilin subfamily 3 member A3**	1.701	1.38	2.096	0.107	6.37E-07	1.40E-05
**Hematopoietic prostaglandin D synthase**	0.542	0.425	0.689	0.123	6.39E-07	1.40E-05
**Sialic acid-binding Ig-like lectin 12**	0.447	0.325	0.613	0.162	6.49E-07	1.41E-05
**Tenascin**	1.954	1.501	2.544	0.135	6.50E-07	1.41E-05
**Kallistatin**	0.278	0.168	0.46	0.257	6.52E-07	1.41E-05
**Ubiquitin D**	0.276	0.166	0.458	0.259	6.61E-07	1.43E-05
**Beta-defensin 132**	0.557	0.443	0.702	0.118	6.67E-07	1.43E-05
**Heparan-sulfate 6-O-sulfotransferase 2**	1.683	1.37	2.067	0.105	6.91E-07	1.48E-05
**G-protein coupled receptor 26**	0.361	0.242	0.54	0.205	7.04E-07	1.49E-05
**Melanoma-associated antigen 5**	0.614	0.507	0.745	0.0982	7.05E-07	1.49E-05

**Insulin-like growth factor-binding protein 7**	2.3 73	1.685	3.342	0.175	7.48E- 07	1.58E-05
**Insulin growth factor-like family member 3**	0.4 97	0.377	0.656	0.141	7.55E- 07	1.59E-05
**IQ domain-containing protein F1**	0.3 6	0.24	0.54	0.207	7.74E- 07	1.62E-05
**Growth/differentiation factor 11/8**	0.4 62	0.34	0.628	0.157	8.18E- 07	1.70E-05
**Oxidized Protein deglycase DJ-1**	0.4 62	0.339	0.628	0.157	8.35E- 07	1.73E-05
**Alpha-parvin**	1.6 28	1.341	1.976	0.0989	8.53E- 07	1.76E-05
**Galanin**	0.2 68	0.158	0.453	0.269	9.44E- 07	1.94E-05
**Very low-density lipoprotein receptor**	0.4 34	0.311	0.606	0.17	9.64E- 07	1.97E-05
**Kininogen, HMW, Two Chain**	0.6 31	0.524	0.759	0.0943	1.02E- 06	2.07E-05
**Urotensin-2**	0.4 44	0.32	0.614	0.166	1.02E- 06	2.07E-05
**Ceruloplasmin**	0.4 64	0.341	0.631	0.157	1.02E- 06	2.07E-05
**Microfibril-associated glycoprotein 4**	1.5 35	1.293	1.824	0.0878	1.04E- 06	2.09E-05
**Colipase-like protein 2**	0.3 66	0.244	0.548	0.206	1.07E- 06	2.15E-05
**Kynurenine--oxoglutarate transaminase 1**	0.2 94	0.18	0.481	0.251	1.09E- 06	2.18E-05
**Dual specificity mitogen-activated protein kinase kinase 1**	1.5 96	1.322	1.927	0.0961	1.13E- 06	2.25E-05
**Mucin-16**	1.4 26	1.236	1.646	0.073	1.16E- 06	2.30E-05
**Neuromedin-B**	1.8 12	1.425	2.304	0.122	1.21E- 06	2.40E-05
**T-lymphocyte surface antigen Ly-9**	1.7 54	1.398	2.201	0.116	1.23E- 06	2.41E-05
**BPI fold-containing family A member 2**	0.6 08	0.497	0.743	0.103	1.26E- 06	2.46E-05
**Peregrin**	0.2 85	0.172	0.474	0.259	1.30E- 06	2.54E-05
**Fibroblast growth factor 23**	1.4 42	1.243	1.673	0.0757	1.33E- 06	2.58E-05
**Prostaglandin-H2 D-isomerase**	2.0 05	1.512	2.66	0.144	1.37E- 06	2.65E-05
**Hepatitis A virus cellular receptor 1**	1.2 97	1.167	1.442	0.0539	1.37E- 06	2.65E-05
**C1GALT1-specific chaperone 1**	0.4 92	0.369	0.656	0.147	1.38E- 06	2.66E-05
**Neurexophilin-2**	0.6 93	0.597	0.804	0.0762	1.42E- 06	2.73E-05
**Ribonuclease K6**	1.5 79	1.311	1.901	0.0948	1.46E- 06	2.79E-05
**Atrial natriuretic factor**	1.7 12	1.375	2.131	0.112	1.51E- 06	2.86E-05
**R-spondin-1**	1.6 26	1.334	1.982	0.101	1.52E- 06	2.87E-05
**BPI fold-containing family A member 1**	0.3 45	0.223	0.533	0.222	1.66E- 06	3.12E-05
**2'-deoxynucleoside 5'-phosphate N-hydrolase 1**	0.3 55	0.233	0.543	0.216	1.66E- 06	3.12E-05
**Collagen alpha-1(XV) chain**	2.4 76	1.708	3.588	0.189	1.69E- 06	3.15E-05
**Nuclear pore complex-interacting protein family member B3**	0.3 98	0.273	0.58	0.192	1.69E- 06	3.15E-05
**Early endosome antigen 1**	0.3 81	0.257	0.566	0.202	1.70E- 06	3.15E-05
**Neuronal pentraxin-2**	0.4 36	0.31	0.613	0.174	1.81E- 06	3.36E-05

**Secreted frizzled-related protein 2**	0.321	0.201	0.512	0.238	1.88E-06	3.47E-05
**Cyclic AMP-responsive element-binding protein 3-like protein 4**	1.982	1.494	2.63	0.144	2.12E-06	3.89E-05
**Tartrate-resistant acid phosphatase type 5**	0.47	0.344	0.642	0.16	2.18E-06	3.99E-05
**Rap guanine nucleotide exchange factor 5**	0.49	0.364	0.658	0.151	2.26E-06	4.11E-05
**Cathepsin H**	1.616	1.324	1.972	0.102	2.26E-06	4.11E-05
**Hephaestin-like protein 1**	0.261	0.15	0.456	0.284	2.27E-06	4.12E-05
**Leukocyte cell-derived chemotaxin-2**	0.668	0.565	0.79	0.0855	2.39E-06	4.31E-05
**Troponin T, fast skeletal muscle**	0.456	0.329	0.632	0.167	2.49E-06	4.47E-05
**Bone morphogenetic protein 7**	1.791	1.405	2.284	0.124	2.56E-06	4.58E-05
**Bone morphogenetic protein 4**	1.698	1.362	2.118	0.113	2.61E-06	4.65E-05
**Ectonucleotide pyrophosphatase/phosphodiesterase family member 5**	0.627	0.516	0.762	0.0996	2.83E-06	5.04E-05
**Keratin-associated protein 2-4**	1.694	1.359	2.113	0.113	2.88E-06	5.09E-05
**Transmembrane protein 185A**	0.423	0.295	0.606	0.184	2.88E-06	5.09E-05
**Brorin**	1.855	1.432	2.403	0.132	2.91E-06	5.12E-05
**Mannosyl-oligosaccharide 1,2-alpha-mannosidase IB**	0.445	0.316	0.625	0.174	3.10E-06	5.44E-05
**Coagulation Factor X**	0.59	0.473	0.737	0.113	3.18E-06	5.56E-05
**Cytosolic non-specific dipeptidase**	0.466	0.338	0.643	0.164	3.24E-06	5.64E-05
**Oral cancer-overexpressed protein 1**	0.675	0.571	0.797	0.0849	3.55E-06	6.16E-05
**Insulin-like growth factor-binding protein 6**	2.95	1.867	4.661	0.233	3.58E-06	6.18E-05
**Forkhead box protein J2**	1.314	1.171	1.475	0.0589	3.59E-06	6.19E-05
**Mast cell-expressed membrane protein 1**	0.421	0.292	0.607	0.187	3.66E-06	6.28E-05
**BMP and activin membrane-bound inhibitor homolog**	1.569	1.296	1.9	0.0975	3.77E-06	6.43E-05
**A-kinase anchor protein 7 isoforms alpha and beta**	0.351	0.225	0.547	0.226	3.77E-06	6.43E-05
**Beta-crystallin B1**	1.405	1.216	1.622	0.0735	3.81E-06	6.48E-05
**Retinoblastoma-associated protein**	0.333	0.209	0.531	0.238	3.86E-06	6.53E-05
**Trefoil factor 3**	1.701	1.357	2.131	0.115	3.94E-06	6.66E-05
**Zinc transporter 3**	0.568	0.446	0.722	0.123	4.08E-06	6.86E-05
**Secreted frizzled-related protein 1**	1.561	1.292	1.888	0.0968	4.17E-06	6.99E-05
**Activin receptor type-2A**	0.353	0.227	0.55	0.226	4.25E-06	7.11E-05
**Monocyte differentiation antigen CD14, soluble**	3.045	1.893	4.897	0.242	4.38E-06	7.29E-05
**Myotubularin-related protein 1**	0.356	0.229	0.554	0.225	4.54E-06	7.54E-05
**Peptidase inhibitor 15**	0.284	0.166	0.487	0.275	4.60E-06	7.61E-05
**Tenascin**	1.939	1.46	2.575	0.145	4.69E-06	7.74E-05
**Eukaryotic initiation factor 4A-I**	1.263	1.142	1.396	0.0512	5.05E-06	8.29E-05

**Scavenger receptor class A member 5**	1.569	1.293	1.904	0.0988	5.12E-06	8.38E-05
**Interleukin-7**	1.608	1.31	1.973	0.104	5.41E-06	8.84E-05
**ATP synthase subunit beta, mitochondrial**	0.542	0.416	0.706	0.135	5.62E-06	9.14E-05
**Transcription initiation factor TFIID subunit 10**	0.534	0.407	0.7	0.139	5.87E-06	9.51E-05
**Hexokinase-3**	0.395	0.264	0.591	0.205	5.95E-06	9.62E-05
**Complement factor H-related protein 5**	2.255	1.586	3.207	0.18	6.00E-06	9.66E-05
**Neutrophil gelatinase-associated lipocalin**	1.691	1.347	2.123	0.116	6.04E-06	9.68E-05
**Serine/threonine-protein kinase receptor R3**	1.429	1.224	1.668	0.0789	6.04E-06	9.68E-05
**Zymogen granule protein 16 homolog B**	1.383	1.202	1.592	0.0717	6.20E-06	9.90E-05
**Microtubule-associated proteins 1A/1B light chain 3A**	1.484	1.25	1.761	0.0873	6.28E-06	1.00E-04
**Triggering receptor expressed on myeloid cells 1**	1.712	1.355	2.163	0.119	6.53E-06	0.000103581
**T-cell antigen CD7**	1.37	1.195	1.571	0.0698	6.57E-06	0.000103773
**Beta-Ala-His dipeptidase**	0.782	0.703	0.87	0.0545	6.59E-06	0.000103904
**Aldo-keto reductase family 1 member B10**	0.721	0.625	0.831	0.0726	6.69E-06	0.00010502
**Carboxypeptidase N subunit 2**	0.428	0.296	0.62	0.188	6.86E-06	0.000107413
**Macrophage-stimulating protein receptor**	0.468	0.336	0.651	0.169	6.98E-06	0.000108212
**Cyclin-dependent kinase 5:Cyclin-dependent kinase 5 activator 1 complex**	0.596	0.476	0.747	0.115	6.97E-06	0.000108212
**Protein S100-A13**	1.404	1.211	1.628	0.0755	6.96E-06	0.000108212
**Pappalysin-1**	1.512	1.263	1.812	0.0921	7.06E-06	0.000109096
**Sorbitol dehydrogenase**	0.747	0.657	0.849	0.0651	7.41E-06	0.000113821
**Leucine-rich repeat and fibronectin type-III domain-containing protein 5**	0.536	0.408	0.704	0.139	7.41E-06	0.000113821
**Angiostatin**	0.352	0.223	0.556	0.233	7.56E-06	0.000115491
**Myosin regulatory light chain 2, atrial isoform**	0.559	0.433	0.721	0.13	7.58E-06	0.000115491
**Tyrosine-protein kinase ZAP-70**	0.689	0.585	0.811	0.0833	7.56E-06	0.000115491
**Retinal dehydrogenase 1**	0.735	0.642	0.841	0.0688	7.82E-06	0.000118726
**Zinc finger protein 276**	0.626	0.51	0.769	0.105	8.29E-06	0.000125446
**Plasma kallikrein**	0.356	0.226	0.56	0.232	8.42E-06	0.000127037
**ADP-dependent glucokinase**	2.172	1.543	3.057	0.175	8.84E-06	0.000133048
**tRNA-dihydrouridine(20) synthase [NAD(P)+]-like**	0.555	0.428	0.72	0.132	8.90E-06	0.000133072
**Vacuolar protein sorting-associated protein 28 homolog**	0.569	0.444	0.73	0.127	8.88E-06	0.000133072
**Protein CASC4**	1.667	1.33	2.09	0.115	9.26E-06	0.000138106
**Lysosomal acid phosphatase**	1.272	1.144	1.415	0.0544	9.41E-06	0.000139853
**Kallikrein-10**	1.588	1.294	1.949	0.104	9.59E-06	0.000142042
**Transmembrane emp24 domain-containing protein 10**	1.587	1.293	1.947	0.104	9.88E-06	0.000145992

**Signaling lymphocytic activation molecule**	1.482	1.245	1.764	0.089	1.00E-05	0.000147496
**Selenoprotein S**	0.564	0.437	0.728	0.13	1.05E-05	0.000154341
**TOM1-like protein 2**	0.524	0.393	0.698	0.147	1.05E-05	0.000154341
**Inter-alpha-trypsin inhibitor heavy chain H3**	1.791	1.382	2.321	0.132	1.06E-05	0.000154724
**Tumor necrosis factor receptor superfamily member 4**	1.793	1.383	2.326	0.133	1.07E-05	0.000155855
**Glutathione S-transferase A1**	0.627	0.509	0.772	0.106	1.08E-05	0.00015692
**Tropomyosin beta chain**	0.665	0.555	0.798	0.0926	1.09E-05	0.00015692
**Histone-lysine N-methyltransferase SUV420H2**	0.308	0.182	0.52	0.268	1.09E-05	0.00015692
**Dolichyl-diphosphooligosaccharide--protein glycosyltransferase 48 kDa subunit**	1.444	1.226	1.702	0.0836	1.10E-05	0.000157742
**Cadherin-11**	0.449	0.314	0.642	0.182	1.10E-05	0.000158242
**Oligodendrocyte-myelin glycoprotein**	0.705	0.603	0.824	0.0795	1.12E-05	0.00016055
**Netrin receptor UNC5B**	1.815	1.391	2.369	0.136	1.14E-05	0.000162342
**Arginine/serine-rich protein 1**	1.734	1.355	2.218	0.126	1.19E-05	0.000168835
**Ectonucleoside triphosphate diphosphohydrolase 3**	0.576	0.45	0.737	0.126	1.21E-05	0.000171717
**Secretoglobin family 3A member 1**	1.376	1.193	1.588	0.073	1.22E-05	0.000171977
**Polypeptide N-acetylgalactosaminyltransferase 1**	1.753	1.363	2.256	0.129	1.24E-05	0.000175271
**Protein zwilch homolog**	0.466	0.331	0.657	0.175	1.29E-05	0.000180261
**Bone morphogenetic protein 6**	1.63	1.309	2.03	0.112	1.28E-05	0.000180261
**Transmembrane protein C1orf162**	0.768	0.683	0.865	0.0604	1.29E-05	0.000180304
**Nuclear factor of activated T-cells, cytoplasmic 1**	0.405	0.27	0.608	0.207	1.30E-05	0.000180961
**Tenomodulin**	0.393	0.258	0.598	0.214	1.31E-05	0.000182001
**Receptor-type tyrosine-protein kinase FLT3**	0.556	0.427	0.725	0.135	1.35E-05	0.000187664
**Endothelial cell-selective adhesion molecule**	1.903	1.423	2.544	0.148	1.42E-05	0.000195427
**Sodium channel subunit beta-4**	0.485	0.35	0.673	0.167	1.41E-05	0.000195427
**Four and a half LIM domains protein 1**	0.468	0.332	0.659	0.175	1.45E-05	0.000200047
**Coagulation Factor XI**	0.441	0.305	0.639	0.189	1.46E-05	0.000200202
**Chromogranin-A**	1.665	1.322	2.098	0.118	1.49E-05	0.000203554
**Interleukin-1 receptor type 1**	1.625	1.304	2.026	0.113	1.59E-05	0.000216269
**Tenascin**	1.453	1.226	1.722	0.0866	1.59E-05	0.000216269
**UDP-glucuronic acid decarboxylase 1**	1.891	1.416	2.525	0.148	1.59E-05	0.000216269
**NSFL1 cofactor p47**	1.291	1.15	1.451	0.0593	1.63E-05	0.000221143
**Bombesin receptor-activated protein C6orf89**	1.721	1.344	2.204	0.126	1.66E-05	0.000223442
**Adenylate kinase 4, mitochondrial**	0.409	0.272	0.614	0.208	1.68E-05	0.000226042
**Fatty acid-binding protein 9**	0.547	0.415	0.72	0.141	1.73E-05	0.000232552

**Hemojuvelin**	0.352	0.218	0.567	0.244	1.77E-05	0.000237026
**Carbonic anhydrase-related protein 11**	1.534	1.261	1.865	0.0998	1.81E-05	0.000241275
**E3 ubiquitin-protein ligase RNF43**	0.449	0.311	0.648	0.187	1.88E-05	0.000250694
**CD5 antigen-like**	1.418	1.208	1.664	0.0817	1.92E-05	0.000254769
**Urokinase plasminogen activator surface receptor**	1.86	1.399	2.473	0.145	1.92E-05	0.000254769
**Probable E3 ubiquitin-protein ligase HERC4**	0.327	0.196	0.546	0.262	1.93E-05	0.000255045
**Cathepsin O**	1.617	1.297	2.016	0.113	1.95E-05	0.000256202
**Troponin I, cardiac muscle**	1.308	1.156	1.479	0.0628	1.96E-05	0.000257991
**Phosphate-regulating neutral endopeptidase**	1.598	1.288	1.982	0.11	2.00E-05	0.000262039
**Retinoic acid receptor responder protein 2**	2.248	1.548	3.264	0.19	2.07E-05	0.000270886
**NADH dehydrogenase [ubiquinone] 1 beta subcomplex subunit 11, mitochondrial**	0.399	0.261	0.609	0.216	2.09E-05	0.000272192
**Cathepsin F**	0.523	0.387	0.705	0.153	2.14E-05	0.000278287
**Dual specificity protein kinase CLK2**	0.611	0.486	0.767	0.116	2.17E-05	0.000280793
**Inhibin beta B chain**	1.532	1.258	1.865	0.1	2.19E-05	0.000283153
**DnaJ homolog subfamily C member 4**	0.506	0.369	0.694	0.161	2.32E-05	0.000299896
**Epididymal-specific lipocalin-10**	0.593	0.465	0.756	0.124	2.36E-05	0.000303463
**C-C motif chemokine 1**	0.612	0.487	0.768	0.116	2.40E-05	0.000307562
**Complement C1q tumor necrosis factor-related protein 3**	0.404	0.265	0.615	0.214	2.40E-05	0.000307562
**Dual specificity mitogen-activated protein kinase kinase 2**	1.537	1.259	1.876	0.102	2.46E-05	0.000313706
**Solute carrier family 35 member G2**	0.637	0.516	0.785	0.107	2.48E-05	0.000315964
**Galactosylceramide sulfotransferase**	0.639	0.519	0.787	0.106	2.55E-05	0.000324188
**Protein NDRG4**	0.443	0.304	0.648	0.193	2.58E-05	0.00032558
**Voltage-dependent calcium channel subunit alpha-2/delta-3**	0.537	0.402	0.717	0.148	2.57E-05	0.00032558
**Glycerol-3-phosphate dehydrogenase 1-like protein**	0.609	0.484	0.768	0.118	2.59E-05	0.000326843
**Interleukin-9**	1.635	1.3	2.057	0.117	2.61E-05	0.000327474
**Phospholipase A2, membrane associated**	1.457	1.223	1.737	0.0895	2.61E-05	0.000327474
**Proteasome subunit beta type-5**	0.361	0.224	0.58	0.243	2.66E-05	0.00033246
**DNA repair protein RAD51 homolog 1**	0.653	0.535	0.797	0.101	2.68E-05	0.000335044
**Cystatin-M**	1.675	1.316	2.131	0.123	2.77E-05	0.000343748
**C-type mannose receptor 2**	2.042	1.463	2.851	0.17	2.77E-05	0.000343748
**Cysteine-rich motor neuron 1 protein**	1.828	1.377	2.426	0.144	2.98E-05	0.000369522
**C-C motif chemokine 3-like 1**	0.46	0.319	0.662	0.186	3.01E-05	0.00037247
**Adenylyltransferase and sulfurtransferase MOCS3**	0.479	0.339	0.677	0.176	3.04E-05	0.000374568
**Netrin-G1**	1.501	1.24	1.817	0.0974	3.05E-05	0.000375341

**TOMM20-like protein 1**	0.544	0.408	0.725	0.147	3.29E-05	0.000403688
**Electron transfer flavoprotein subunit alpha, mitochondrial**	0.443	0.301	0.65	0.196	3.31E-05	0.000404802
**Macrophage-capping protein**	1.436	1.21	1.703	0.0871	3.32E-05	0.000405759
**Thioredoxin domain-containing protein 5**	1.611	1.286	2.019	0.115	3.43E-05	0.000417743
**Afamin**	0.511	0.372	0.702	0.162	3.50E-05	0.000424542
**Ribosomal protein S6 kinase beta-1**	0.528	0.39	0.715	0.154	3.50E-05	0.000424542
**Syntaxin-7**	1.303	1.149	1.477	0.064	3.56E-05	0.00043029
**Stress-70 protein, mitochondrial**	1.284	1.141	1.446	0.0605	3.58E-05	0.000431694
**NADH dehydrogenase [ubiquinone] 1 beta subcomplex subunit 4**	0.683	0.57	0.819	0.0922	3.63E-05	0.000436203
**Fumarylacetoacetase**	0.688	0.576	0.822	0.0906	3.69E-05	0.000442221
**Formimidoyltransferase-cyclodeaminase**	0.876	0.823	0.933	0.0321	3.83E-05	0.00045771
**Carbonic anhydrase 6**	0.754	0.659	0.862	0.0687	3.83E-05	0.00045771
**Alanine aminotransferase 1**	0.528	0.39	0.716	0.155	3.92E-05	0.000466905
**Regulator of G-protein signaling 19**	0.408	0.266	0.625	0.218	3.93E-05	0.000466905
**Proto-oncogene tyrosine-protein kinase receptor Ret**	0.621	0.495	0.78	0.116	4.17E-05	0.000494721
**Protein kinase C-binding protein NELL2**	1.976	1.426	2.737	0.166	4.28E-05	0.000506387
**Pre-mRNA-splicing factor ATP-dependent RNA helicase PRP16**	0.427	0.284	0.642	0.208	4.36E-05	0.00051402
**RNA polymerase II elongation factor ELL**	0.257	0.134	0.494	0.333	4.52E-05	0.000531784
**Sorcin**	1.367	1.176	1.589	0.0768	4.65E-05	0.000545705
**Small conductance calcium-activated potassium channel protein 1**	0.341	0.203	0.572	0.265	4.74E-05	0.000555256
**ADP-sugar pyrophosphatase**	1.642	1.293	2.086	0.122	4.82E-05	0.000561477
**Semaphorin-3G**	0.476	0.333	0.681	0.182	4.81E-05	0.000561477
**Tumor protein p53-inducible protein 11**	0.817	0.741	0.901	0.0499	4.88E-05	0.000566296
**E3 ubiquitin-protein ligase NEURL1**	0.311	0.177	0.547	0.287	4.88E-05	0.000566296
**Prolactin-releasing peptide**	0.408	0.264	0.63	0.222	5.16E-05	0.000597356
**Proliferation-associated protein 2G4**	1.291	1.141	1.461	0.0631	5.20E-05	0.000600004
**Anterior gradient protein 2 homolog**	0.246	0.125	0.485	0.346	5.21E-05	0.000600004
**Ephrin-A4**	1.663	1.299	2.128	0.126	5.39E-05	0.000619085
**BolA-like protein 1**	1.428	1.201	1.698	0.0883	5.40E-05	0.000619085
**Pigment epithelium-derived factor**	3.4	1.876	6.162	0.303	5.48E-05	0.000627288
**N-acylethanolamine-hydrolyzing acid amidase**	0.73	0.627	0.851	0.078	5.52E-05	0.000630688
**Cellular retinoic acid-binding protein 1**	0.44	0.295	0.656	0.204	5.58E-05	0.000635581
**Delta-like protein 1**	1.632	1.286	2.072	0.122	5.67E-05	0.000643499
**Olfactory marker protein**	0.454	0.309	0.667	0.196	5.67E-05	0.000643499

**Netrin receptor UNC5B**	1.721	1.321	2.242	0.135	5.77E-05	0.000652705
**Glutathione S-transferase P**	1.525	1.241	1.875	0.105	6.02E-05	0.000679902
**3'(2'),5'-bisphosphate nucleotidase 1**	1.28	1.134	1.444	0.0615	6.13E-05	0.000690566
**Coiled-coil domain-containing protein 80**	2.126	1.47	3.077	0.189	6.28E-05	0.000703854
**Galectin-8**	1.494	1.228	1.819	0.1	6.28E-05	0.000703854
**Uroplakin-3b-like protein**	0.584	0.449	0.76	0.134	6.39E-05	0.000714475
**Carboxypeptidase D**	0.304	0.17	0.545	0.298	6.47E-05	0.00072253
**Smoothelin**	1.202	1.098	1.315	0.046	6.50E-05	0.000723819
**Calcium-binding and coiled-coil domain-containing protein 2**	2.097	1.458	3.017	0.185	6.52E-05	0.000724224
**Beta-enolase**	0.739	0.637	0.858	0.0761	7.09E-05	0.000785297
**Protein S100-A12**	1.4	1.186	1.653	0.0847	7.10E-05	0.000785297
**Thioredoxin domain-containing protein 15**	1.511	1.232	1.852	0.104	7.22E-05	0.000797482
**Cytohesin-2**	0.236	0.116	0.482	0.364	7.29E-05	0.000803225
**CD209 antigen**	0.636	0.509	0.796	0.114	7.34E-05	0.00080706
**Cysteine and glycine-rich protein 3**	1.467	1.214	1.773	0.0967	7.43E-05	0.00081536
**Heparin cofactor 2**	0.484	0.338	0.693	0.183	7.47E-05	0.000817614
**Retinol-binding protein 4**	0.53	0.387	0.726	0.161	7.57E-05	0.000826484
**Leucine-rich repeat and fibronectin type III domain-containing protein 1**	0.413	0.266	0.641	0.224	7.95E-05	0.000865931
**Cell growth regulator with EF hand domain protein 1**	1.817	1.351	2.445	0.151	7.99E-05	0.000868575
**Tenascin**	1.607	1.269	2.035	0.12	8.15E-05	0.000884555
**Roundabout homolog 2**	2.385	1.547	3.675	0.221	8.22E-05	0.000889197
**Vesicular integral-membrane protein VIP36**	0.611	0.478	0.781	0.125	8.35E-05	0.000902349
**Fatty acid-binding protein, liver**	0.597	0.462	0.772	0.131	8.40E-05	0.000905232
**Interleukin-23**	1.458	1.208	1.76	0.0961	8.77E-05	0.000943184
**Endostatin**	1.979	1.407	2.784	0.174	8.85E-05	0.00094594
**Interleukin-15 receptor subunit alpha**	1.597	1.264	2.018	0.119	8.84E-05	0.00094594
**Fc receptor-like protein 5**	1.379	1.174	1.619	0.0819	8.83E-05	0.00094594
**Vascular endothelial growth factor D**	1.378	1.173	1.617	0.0819	9.13E-05	0.000973068
**L-xylulose reductase**	0.759	0.661	0.871	0.0706	9.19E-05	0.000977367
**Peptidyl-prolyl cis-trans isomerase-like 2**	0.582	0.443	0.763	0.139	9.23E-05	0.000979852
**Small ubiquitin-related modifier 2**	1.43	1.195	1.711	0.0915	9.33E-05	0.000988073
**CCAAT/enhancer-binding protein beta**	0.642	0.514	0.802	0.113	9.42E-05	0.000996243
**Rab9 effector protein with kelch motifs**	0.402	0.254	0.636	0.234	9.71E-05	0.001023827
**Superoxide dismutase [Mn], mitochondrial**	0.52	0.374	0.723	0.168	9.78E-05	0.001027781

**Protein sel-1 homolog 2**	0.455	0.307	0.676	0.202	9.77E-05	0.001027781
**Hsc70-interacting protein**	1.533	1.236	1.9	0.11	9.91E-05	0.001038468
**Phosphatidate phosphatase PPAPDC1A**	0.508	0.361	0.714	0.174	0.000101303	0.001059635
**Ribonuclease 4**	2.4	1.543	3.734	0.225	0.000102107	0.001060874
**Adenosine deaminase CECR1**	1.462	1.207	1.77	0.0977	0.000101758	0.001060874
**RecQ-mediated genome instability protein 1**	0.384	0.237	0.623	0.246	0.000102273	0.001060874
**Protein phosphatase 1 regulatory subunit 3B**	0.741	0.637	0.862	0.0772	0.000102054	0.001060874
**Interferon alpha-5**	0.412	0.263	0.644	0.229	0.000103446	0.001070807
**Gamma-aminobutyric acid receptor-associated protein-like 1**	1.625	1.272	2.076	0.125	0.000104164	0.001073774
**C-X-C motif chemokine 13**	1.425	1.192	1.704	0.0913	0.000104075	0.001073774
**PH and SEC7 domain-containing protein 2**	0.709	0.596	0.844	0.0888	0.000105831	0.001088706
**Elafin**	1.416	1.187	1.688	0.0897	0.000106787	0.001096273
**Collagen alpha-2(VI) chain**	1.539	1.237	1.915	0.111	0.000107609	0.00110244
**RNA-binding protein 24**	0.536	0.39	0.735	0.161	0.000109024	0.001114646
**Serine/threonine-protein kinase PAK 3**	0.549	0.405	0.744	0.155	0.000109548	0.0011177
**Acylphosphatase-2**	1.368	1.167	1.603	0.081	0.000109951	0.001119524
**Serine/threonine-protein kinase 17B**	0.534	0.389	0.734	0.162	0.000112706	0.001145228
**Calcium/calmodulin-dependent protein kinase type 1D**	0.529	0.383	0.731	0.165	0.000114599	0.001162097
**ProSAAS**	1.413	1.185	1.684	0.0896	0.000115604	0.0011624
**Protein S100-A11**	1.601	1.26	2.035	0.122	0.000115796	0.0011624
**Eukaryotic translation initiation factor 4H**	1.15	1.071	1.234	0.0362	0.000115553	0.0011624
**Limbic system-associated membrane protein**	1.982	1.4	2.806	0.177	0.000115075	0.0011624
**Hyaluronidase-1**	0.502	0.354	0.713	0.179	0.000115546	0.0011624
**Killer cell immunoglobulin-like receptor 2DL1**	0.46	0.31	0.683	0.202	0.000116589	0.001168004
**Serum amyloid A-2 protein**	1.215	1.1	1.342	0.0506	0.000117559	0.001175353
**Prefoldin subunit 5**	1.639	1.274	2.107	0.128	0.000118454	0.001181931
**Coagulation Factor V**	0.692	0.574	0.835	0.0956	0.000119334	0.001188328
**ADP-ribosylation factor 1**	1.294	1.135	1.476	0.0671	0.000120941	0.00119715
**G2/mitotic-specific cyclin-B2**	0.441	0.29	0.669	0.213	0.000120534	0.00119715
**Laminin-2**	2.129	1.448	3.129	0.197	0.00012071	0.00119715
**Peptidyl-prolyl cis-trans isomerase C**	1.961	1.391	2.765	0.175	0.00012185	0.001201373
**Membrane-associated progesterone receptor component 2**	1.351	1.159	1.574	0.0782	0.00012171	0.001201373
**Endothelial monocyte-activating polypeptide 2**	0.465	0.314	0.687	0.2	0.000123824	0.001218418
**Bone morphogenetic protein 3**	0.501	0.352	0.713	0.18	0.000124079	0.001218515

**Septin-6**	1.487	1.214	1.821	0.104	0.000126497	0.001239817
**Protein JTB**	1.813	1.337	2.458	0.155	0.0001272 5	0.001244748
**Spondin-2**	1.534	1.232	1.911	0.112	0.0001302	0.001271112
**NAD-dependent protein deacylase sirtuin-5, mitochondrial**	1.489	1.214	1.826	0.104	0.000130671	0.001273209
**Ephrin type-B receptor 4**	1.7	1.295	2.231	0.139	0.000132067	0.001284301
**mRNA-decapping enzyme 1B**	1.218	1.101	1.348	0.0517	0.0001324	0.001285025
**Neurogenic locus notch homolog protein 3**	2.267	1.49	3.451	0.214	0.000133287	0.001291122
**Collectin-10**	0.368	0.22	0.615	0.262	0.0001344	0.001299376
**Semenogelin-1**	1.857	1.351	2.552	0.162	0.000135576	0.001308208
**Peptidyl-prolyl cis-trans isomerase G**	0.666	0.541	0.821	0.106	0.000135921	0.001308995
**Cofilin-2**	1.181	1.084	1.287	0.0437	0.000137551	0.001322132
**Diablo homolog, mitochondrial**	1.457	1.2	1.767	0.0987	0.000137863	0.001322577
**Vascular endothelial growth factor receptor 2**	0.46	0.309	0.686	0.204	0.000138554	0.001326651
**Receptor-type tyrosine-protein phosphatase delta**	0.6	0.461	0.78	0.134	0.000140019	0.00133811
**Potassium voltage-gated channel subfamily G member 4**	0.445	0.293	0.676	0.213	0.000147547	0.001404654
**Dynein intermediate chain 1, axonemal**	0.518	0.368	0.727	0.173	0.000147498	0.001404654
**Gamma-aminobutyric acid receptor-associated protein**	1.555	1.238	1.954	0.116	0.000149732	0.001421969
**C-type lectin domain family 2 member A**	1.663	1.279	2.164	0.134	0.000149936	0.001421969
**Stromal interaction molecule 1**	0.517	0.368	0.727	0.174	0.000150437	0.001424006
**Rho guanine nucleotide exchange factor 7**	0.498	0.348	0.714	0.184	0.000150958	0.001426225
**Laminin subunit alpha-4**	0.425	0.273	0.662	0.226	0.000152248	0.001435684
**Interferon alpha/beta receptor 1**	1.526	1.226	1.9	0.112	0.000153421	0.001444015
**Procollagen C-endopeptidase enhancer 2**	0.547	0.4	0.748	0.159	0.000157205	0.001476839
**Growth/differentiation factor 8**	0.48	0.328	0.703	0.194	0.000158715	0.001488215
**Beta-microseminoprotein**	1.265	1.12	1.429	0.0623	0.000160496	0.001502086
**Glycolipid transfer protein domain-containing protein 2**	0.547	0.4	0.749	0.16	0.000161367	0.001507404
**Leucine-rich repeat-containing protein 25**	0.549	0.402	0.75	0.159	0.0001617 3	0.001507964
**Semaphorin-4B**	1.665	1.277	2.17	0.135	0.000163618	0.00152272
**Kallikrein-12**	0.697	0.578	0.841	0.0957	0.000166576	0.001547356
**Chromobox protein homolog 5**	0.499	0.348	0.717	0.185	0.000168104	0.001555741
**Serine/threonine-protein kinase 16**	0.502	0.35	0.718	0.183	0.000167831	0.001555741
**Sperm surface protein Sp17**	1.531	1.226	1.911	0.113	0.000171077	0.001580317
**Tumor necrosis factor receptor superfamily member 19L**	0.491	0.339	0.712	0.189	0.000172772	0.001593022
**Rho guanine nucleotide exchange factor 10**	0.53	0.381	0.739	0.169	0.000179241	0.001649614

**Cytochrome c oxidase assembly factor 3 homolog, mitochondrial**	0.437	0.284	0.674	0.221	0.000180056	0.00165406
**Carboxypeptidase M**	0.526	0.376	0.736	0.172	0.000181559	0.001664796
**mRNA-decapping enzyme 1A**	1.606	1.253	2.058	0.127	0.000182351	0.001668983
**Lymphotoxin alpha1:beta2**	1.601	1.251	2.048	0.126	0.000183345	0.001675002
**1-phosphatidylinositol 4,5-bisphosphate phosphodiesterase gamma-1**	0.419	0.266	0.661	0.232	0.000184371	0.001681286
**Oncoprotein-induced transcript 3 protein**	1.488	1.208	1.834	0.107	0.000188479	0.001715606
**Chitinase-3-like protein 1**	1.165	1.075	1.263	0.041	0.000190548	0.001731271
**Apolipoprotein A-V**	0.754	0.65	0.875	0.0758	0.000193502	0.001754908
**DNA-directed RNA polymerases I and III subunit RPAC1**	0.62	0.482	0.797	0.128	0.00019546	0.001769447
**Sialic acid-binding Ig-like lectin 8**	0.428	0.274	0.669	0.228	0.000196885	0.001779112
**Protocadherin beta-2**	0.511	0.359	0.728	0.18	0.000199505	0.001799519
**C-type lectin domain family 4 member D**	0.396	0.243	0.646	0.25	0.000206144	0.001856044
**Trafficking protein particle complex subunit 3**	1.563	1.234	1.978	0.12	0.000207141	0.001858295
**RELT-like protein 2**	0.571	0.424	0.768	0.151	0.000206811	0.001858295
**Cellular retinoic acid-binding protein 2**	1.504	1.212	1.866	0.11	0.000209018	0.001871763
**T-cell surface glycoprotein CD8 beta chain**	0.554	0.406	0.757	0.159	0.00021066	0.001883083
**Fermitin family homolog 3**	1.232	1.103	1.375	0.0563	0.000211527	0.001886129
**Prostaglandin E synthase 3**	0.619	0.48	0.798	0.13	0.000211759	0.001886129
**Transcription factor AP-1**	0.503	0.349	0.723	0.186	0.000214053	0.001903158
**Golgi-associated plant pathogenesis-related protein 1**	0.634	0.498	0.807	0.123	0.00021742	0.001929655
**Lumican**	1.992	1.382	2.872	0.187	0.000218931	0.001939601
**Opticin**	0.45	0.295	0.688	0.216	0.000220537	0.001950363
**Xyloside xylosyltransferase 1**	1.884	1.346	2.637	0.172	0.000221389	0.001954428
**Syntaxin-8**	1.279	1.122	1.457	0.0666	0.00022654	0.001996358
**Properdin**	0.491	0.337	0.717	0.193	0.000227685	0.002002907
**Tumor necrosis factor receptor superfamily member 3**	0.669	0.54	0.829	0.109	0.000228835	0.002009467
**ATP synthase subunit f, mitochondrial**	0.511	0.357	0.73	0.182	0.000229907	0.002015331
**Sulfotransferase family cytosolic 2B member 1**	0.49	0.335	0.716	0.194	0.00023388	0.002046557
**Sperm-associated antigen 11A**	0.541	0.389	0.75	0.167	0.000236193	0.002063171
**Low-density lipoprotein receptor-related protein 10**	1.699	1.281	2.253	0.144	0.000236761	0.00206451
**WNT1-inducible-signaling pathway protein 2**	1.742	1.296	2.342	0.151	0.000237487	0.002067216
**ATP synthase-coupling factor 6, mitochondrial**	1.201	1.089	1.324	0.0499	0.000239887	0.002081186
**Netrin-4**	1.763	1.303	2.385	0.154	0.000239928	0.002081186
**Tumor necrosis factor receptor superfamily member 1B**	1.536	1.221	1.932	0.117	0.000241082	0.002082137

High affinity immunoglobulin epsilon receptor subunit alpha	0.658	0.526	0.823	0.114	0.000241292	0.002082137
Cold shock domain-containing protein C2	0.518	0.364	0.736	0.179	0.000240682	0.002082137
Receptor-type tyrosine-protein phosphatase eta	0.354	0.203	0.616	0.283	0.000242044	0.002085011
Aflatoxin B1 aldehyde reductase member 3	0.813	0.728	0.908	0.0564	0.000244151	0.002099531
Twinfilin-1	1.279	1.121	1.459	0.0671	0.000245466	0.002107195
Protocadherin gamma-A1	1.176	1.078	1.282	0.0442	0.000248614	0.002126887
Cullin-4B	1.403	1.171	1.682	0.0924	0.000248525	0.002126887
Carboxypeptidase Q	0.664	0.533	0.827	0.112	0.000254311	0.002171895
SPARC-like protein 1	0.506	0.351	0.729	0.186	0.000255784	0.002180736
Ubiquitin-fold modifier-conjugating enzyme 1	1.287	1.124	1.473	0.0689	0.000256759	0.002185306
Brain-specific serine protease 4	1.673	1.269	2.205	0.141	0.000259039	0.002200946
N-acetyllactosaminide beta-1,3-N-acetylglucosaminyltransferase 2	0.396	0.24	0.651	0.254	0.000259693	0.002202747
Beta-defensin 128	0.438	0.282	0.683	0.226	0.000262345	0.002221458
Mast/stem cell growth factor receptor Kit	0.613	0.472	0.798	0.134	0.000263159	0.002222788
Activin A	1.44	1.184	1.752	0.1	0.000263395	0.002222788
Membrane-associated progesterone receptor component 2	0.533	0.38	0.748	0.173	0.000270684	0.002273414
Cadherin-related family member 5	0.436	0.278	0.681	0.228	0.000270764	0.002273414
Intracellular hyaluronan-binding protein 4	1.5	1.206	1.865	0.111	0.000270223	0.002273414
Protein SET	0.414	0.257	0.666	0.243	0.000283788	0.002374756
Protein p13 MTCP-1	0.601	0.456	0.791	0.14	0.000283347	0.002374756
Tyrosine-protein kinase ZAP-70	0.578	0.43	0.777	0.151	0.000285487	0.002384968
Procollagen-lysine,2-oxoglutarate 5-dioxygenase 2	1.394	1.165	1.668	0.0916	0.000286192	0.00238685
GSK3-beta interaction protein	1.392	1.164	1.664	0.0912	0.000287489	0.002393658
Alpha-N-acetylglucosaminidase	1.379	1.159	1.64	0.0886	0.000289055	0.002398679
Neurocan core protein	0.683	0.555	0.839	0.105	0.000289009	0.002398679
Phosphoglycerate mutase 1	1.094	1.042	1.148	0.0247	0.000292826	0.002425925
Dolichyl-diphosphooligosaccharide--protein glycosyltransferase subunit 1	1.34	1.144	1.571	0.0809	0.000296664	0.002453634
Peptidyl-prolyl cis-trans isomerase FKBP7	1.373	1.156	1.63	0.0876	0.000298317	0.002463216
Glucoside xylosyltransferase 1	0.566	0.416	0.771	0.157	0.000299796	0.002471329
Vascular endothelial growth factor A	1.916	1.346	2.726	0.18	0.000305475	0.002513986
Syntaxin-4	1.221	1.095	1.36	0.0553	0.00031193	0.002562868
Neuronal growth regulator 1	0.415	0.258	0.67	0.244	0.000316935	0.002599699
Cystatin-D	1.289	1.123	1.48	0.0706	0.000320418	0.002621841
Platelet-derived growth factor receptor alpha	1.331	1.139	1.556	0.0795	0.000320687	0.002621841

**Tachykinin-4**	0.3 98	0.241	0.658	0.256	0.0003 29641	0.00269062 5
**Coagulation factor VII**	0.6 72	0.541	0.835	0.111	0.0003 3358	0.00271831 9
**Protocadherin alpha-C2**	1.4 54	1.185	1.785	0.105	0.0003 40437	0.00276966 9
**Prosaposin**	1.5 91	1.234	2.052	0.13	0.0003 41226	0.00277155 5
**Prolyl 3-hydroxylase 1**	0.4 14	0.256	0.671	0.246	0.0003 44639	0.00279471 5
**Vasodilator-stimulated phosphoprotein**	1.1 71	1.074	1.276	0.0441	0.0003 45589	0.00279787
**Astrocytic phosphoprotein PEA-15**	1.3 84	1.158	1.655	0.091	0.0003 51214	0.00283879
**Insulin-like growth factor-binding protein complex acid labile subunit**	0.7 15	0.595	0.86	0.094	0.0003 58545	0.00287935 1
**Ephrin-A2**	1.6 85	1.265	2.244	0.146	0.0003 5843	0.00287935 1
**Histone-lysine N-methyltransferase 2D**	0.3 95	0.238	0.658	0.26	0.0003 5705	0.00287935 1
**Serpin A9**	1.5 39	1.215	1.95	0.121	0.0003 57646	0.00287935 1
**Kallikrein-6**	0.6 54	0.518	0.826	0.119	0.0003 60689	0.00289189 7
**NGFI-A-binding protein 1**	0.6 79	0.548	0.84	0.109	0.0003 62441	0.00290127 5
**Thyroglobulin**	1.2 01	1.086	1.329	0.0515	0.0003 66043	0.00292541
**Cell adhesion molecule 3**	1.4 28	1.174	1.737	0.1	0.0003 70496	0.00295151 7
**Interleukin-34**	0.6 48	0.51	0.823	0.122	0.0003 70383	0.00295151 7
**Aurora kinase B**	0.5 12	0.354	0.74	0.188	0.0003 71365	0.00295371 6
**cAMP-dependent protein kinase catalytic subunit alpha**	1.1 54	1.066	1.249	0.0403	0.0003 74347	0.00297203 4
**Augurin**	0.6 04	0.457	0.797	0.142	0.0003 74862	0.00297203 4
**T-cell immunoreceptor with Ig and ITIM domains**	0.6 47	0.509	0.823	0.122	0.0003 76718	0.00298199 8
**Amyloid-like protein 1**	0.7 24	0.606	0.865	0.0908	0.0003 79044	0.00299565 2
**Gamma-enolase**	1.3 1	1.129	1.521	0.0761	0.0003 8043	0.00300183 8
**Thrombin**	0.6 89	0.561	0.847	0.105	0.0003 91745	0.00308136
**Cyclic AMP-dependent transcription factor ATF-6 beta**	0.5 99	0.452	0.795	0.144	0.0003 91576	0.00308136
**Hepatocyte growth factor**	1.3 53	1.144	1.599	0.0854	0.0003 99907	0.00314059 5
**Ribonuclease P protein subunit p30**	0.5 06	0.347	0.738	0.193	0.0004 08397	0.00320221 6
**Ribonuclease T2**	1.6 27	1.242	2.132	0.138	0.0004 11017	0.00321769 3
**Protein tyrosine phosphatase type IVA 2**	1.3 15	1.13	1.531	0.0775	0.0004 11734	0.00321824 5
**Phosphatidylinositol 4-phosphate 3-kinase C2 domain-containing subunit alpha**	0.4 78	0.317	0.72	0.209	0.0004 12968	0.00322283
**Osteopetrosis-associated transmembrane protein 1**	1.8 32	1.309	2.565	0.172	0.0004 16471	0.00324508 6
**Dual specificity protein phosphatase 26**	0.6 57	0.52	0.83	0.119	0.0004 36073	0.00338721 6
**Inositol polyphosphate 5-phosphatase OCRL-1**	0.5 4	0.383	0.761	0.175	0.0004 35539	0.00338721 6
**Ephrin-B1**	1.5 7	1.221	2.019	0.128	0.0004 42761	0.00342847 1
**Hepatocyte nuclear factor 4-alpha**	1.8 33	1.307	2.57	0.172	0.0004 42208	0.00342847 1

**WNT1-inducible-signaling pathway protein 1**	0.603	0.455	0.8	0.144	0.000444657	0.003437807
**Vitamin D3 receptor**	0.48	0.318	0.723	0.209	0.000445813	0.003441397
**RUN and FYVE domain-containing protein 1**	0.526	0.368	0.753	0.183	0.000447562	0.003449553
**Methyltransferase-like protein 24**	1.367	1.148	1.628	0.0891	0.000450749	0.003468749
**Tuftelin-interacting protein 11**	0.624	0.48	0.812	0.134	0.000455871	0.003502753
**Importin subunit alpha-1**	1.45	1.178	1.784	0.106	0.000461251	0.003538624
**COP9 signalosome complex subunit 7b**	0.832	0.75	0.922	0.0526	0.000466121	0.003570486
**VIP36-like protein**	1.213	1.089	1.351	0.0552	0.000469628	0.003591821
**Phospholipid transfer protein**	1.839	1.307	2.588	0.174	0.000471216	0.00359844
**Arf-GAP with GTPase, ANK repeat and PH domain-containing protein 3**	0.643	0.501	0.824	0.127	0.00047862	0.003649384
**Phosphoserine aminotransferase**	0.794	0.697	0.904	0.0663	0.000488062	0.003715692
**Protein-tyrosine sulfotransferase 2**	0.431	0.268	0.692	0.242	0.000491335	0.003734896
**beta-nerve growth factor**	1.264	1.108	1.443	0.0673	0.000493566	0.003746138
**Urea transporter 2**	1.499	1.193	1.883	0.116	0.000513804	0.003893807
**Sorting nexin-17**	0.624	0.478	0.815	0.136	0.000529853	0.00400933
**Neuroendocrine convertase 2**	1.45	1.175	1.79	0.107	0.000532242	0.004021292
**Carnitine O-palmitoyltransferase 1, muscle isoform**	0.53	0.37	0.759	0.184	0.000533401	0.004023946
**Phosphoglycerate mutase 2**	1.201	1.083	1.332	0.0529	0.000536431	0.004040681
**Caveolin-3**	1.603	1.227	2.094	0.136	0.000540648	0.00406629
**Apolipoprotein L1**	0.587	0.434	0.794	0.154	0.00054791	0.004114695
**Desmocollin-2**	1.672	1.249	2.238	0.149	0.000556981	0.00417652
**Importin subunit beta-1**	1.167	1.069	1.274	0.0448	0.000564109	0.004223605
**Interleukin-6**	1.299	1.119	1.507	0.0758	0.000567452	0.004242254
**Histone deacetylase 8**	0.563	0.406	0.781	0.167	0.000577033	0.004307414
**Tax1-binding protein 3**	1.322	1.128	1.551	0.0812	0.000577905	0.004307471
**Phosphatidylinositol 4,5-bisphosphate 3-kinase catalytic subunit gamma isoform**	0.67	0.533	0.842	0.116	0.000579014	0.00430928
**Protein S100-P**	1.528	1.2	1.947	0.123	0.000586354	0.004357396
**Erythropoietin receptor**	1.499	1.19	1.89	0.118	0.000597891	0.004436514
**Pregnancy-specific beta-1-glycoprotein 8**	1.494	1.188	1.878	0.117	0.000600476	0.004449063
**3-oxo-5-beta-steroid 4-dehydrogenase**	0.733	0.614	0.876	0.0905	0.000607934	0.004497625
**Interferon alpha-2**	0.349	0.191	0.637	0.307	0.000610204	0.004501046
**Cyclin-dependent kinase 2-associated protein 1**	1.461	1.176	1.815	0.111	0.000609518	0.004501046
**Gremlin-2**	1.576	1.215	2.045	0.133	0.000616888	0.004543615
**Cystatin-S**	1.257	1.103	1.433	0.0668	0.00061835	0.004547658

**Growth factor receptor-bound protein 2**	1.145	1.059	1.237	0.0396	0.000625083	0.004590396
**Signal transducer and activator of transcription 3**	1.158	1.065	1.26	0.043	0.000632684	0.004639369
**Integrin-linked protein kinase**	1.107	1.044	1.174	0.0298	0.000637631	0.004668772
**Killer cell immunoglobulin-like receptor 2DL2**	0.559	0.401	0.781	0.17	0.000640719	0.004684494
**Microtubule-associated protein RP/EB family member 3**	1.16	1.065	1.263	0.0435	0.00064957	0.004735316
**Regulator of G-protein signaling 3**	0.533	0.371	0.765	0.185	0.000649572	0.004735316
**Calcineurin B homologous protein 3**	1.369	1.143	1.641	0.0923	0.000659092	0.004794838
**Basement membrane-specific heparan sulfate proteoglycan core protein**	1.67	1.243	2.244	0.151	0.000659663	0.004794838
**N-acetylserotonin O-methyltransferase-like protein**	1.241	1.096	1.406	0.0635	0.000661517	0.004801299
**Kininostatin**	0.791	0.691	0.905	0.0689	0.000668731	0.004846593
**Fibroblast growth factor-binding protein 3**	1.501	1.188	1.897	0.119	0.000672148	0.004857219
**NEDD8-conjugating enzyme Ubc12**	1.41	1.157	1.719	0.101	0.000671396	0.004857219
**Probable ATP-dependent RNA helicase DDX23**	0.43	0.264	0.7	0.248	0.000676744	0.004883345
**Leucine-rich repeat and transmembrane domain-containing protein 1**	1.643	1.234	2.188	0.146	0.000678022	0.004885487
**Guanine nucleotide-binding protein G(I)/G(S)/G(O) subunit gamma-11**	0.512	0.348	0.754	0.197	0.000683393	0.004903668
**Stem cell growth factor-alpha**	1.428	1.163	1.754	0.105	0.0006835	0.004903668
**Iduronate 2-sulfatase**	1.469	1.177	1.834	0.113	0.000682951	0.004903668
**Induced myeloid leukemia cell differentiation protein Mcl-1**	1.325	1.126	1.558	0.0828	0.000685392	0.004910168
**Cell adhesion molecule 2**	1.488	1.183	1.872	0.117	0.000694181	0.004965991
**Hepatoma-derived growth factor**	1.352	1.136	1.609	0.0889	0.000697721	0.004977007
**High mobility group protein B1**	0.684	0.55	0.852	0.112	0.000696998	0.004977007
**Neurogranin**	1.184	1.074	1.306	0.0499	0.0007030 1	0.005007567
**NAD kinase**	1.305	1.119	1.523	0.0787	0.000706298	0.005016633
**Thrombospondin-2**	1.397	1.151	1.695	0.0987	0.000705891	0.005016633
**3-ketodihydrosphingosine reductase**	0.58	0.423	0.795	0.161	0.000713888	0.005063313
**Stromal cell-derived factor 1**	1.595	1.217	2.09	0.138	0.000721115	0.0051073
**Leucine-rich repeat transmembrane protein FLRT2**	1.609	1.221	2.121	0.141	0.000724764	0.00512231
**Sulfhydryl oxidase 2**	0.621	0.471	0.818	0.141	0.000725292	0.00512231
**Glutathione S-transferase A4**	0.566	0.407	0.788	0.169	0.000757327	0.005340978
**Elongation factor 1-delta**	1.162	1.065	1.267	0.0445	0.000759981	0.005344556
**Repulsive guidance molecule A**	0.453	0.285	0.718	0.235	0.000759654	0.005344556
**Calcineurin subunit B type 1**	1.456	1.17	1.812	0.112	0.000767382	0.005386968
**Dual specificity protein phosphatase 3**	1.249	1.097	1.421	0.066	0.000768176	0.005386968
**Programmed cell death protein 6**	0.534	0.371	0.77	0.186	0.000770725	0.005397244

**Pleiotrophin**	1.494	1.182	1.887	0.119	0.000772358	0.005401084
**Interleukin-20 receptor subunit alpha**	1.479	1.177	1.858	0.116	0.000775069	0.005412436
**DNA-binding protein SATB2**	0.493	0.326	0.745	0.211	0.00078243	0.00545619
**Polymeric immunoglobulin receptor**	1.312	1.119	1.537	0.0809	0.000793551	0.005525999
**CD9 antigen**	0.396	0.23	0.68	0.276	0.000797961	0.005548948
**Galectin-related protein**	1.121	1.049	1.198	0.0341	0.000799393	0.005551155
**Membrane-associated guanylate kinase, WW and PDZ domain-containing protein 2**	1.374	1.141	1.655	0.095	0.000813456	0.005640942
**Thioredoxin-dependent peroxide reductase, mitochondrial**	1.146	1.058	1.241	0.0407	0.000819992	0.005678357
**72 kDa type IV collagenase**	1.829	1.284	2.605	0.18	0.000821286	0.005679418
**60 kDa heat shock protein, mitochondrial**	1.127	1.051	1.209	0.0359	0.000839902	0.005800103
**von Willebrand factor A domain-containing protein 1**	1.969	1.323	2.932	0.203	0.000845367	0.005829755
**Beta-defensin 1**	0.654	0.509	0.84	0.128	0.000869151	0.005985483
**Pleckstrin homology domain-containing family A member 1**	1.43	1.158	1.765	0.107	0.000872613	0.006001022
**Melanoma-associated antigen B10**	0.57	0.409	0.794	0.169	0.000874471	0.006005507
**Membrane-associated progesterone receptor component 1**	1.351	1.132	1.613	0.0904	0.000876074	0.006008014
**Regulator of G-protein signaling 5**	0.505	0.338	0.755	0.205	0.00087725	0.006008014
**Protein O-linked-mannose beta-1,4-N-acetylglucosaminyltransferase 2**	1.312	1.118	1.54	0.0818	0.000890373	0.006089512
**Ubiquitin carboxyl-terminal hydrolase 15**	1.334	1.125	1.582	0.087	0.00091361	0.006239869
**Caspase-3**	1.124	1.049	1.205	0.0354	0.000945077	0.006445946
**Interleukin enhancer-binding factor 3**	1.59	1.207	2.094	0.141	0.000964166	0.006567146
**Tropomodulin-2**	1.164	1.063	1.273	0.0459	0.000965926	0.006570147
**Desmin**	0.65	0.503	0.84	0.131	0.000981103	0.006664274
**Interleukin-5 receptor subunit alpha**	1.328	1.122	1.572	0.0861	0.000982866	0.006667153
**26S proteasome non-ATPase regulatory subunit 10**	0.417	0.248	0.702	0.266	0.000989719	0.006704503
**T-cell surface glycoprotein CD8 alpha chain**	1.416	1.151	1.742	0.106	0.001000474	0.00676815
**F-actin-capping protein subunit alpha-1**	1.184	1.071	1.309	0.0514	0.001003679	0.006780624
**E3 ubiquitin-protein ligase LNX**	0.572	0.41	0.798	0.17	0.001020348	0.006883895
**Folate receptor alpha**	1.318	1.118	1.555	0.0843	0.001042974	0.007027021
**PRKC apoptosis WT1 regulator protein**	1.148	1.057	1.247	0.0421	0.001051405	0.007074248
**1-phosphatidylinositol 4,5-bisphosphate phosphodiesterase beta-1**	1.229	1.086	1.391	0.063	0.001055452	0.0070919
**Interleukin-16**	1.618	1.213	2.159	0.147	0.001058162	0.007100524
**Lamina-associated polypeptide 2, isoforms beta/gamma**	1.295	1.109	1.511	0.0789	0.001064767	0.007135231
**Keratin, type I cytoskeletal 19**	0.636	0.485	0.834	0.138	0.001081115	0.007235044
**CMRF35-like molecule 8**	1.479	1.169	1.871	0.12	0.001095538	0.007321725

**Tumor necrosis factor receptor superfamily member 1B**	1.406	1.146	1.726	0.104	0.001098985	0.007334913
**Erythropoietin**	1.318	1.116	1.556	0.0847	0.001113117	0.007419291
**Desmoglein-1**	0.616	0.46	0.825	0.149	0.001131024	0.007528566
**Ribonuclease pancreatic**	1.27	1.1	1.466	0.0734	0.001135017	0.007534997
**Catenin beta-1**	0.657	0.51	0.846	0.129	0.001134331	0.007534997
**Ck-beta-8-1**	1.458	1.162	1.83	0.116	0.001142028	0.007557404
**Nuclease-sensitive element-binding protein 1**	1.317	1.116	1.554	0.0845	0.001140056	0.007557404
**Progonadoliberin-2**	1.283	1.104	1.491	0.0766	0.001142945	0.007557404
**Ras-related protein M-Ras**	0.501	0.33	0.76	0.213	0.001151719	0.007605319
**14-3-3 protein sigma**	1.439	1.155	1.792	0.112	0.001157485	0.007617727
**Calcineurin subunit B type 2**	0.62	0.465	0.827	0.147	0.001158188	0.007617727
**Polypeptide N-acetylgalactosaminyltransferase 11**	0.394	0.225	0.691	0.286	0.001156382	0.007617727
**Mitochondrial fission regulator 1**	1.424	1.15	1.762	0.109	0.00116429	0.007645656
**Protein FAM3B**	0.63	0.477	0.833	0.142	0.001165506	0.007645656
**Cadherin-15**	0.548	0.381	0.788	0.185	0.001179802	0.007720754
**Cathepsin B**	1.476	1.166	1.867	0.12	0.001181606	0.007720754
**Paralemmin-1**	0.473	0.301	0.744	0.231	0.001181211	0.007720754
**Vascular endothelial growth factor C**	1.483	1.168	1.882	0.122	0.001209656	0.007883349
**Syntenin-1**	1.49	1.17	1.897	0.123	0.001209112	0.007883349
**SLAM family member 6**	1.516	1.178	1.951	0.129	0.001216298	0.007916268
**Complement factor D**	1.889	1.284	2.779	0.197	0.001238712	0.008051628
**Histidine-rich glycoprotein**	0.708	0.573	0.873	0.107	0.00124739	0.008097462
**Gelsolin**	0.585	0.423	0.81	0.166	0.001252618	0.008120817
**B- and T-lymphocyte attenuator**	0.565	0.4	0.799	0.177	0.001258115	0.008145846
**Prefoldin subunit 3**	1.755	1.247	2.47	0.174	0.001262458	0.008163347
**Vitamin D-binding protein**	0.379	0.21	0.684	0.301	0.001273115	0.008210934
**Growth/differentiation factor 2**	0.528	0.358	0.779	0.198	0.001271899	0.008210934
**Annexin A8**	0.514	0.343	0.771	0.206	0.001274958	0.008212181
**tRNA (cytosine(38)-C(5))-methyltransferase**	1.518	1.178	1.958	0.13	0.0012794	0.008230148
**Tumor necrosis factor receptor superfamily member 11B**	1.441	1.154	1.8	0.113	0.001282981	0.00823191
**PR domain zinc finger protein 1**	0.551	0.383	0.792	0.185	0.00128266	0.00823191
**C-C motif chemokine 21**	1.472	1.163	1.862	0.12	0.001288931	0.008259444
**SUMO-conjugating enzyme UBC9**	1.248	1.09	1.428	0.0689	0.001301285	0.008317198
**Myelin protein zero-like protein 2**	0.621	0.465	0.83	0.148	0.001300449	0.008317198

**Transgelin-3**	1.37	1.131	1.66	0.0979	0.00131191	0.008369156
**Transcription factor IIIB 90 kDa subunit**	0.546	0.378	0.79	0.188	0.0013127776	0.008369156
**Ephrin type-A receptor 3**	1.325	1.116	1.574	0.0877	0.0013325061	0.008436673
**Cytokine-like protein 1**	1.321	1.115	1.566	0.0868	0.0013328491	0.008436934
**Lymphatic vessel endothelial hyaluronic acid receptor 1**	0.588	0.425	0.813	0.165	0.0013327802	0.008436934
**Alcohol dehydrogenase 1B**	0.635	0.481	0.838	0.141	0.0013333123	0.008455564
**T-cell surface glycoprotein CD3 epsilon chain**	0.664	0.516	0.853	0.128	0.0013338407	0.008478278
**Nuclear protein MDM1**	1.531	1.18	1.986	0.133	0.0013349615	0.008538414
**Inter-alpha-trypsin inhibitor heavy chain H4**	2.333	1.39	3.916	0.264	0.0013352219	0.008544036
**Alpha- and gamma-adaptin-binding protein p34**	1.194	1.071	1.331	0.0554	0.0013362867	0.008600401
**Aurora kinase A**	0.547	0.378	0.791	0.188	0.0013369223	0.00861876
**Retinoic acid early transcript 1G protein**	0.577	0.412	0.808	0.172	0.0013369239	0.00861876
**Fatty acid-binding protein, heart**	1.413	1.143	1.747	0.108	0.0013380407	0.00865763
**Serine protease HTRA2, mitochondrial**	1.295	1.105	1.517	0.0808	0.0013380811	0.00865763
**Brain natriuretic peptide 32**	1.518	1.175	1.96	0.13	0.0013385788	0.00865763
**Insulin-like growth factor 1 receptor**	1.586	1.196	2.105	0.144	0.0013379493	0.00865763
**Golgi SNAP receptor complex member 1**	0.462	0.288	0.741	0.242	0.0013382656	0.00865763
**Alkaline phosphatase, placental-like**	1.439	1.151	1.799	0.114	0.0013385847	0.00865763
**Matrilin-3**	1.471	1.161	1.863	0.121	0.0013388904	0.008665858
**Disintegrin and metalloproteinase domain-containing protein 10**	0.372	0.203	0.682	0.309	0.0013392309	0.008676231
**Glutamate receptor 4**	1.334	1.118	1.592	0.0902	0.0013397062	0.008690667
**Probable ATP-dependent RNA helicase DHX58**	1.411	1.142	1.743	0.108	0.0013398117	0.008690667
**Peroxiredoxin-1**	1.301	1.107	1.529	0.0824	0.0014001403	0.008700234
**Sex hormone-binding globulin**	1.278	1.099	1.485	0.0767	0.0014007514	0.008710812
**Protein NDNF**	0.563	0.396	0.801	0.18	0.0014006471	0.008710812
**Gremlin-1**	1.408	1.141	1.736	0.107	0.0014008356	0.008710812
**Ras-related protein Rab-14**	1.33	1.116	1.584	0.0893	0.0014026951	0.008793056
**Alpha-2,8-sialyltransferase 8F**	0.49	0.316	0.76	0.224	0.0014026812	0.008793056
**Axin-2**	0.642	0.489	0.843	0.139	0.0014024662	0.008793056
**Non-histone chromosomal protein HMG-14**	0.646	0.493	0.845	0.137	0.0014030556	0.008804372
**Proteasome subunit beta type-1**	1.455	1.155	1.832	0.118	0.00143476	0.008819343
**Integral membrane protein 2A**	0.495	0.322	0.763	0.22	0.0014043098	0.008859658
**Cellular tumor antigen p53**	1.247	1.089	1.429	0.0694	0.00144738	0.008875009
**Sarcalumenin**	1.478	1.162	1.881	0.123	0.0014056632	0.008910204

**Zymogen granule membrane protein 16**	1.117	1.044	1.196	0.0348	0.001456699	0.008910204
**Ecto-ADP-ribosyltransferase 3**	0.645	0.492	0.845	0.138	0.001463387	0.008940128
**Actin-related protein 2/3 complex subunit 1B**	1.121	1.045	1.203	0.0359	0.001490653	0.009095539
**Alpha-amylase 2B**	0.74	0.615	0.891	0.0946	0.001497912	0.009128643
**40S ribosomal protein S10**	0.732	0.603	0.887	0.0984	0.001505839	0.009165739
**Thioredoxin-related transmembrane protein 2**	0.665	0.516	0.855	0.129	0.001512314	0.009193911
**Bcl-2-like protein 1**	0.683	0.539	0.864	0.12	0.001517385	0.00921349
**Paired immunoglobulin-like type 2 receptor alpha**	1.415	1.142	1.754	0.11	0.001531343	0.009286914
**Neuroligin-1**	1.642	1.208	2.233	0.157	0.001545198	0.009348163
**ADAMTS-like protein 2**	1.424	1.144	1.773	0.112	0.001543398	0.009348163
**Scavenger receptor class F member 1**	1.406	1.139	1.737	0.108	0.001549638	0.009363651
**Adenylosuccinate synthetase isozyme 2**	1.296	1.104	1.523	0.0821	0.001564674	0.009443045
**Transferrin receptor protein 1**	1.482	1.161	1.892	0.124	0.001577306	0.009507756
**Phenylalanine--tRNA ligase, mitochondrial**	0.441	0.265	0.733	0.259	0.001592725	0.009589089
**Normal mucosa of esophagus-specific gene 1 protein**	0.729	0.599	0.887	0.1	0.001608123	0.009670102
**Interleukin-36 gamma**	1.281	1.098	1.494	0.0785	0.00161037	0.009671935
**R-spondin-3**	1.439	1.147	1.805	0.116	0.001641976	0.00984988
**Transcription factor TFIIIB component B'' homolog**	0.522	0.348	0.782	0.207	0.001644238	0.009851576
**Protein Wnt-5a**	1.365	1.124	1.657	0.0989	0.001656562	0.009913491
**Allograft inflammatory factor 1-like**	1.623	1.2	2.194	0.154	0.001659331	0.00991814
**5'(3')-deoxyribonucleotidase, cytosolic type**	1.302	1.105	1.536	0.084	0.001672014	0.009981962
**Insulin-like growth factor-binding protein 4**	1.947	1.285	2.95	0.212	0.001677205	0.009990862
**Histone deacetylase complex subunit SAP18**	0.631	0.474	0.841	0.146	0.001677518	0.009990862
**Calcineurin B homologous protein 1**	1.346	1.118	1.62	0.0946	0.001689896	0.010052559
**Immunoglobulin A**	1.322	1.11	1.574	0.089	0.001715953	0.010195379
**Urea transporter 1**	0.452	0.275	0.742	0.254	0.001718001	0.010195384
**Glioma pathogenesis-related protein 1**	1.47	1.155	1.871	0.123	0.001732276	0.010267861
**Transaldolase**	1.438	1.145	1.805	0.116	0.00175	0.010413864
**Bone morphogenetic protein 8B**	0.511	0.335	0.778	0.215	0.001773404	0.010486674
**Carboxypeptidase A2**	0.623	0.463	0.839	0.151	0.001782785	0.010529642
**Beta-1,4-galactosyltransferase 1**	2.171	1.334	3.532	0.248	0.001800402	0.010608521
**Matrix metalloproteinase-20**	0.612	0.45	0.833	0.157	0.001798491	0.010608521
**Fibroblast growth factor 7**	1.367	1.123	1.664	0.1	0.001832551	0.010768894
**Interleukin-10 receptor subunit alpha**	0.581	0.413	0.818	0.174	0.001834108	0.010768894

**Ribokinase**	0.507	0.331	0.777	0.218	0.001830302	0.010768894
**Cytochrome P450 3A4**	0.462	0.284	0.751	0.248	0.001838977	0.010784768
**Pro-opiomelanocortin**	1.368	1.123	1.666	0.101	0.001848124	0.010812934
**Ras-related protein Rab-27B**	0.492	0.315	0.769	0.228	0.001846135	0.010812934
**PCNA-associated factor**	1.336	1.113	1.604	0.0932	0.00185847	0.010860705
**Hepatitis A virus cellular receptor 2**	1.537	1.172	2.016	0.138	0.001877623	0.010959773
**ADP-ribosylation factor 6**	0.461	0.283	0.752	0.249	0.001898731	0.01107
**Angiogenin**	1.575	1.182	2.099	0.146	0.00191207	0.011134732
**t-SNARE domain-containing protein 1**	0.365	0.193	0.69	0.325	0.001916664	0.011148449
**Uncharacterized protein KIAA1644**	1.29	1.098	1.514	0.082	0.001930183	0.01121398
**Ecto-NOX disulfide-thiol exchanger 1**	0.711	0.572	0.882	0.11	0.001936875	0.011239744
**Glycoprotein hormone alpha-2**	0.535	0.36	0.795	0.202	0.001951939	0.011313972
**Carboxypeptidase N catalytic chain**	0.462	0.283	0.753	0.25	0.001963406	0.011367206
**Calmegin**	1.32	1.107	1.574	0.0897	0.001972792	0.011408285
**Basal Cell Adhesion Molecule**	1.473	1.152	1.884	0.125	0.001993387	0.011514007
**Serine/threonine-protein kinase DCLK1**	1.39	1.128	1.712	0.107	0.00200686	0.01156561
**Gastrin-releasing peptide**	1.442	1.143	1.819	0.118	0.002006967	0.01156561
**Calcium-activated potassium channel subunit beta-3**	1.765	1.23	2.531	0.184	0.002030063	0.011685183
**IQ domain-containing protein F3**	0.502	0.324	0.778	0.224	0.002033433	0.011691067
**Peptidyl-prolyl cis-trans isomerase F, mitochondrial**	1.098	1.035	1.166	0.0305	0.00204623	0.01175107
**ICOS ligand**	1.316	1.105	1.567	0.0891	0.002050067	0.011759543
**Protocadherin gamma-A12**	1.478	1.153	1.896	0.127	0.002060178	0.011803945
**Fibroblast growth factor 18**	1.334	1.11	1.602	0.0935	0.002064441	0.011814773
**Regulator of G-protein signaling 10**	1.133	1.046	1.226	0.0405	0.002083539	0.011910378
**SLIT and NTRK-like protein 4**	1.567	1.177	2.086	0.146	0.002100318	0.011975373
**PDZ and LIM domain protein 1**	1.117	1.041	1.199	0.036	0.002103753	0.011975373
**Chitinase-3-like protein 2**	0.444	0.265	0.745	0.264	0.002104529	0.011975373
**Serine protease HTRA1**	1.686	1.209	2.353	0.17	0.002103626	0.011975373
**Stem cell growth factor-beta**	1.446	1.143	1.83	0.12	0.002107151	0.011976602
**V-type immunoglobulin domain-containing suppressor of T-cell activation**	1.33	1.109	1.595	0.0927	0.002123912	0.012058105
**Small VCP/p97-interacting protein**	0.535	0.359	0.798	0.203	0.002132483	0.012092977
**Lysosomal Pro-X carboxypeptidase**	0.646	0.488	0.854	0.142	0.002138631	0.012102111
**Netrin receptor UNC5C**	1.638	1.195	2.245	0.161	0.002138955	0.012102111
**Tyrosine-protein kinase CSK**	1.133	1.046	1.228	0.0408	0.002149935	0.012150425

**Citrate synthase, mitochondrial**	1.107	1.037	1.181	0.0331	0.002157981	0.012168277
**ES1 protein homolog, mitochondrial**	0.526	0.349	0.793	0.209	0.002156257	0.012168277
**Thyroid peroxidase**	0.659	0.505	0.86	0.136	0.002169221	0.012217817
**Fibroleukin**	0.558	0.384	0.81	0.191	0.002175301	0.012224871
**Interleukin-10**	1.351	1.115	1.638	0.0982	0.002175384	0.012224871
**Palmitoyl-protein thioesterase 1**	1.395	1.128	1.727	0.109	0.002179512	0.01223426
**Heparan-sulfate 6-O-sulfotransferase 3**	0.444	0.264	0.746	0.265	0.00218481	0.012250191
**Legumain**	0.553	0.379	0.808	0.193	0.002196311	0.012300824
**Heterogeneous nuclear ribonucleoprotein Q**	0.681	0.533	0.871	0.125	0.002214935	0.012391192
**Metalloproteinase inhibitor 2**	1.935	1.267	2.956	0.216	0.002267341	0.012655932
**Retinoid-binding protein 7**	0.582	0.411	0.824	0.177	0.002266632	0.012655932
**Annexin A11**	1.137	1.047	1.235	0.0421	0.002271128	0.012662874
**Leucine-rich repeat-containing protein 4C**	1.572	1.175	2.102	0.148	0.002290367	0.012755861
**OCIA domain-containing protein 1**	0.612	0.446	0.839	0.161	0.002294392	0.012763997
**Coagulation factor IXab**	0.702	0.559	0.881	0.116	0.002298696	0.012773667
**Nidogen-1**	1.561	1.172	2.078	0.146	0.002306979	0.012805406
**Coiled-coil domain-containing protein 50**	1.242	1.08	1.427	0.071	0.002316078	0.012806756
**Ubiquitin-fold modifier 1**	1.178	1.06	1.308	0.0537	0.002322655	0.012806756
**ATPase WRNIP1**	0.383	0.206	0.71	0.315	0.002317622	0.012806756
**Immunoglobulin superfamily member 3**	1.446	1.14	1.832	0.121	0.002319542	0.012806756
**Calsyntenin-2**	1.407	1.13	1.753	0.112	0.002314185	0.012806756
**Seizure 6-like protein**	0.556	0.382	0.811	0.192	0.002321445	0.012806756
**Retinoic acid receptor responder protein 3**	1.302	1.099	1.544	0.0868	0.002330452	0.012835529
**Coxsackievirus and adenovirus receptor**	0.648	0.491	0.857	0.142	0.002340996	0.012879357
**Protein dpy-30 homolog**	1.366	1.117	1.671	0.103	0.0023828	0.013094881
**Segment polarity protein dishevelled homolog DVL-2**	0.677	0.527	0.871	0.128	0.002422555	0.013298681
**Inhibin beta C chain**	0.629	0.466	0.849	0.153	0.002440172	0.013380634
**Golgi membrane protein 1**	1.308	1.1	1.557	0.0887	0.002449203	0.013415382
**Armadillo repeat-containing protein 5**	0.475	0.294	0.769	0.245	0.002452768	0.013420143
**Neurotrimin**	1.485	1.15	1.919	0.131	0.002458886	0.013438853
**Tumor necrosis factor receptor superfamily member 19**	1.366	1.116	1.673	0.103	0.002486233	0.013499406
**Macrophage scavenger receptor types I and II**	1.358	1.114	1.656	0.101	0.002480863	0.013499406
**Argininosuccinate lyase**	0.805	0.7	0.927	0.0715	0.002477953	0.013499406
**Vacuolar protein-sorting-associated protein 25**	1.178	1.059	1.309	0.054	0.002479428	0.013499406

**Magnesium transporter NIPA4**	0.618	0.452	0.844	0.159	0.002480005	0.013499406
**Transmembrane protein 132C**	1.461	1.143	1.867	0.125	0.00248471	0.013499406
**Sulfotransferase 1C2**	0.543	0.366	0.807	0.202	0.002491979	0.013515865
**Interleukin-1 receptor-like 1**	1.343	1.109	1.626	0.0975	0.002500399	0.013546773
**C-C motif chemokine 14**	1.348	1.111	1.635	0.0987	0.002508487	0.013575629
**Tumor necrosis factor-inducible gene 6 protein**	1.422	1.132	1.787	0.117	0.002513904	0.013575629
**Stabilin-2**	0.622	0.457	0.846	0.157	0.002511382	0.013575629
**Ig Kappa chain V-I region HK102- like**	1.451	1.139	1.849	0.124	0.00256347	0.013828297
**C-type lectin domain family 2 member D**	0.515	0.335	0.793	0.22	0.002588345	0.013932294
**Hydroxymethylglutaryl-CoA synthase, cytoplasmic**	0.866	0.788	0.951	0.0478	0.00258767	0.013932294
**Protein disulfide-isomerase TMX3**	1.486	1.148	1.923	0.132	0.002606239	0.013998345
**Nucleophosmin**	0.347	0.174	0.691	0.351	0.002605011	0.013998345
**Calcium-regulated heat-stable protein 1**	1.358	1.112	1.658	0.102	0.002637913	0.014122761
**Sorting nexin-7**	0.657	0.5	0.864	0.14	0.002634516	0.014122761
**Glypican-5**	0.675	0.522	0.872	0.131	0.002636732	0.014122761
**Inositol monophosphatase 3**	0.668	0.514	0.869	0.134	0.002657165	0.014210553
**Microtubule-associated proteins 1A/1B light chain 3B**	1.222	1.072	1.392	0.0667	0.00266087	0.014215097
**SH3 domain-binding glutamic acid-rich-like protein 3**	1.14	1.047	1.242	0.0437	0.002670815	0.014252934
**Protein RIC-3**	0.422	0.24	0.741	0.288	0.002684813	0.014312294
**Tenascin**	1.32	1.101	1.582	0.0925	0.002693276	0.014342053
**Alpha-1-antitrypsin**	1.539	1.161	2.04	0.144	0.002702738	0.014346409
**Major prion protein**	0.54	0.362	0.808	0.205	0.002698413	0.014346409
**Apolipoprotein C-III**	0.851	0.766	0.946	0.0538	0.002700634	0.014346409
**Neutrophil cytosol factor 4**	0.722	0.584	0.893	0.109	0.002710932	0.014359289
**E3 ubiquitin-protein ligase RNF146**	0.425	0.243	0.743	0.286	0.002709967	0.014359289
**Stromelysin-2**	1.36	1.112	1.663	0.103	0.002718283	0.014382924
**Collagen type IV alpha-3-binding protein**	1.199	1.065	1.351	0.0607	0.002742471	0.014495502
**M-phase inducer phosphatase 2**	0.506	0.324	0.79	0.227	0.002747496	0.014506661
**Platelet-activating factor acetylhydrolase**	1.466	1.141	1.883	0.128	0.002779028	0.014657607
**Serine/threonine-protein kinase pim-1**	0.519	0.338	0.798	0.219	0.00278544	0.014675881
**Methionine aminopeptidase 1**	1.113	1.038	1.194	0.0359	0.002820322	0.014843959
**Fibroblast growth factor receptor 3**	0.573	0.397	0.827	0.187	0.002900129	0.015247878
**Lymphokine-activated killer T-cell-originated protein kinase**	0.495	0.312	0.787	0.236	0.002903986	0.015252054
**Transcobalamin-1**	1.275	1.086	1.496	0.0817	0.002927301	0.015358304

**Nucleosome assembly protein 1-like 2**	0.581	0.406	0.831	0.183	0.002936258	0.015389084
**Chordin-like protein 1**	1.578	1.168	2.131	0.153	0.00295285	0.015459769
**Protocadherin gamma-A10**	1.291	1.091	1.529	0.086	0.002957308	0.015466845
**Apolipoprotein D**	0.656	0.496	0.866	0.142	0.002987765	0.01560974
**Tyrosine-protein kinase SYK**	1.114	1.037	1.197	0.0365	0.003021872	0.015771384
**Ribosomal protein S6 kinase alpha-3**	1.149	1.048	1.26	0.047	0.003031844	0.015806859
**Regenerating islet-derived protein 3-alpha**	1.261	1.082	1.47	0.0783	0.003038042	0.015822368
**Estradiol 17-beta-dehydrogenase 8**	0.729	0.592	0.899	0.107	0.003041174	0.015822368
**Dual specificity protein phosphatase 15**	1.238	1.075	1.426	0.0722	0.003051816	0.015861161
**Alpha-hemoglobin-stabilizing protein**	0.685	0.534	0.88	0.128	0.003077397	0.015977436
**V-set and transmembrane domain-containing protein 4**	0.52	0.337	0.802	0.221	0.003085116	0.016000826
**Vacuolar protein sorting-associated protein 26A**	1.186	1.059	1.329	0.0578	0.003136198	0.016248835
**HLA class I histocompatibility antigen, alpha chain G**	1.661	1.186	2.327	0.172	0.00314254	0.016264767
**AP-2 complex subunit alpha-2**	1.195	1.062	1.345	0.0604	0.003155272	0.016313708
**Low-density lipoprotein receptor-related protein 11**	1.733	1.203	2.498	0.187	0.003180352	0.016391274
**Histone acetyltransferase type B catalytic subunit**	0.672	0.516	0.875	0.135	0.003181727	0.016391274
**GrpE protein homolog 1, mitochondrial**	1.183	1.058	1.324	0.0571	0.003183443	0.016391274
**Growth/differentiation factor 10**	0.504	0.32	0.795	0.232	0.003181781	0.016391274
**O-acetyl-ADP-ribose deacetylase MACROD1**	0.586	0.411	0.836	0.181	0.00319945	0.016456678
**Cathelicidin antimicrobial peptide**	0.786	0.669	0.922	0.0819	0.003212261	0.016504058
**Ubiquitin carboxyl-terminal hydrolase 21**	0.615	0.445	0.85	0.165	0.003215292	0.016504058
**Transcription factor RelB**	0.535	0.353	0.811	0.213	0.003232998	0.016577855
**Tryptophan--tRNA ligase, cytoplasmic**	1.493	1.143	1.949	0.136	0.003256041	0.016678837
**Thioredoxin domain-containing protein 12**	1.161	1.051	1.282	0.0507	0.003278439	0.016776306
**Acidic leucine-rich nuclear phosphoprotein 32 family member A**	0.542	0.36	0.815	0.208	0.003294586	0.016841628
**Homeodomain-only protein**	0.654	0.493	0.868	0.145	0.003320098	0.016954632
**Pancreatic alpha-amylase**	0.769	0.646	0.917	0.0893	0.003335128	0.017013938
**Transmembrane protein 59-like**	1.618	1.173	2.233	0.164	0.003356376	0.017104808
**Prothrombin**	2.072	1.273	3.373	0.249	0.003375833	0.017186375
**Proteasome subunit alpha type-2**	0.658	0.497	0.87	0.143	0.003395656	0.017269632
**Peroxisomal membrane protein PEX14**	1.262	1.08	1.474	0.0794	0.003407864	0.017296388
**Protein regulator of cytokinesis 1**	0.56	0.379	0.825	0.198	0.003404988	0.017296388
**Asporin**	0.758	0.63	0.913	0.0946	0.003431973	0.017401011
**Eukaryotic translation initiation factor 2 subunit 1**	1.166	1.052	1.292	0.0525	0.003442929	0.017438802

**Torsin-1A-interacting protein 2**	1.307	1.092	1.565	0.0917	0.003475207	0.017571097
**Eukaryotic translation initiation factor 4E**	1.224	1.069	1.402	0.0692	0.003476106	0.017571097
**Serine protease inhibitor Kazal-type 4**	0.565	0.385	0.829	0.196	0.003563714	0.01799567
**Catalase**	0.789	0.672	0.925	0.0815	0.003589173	0.018105869
**Methyl-CpG-binding domain protein 1**	0.744	0.61	0.908	0.101	0.003624272	0.018264425
**Neutral ceramidase**	0.699	0.549	0.89	0.123	0.003642631	0.01833838
**Aldose reductase**	1.31	1.092	1.573	0.0932	0.003728291	0.018703772
**C-C motif chemokine 23**	1.495	1.139	1.962	0.139	0.003731778	0.018703772
**Bone sialoprotein 2**	1.303	1.09	1.559	0.0913	0.00372795	0.018703772
**Tomoregulin-1**	1.569	1.157	2.128	0.155	0.003720462	0.018703772
**Sialoadhesin**	1.275	1.082	1.502	0.0837	0.003733993	0.018703772
**Adseverin**	1.361	1.105	1.676	0.106	0.003768049	0.018855393
**Bicaudal D-related protein 1**	1.48	1.135	1.93	0.135	0.003785363	0.018923013
**Switch-associated protein 70**	1.538	1.149	2.058	0.149	0.003810218	0.019028159
**Soluble calcium-activated nucleotidase 1**	1.267	1.079	1.487	0.0818	0.0038434 4	0.019155645
**Advanced glycosylation end product-specific receptor, soluble**	1.19	1.058	1.338	0.0601	0.0038 4308	0.019155645
**Activin B**	0.637	0.469	0.865	0.156	0.003912948	0.019463104
**Collagen alpha-1(VI) chain**	1.198	1.06	1.355	0.0627	0.003911155	0.019463104
**Proteasome subunit alpha type-5**	0.729	0.588	0.904	0.109	0.003924643	0.019501794
**Alpha-actinin-2**	1.17	1.052	1.302	0.0545	0.003952845	0.019542394
**UDP-glucose 4-epimerase**	0.675	0.517	0.882	0.136	0.003943878	0.019542394
**E3 ubiquitin-protein ligase ZNRF3**	1.283	1.083	1.52	0.0865	0.003954587	0.019542394
**Ectodysplasin-A, secreted form**	0.587	0.408	0.843	0.185	0.003960288	0.019542394
**Proto-oncogene tyrosine-protein kinase ROS**	0.63	0.46	0.863	0.16	0.003952265	0.019542394
**Transmembrane protein 132A**	0.722	0.579	0.901	0.113	0.003950097	0.019542394
**Kit ligand**	0.687	0.532	0.887	0.131	0.003958847	0.019542394
**ER membrane protein complex subunit 4**	0.602	0.426	0.85	0.177	0.0039958	0.019690366
**Tubulin-specific chaperone A**	1.333	1.096	1.622	0.0999	0.003998184	0.019690366
**Ubiquitin-associated and SH3 domain-containing protein B**	1.08	1.025	1.138	0.0268	0.004019242	0.019768727
**Alpha-1-antichymotrypsin complex**	1.332	1.096	1.619	0.0997	0.004022037	0.019768727
**Amiloride-sensitive amine oxidase [copper-containing]**	0.827	0.727	0.941	0.0659	0.0040 2648	0.019771048
**Interleukin-3 receptor subunit alpha**	1.368	1.105	1.694	0.109	0.004070529	0.019947996
**Erlin-1**	0.431	0.242	0.765	0.293	0.004068696	0.019947996
**Glypican-4**	1.319	1.092	1.594	0.0965	0.004076665	0.019958422

**Kin of IRRE-like protein 3**	1.701	1.184	2.444	0.185	0.004082132	0.019965554
**DnaJ homolog subfamily B member 1**	1.157	1.047	1.278	0.0508	0.004104542	0.020055459
**Sialic acid-binding Ig-like lectin 15**	0.471	0.281	0.788	0.263	0.00416077	0.020310267
**Protein S100-A10**	0.562	0.379	0.834	0.201	0.004186389	0.02041531
**Heparan sulfate glucosamine 3-O-sulfotransferase 5**	0.692	0.538	0.891	0.128	0.004193547	0.020430208
**Serine/threonine-protein phosphatase 4 regulatory subunit 3A**	1.342	1.097	1.642	0.103	0.004224032	0.020558609
**Kidney-associated antigen 1**	1.431	1.119	1.83	0.126	0.004316714	0.020989178
**Delta and Notch-like epidermal growth factor-related receptor**	0.703	0.552	0.896	0.123	0.004323089	0.02099967
**L-Selectin**	0.585	0.405	0.846	0.188	0.004343494	0.021078221
**Interferon lambda receptor 1**	1.44	1.12	1.849	0.128	0.004380084	0.021235089
**Vesicle-associated membrane protein-associated protein A**	1.133	1.04	1.235	0.0439	0.004389014	0.021237026
**Indian hedgehog protein**	0.697	0.543	0.893	0.127	0.004388426	0.021237026
**Insulin-like 3**	0.673	0.513	0.884	0.139	0.004397748	0.021258627
**Tyrosine-protein kinase Lyn, isoform B**	1.109	1.033	1.192	0.0365	0.004457787	0.021527955
**Protein SGT1 homolog**	1.176	1.052	1.315	0.057	0.004466899	0.021530194
**Uncharacterized protein C17orf78**	0.666	0.503	0.881	0.143	0.004462604	0.021530194
**Ubiquitin-like protein 4A**	1.175	1.051	1.313	0.0567	0.004520602	0.021767966
**Ras GTPase-activating protein-binding protein 2**	1.154	1.045	1.273	0.0503	0.004527171	0.021778535
**Ectodysplasin-A, secreted form**	0.55	0.363	0.831	0.211	0.004578882	0.022006037
**Grancalcin**	1.238	1.068	1.435	0.0753	0.004592994	0.02205257
**Torsin-1A-interacting protein 1**	1.217	1.062	1.395	0.0694	0.004610405	0.022114843
**Glutaredoxin-like protein C5orf63**	0.547	0.36	0.83	0.213	0.004623299	0.022155348
**C-C motif chemokine 22**	1.265	1.075	1.489	0.0831	0.004646347	0.022244386
**Carbonic anhydrase 13**	1.101	1.03	1.177	0.034	0.00467731	0.02237111
**Thyroid hormone receptor alpha**	0.601	0.422	0.855	0.18	0.004685682	0.022389647
**Eukaryotic translation initiation factor 4B**	1.166	1.048	1.296	0.0542	0.004703059	0.022451134
**Amyloid beta A4 precursor protein-binding family B member 1**	1.321	1.089	1.603	0.0986	0.004720638	0.02246954
**Potassium voltage-gated channel subfamily E regulatory beta subunit 5**	1.291	1.081	1.541	0.0904	0.004713069	0.02246954
**Sclerostin**	0.594	0.414	0.852	0.184	0.0047202	0.02246954
**Matrix-remodeling-associated protein 8**	0.591	0.411	0.851	0.186	0.004724967	0.02246954
**Ena/VASP-like protein**	0.634	0.462	0.87	0.162	0.004745378	0.022545075
**Aldo-keto reductase family 1 member C1**	0.596	0.417	0.854	0.183	0.004751193	0.022551183
**Phosphoglycerate kinase 1**	1.245	1.069	1.45	0.0777	0.004759943	0.022571195
**Carnitine O-acetyltransferase**	1.1	1.03	1.176	0.0339	0.004806567	0.022770598

**SAYSvFN domain-containing protein 1**	0.62	0.444	0.865	0.17	0.00484516	0.022931608
**DNA-directed RNA polymerase III subunit RPC9**	0.588	0.406	0.851	0.189	0.004895798	0.023128232
**WW domain-binding protein 2**	1.262	1.073	1.484	0.0827	0.004895995	0.023128232
**Ferritin, mitochondrial**	1.33	1.09	1.623	0.101	0.004909653	0.023159684
**GDNF family receptor alpha-2**	0.566	0.38	0.841	0.203	0.004911955	0.023159684
**Tapasin**	1.251	1.07	1.463	0.0797	0.004918999	0.023170953
**Myosin regulatory light chain 12A**	1.108	1.032	1.19	0.0365	0.004949987	0.023294882
**Protocadherin beta-10**	0.645	0.475	0.876	0.156	0.00499645	0.023469175
**Receptor tyrosine-protein kinase erbB-4**	1.354	1.096	1.673	0.108	0.004995077	0.023469175
**Diphosphoinositol polyphosphate phosphohydrolase 1**	1.261	1.072	1.483	0.0828	0.005076145	0.023809762
**Acid sphingomyelinase-like phosphodiesterase 3a**	0.64	0.468	0.874	0.16	0.005078523	0.023809762
**E3 ubiquitin-protein ligase RNF128**	0.524	0.333	0.823	0.231	0.005084336	0.023814589
**Eukaryotic translation initiation factor 4 gamma 2**	1.084	1.025	1.147	0.0288	0.005118825	0.023953599
**Neuropilin-1**	1.686	1.169	2.431	0.187	0.005173693	0.024187621
**DNA-binding protein SATB1**	0.576	0.392	0.848	0.197	0.005188419	0.024233715
**3-ketoacyl-CoA thiolase, peroxisomal**	0.835	0.737	0.948	0.0643	0.005196997	0.024251027
**BH3-interacting domain death agonist**	1.252	1.069	1.466	0.0804	0.005227061	0.024347261
**Thiamine-triphosphatase**	0.69	0.532	0.895	0.133	0.0052274	0.024347261
**Netrin receptor UNC5A**	1.247	1.068	1.456	0.079	0.005240426	0.024385125
**Heparan sulfate glucosamine 3-O-sulfotransferase 1**	0.472	0.278	0.799	0.269	0.005245838	0.024387516
**Beta-1,4-galactosyltransferase 6**	0.651	0.481	0.88	0.154	0.005257816	0.024420398
**Quinone oxidoreductase-like protein 1**	0.819	0.712	0.942	0.0715	0.005264919	0.024430596
**Proto-oncogene tyrosine-protein kinase Src isoform 2**	1.09	1.026	1.158	0.0309	0.005288331	0.02449907
**Dickkopf-like protein 1**	0.693	0.535	0.897	0.132	0.005289516	0.02449907
**Stanniocalcin-1**	1.518	1.132	2.037	0.15	0.005305475	0.024550151
**Quinone oxidoreductase**	0.845	0.75	0.951	0.0606	0.005371611	0.024833101
**Protein kinase C beta type (splice variant beta-II)**	1.101	1.029	1.178	0.0346	0.005462899	0.025231703
**Retinoschisin**	0.553	0.364	0.84	0.213	0.005488346	0.025325743
**VPS10 domain-containing receptor SorCS2**	0.855	0.765	0.955	0.0565	0.005518162	0.025439751
**Cas scaffolding protein family member 4**	1.324	1.086	1.614	0.101	0.005556853	0.025594425
**Myoneurin**	0.533	0.342	0.832	0.227	0.005562557	0.025597016
**Nectin-3**	0.49	0.296	0.812	0.257	0.005592864	0.025712715
**Attractin**	0.521	0.328	0.826	0.235	0.005599077	0.025717532
**Adenylyl cyclase-associated protein 1**	1.094	1.027	1.166	0.0324	0.00563525	0.025838724

**Dual adapter for phosphotyrosine and 3-phosphotyrosine and 3-phosphoinositide**	1.089	1.025	1.157	0.0308	0.005635841	0.025838724
**Kallikrein-8**	1.491	1.124	1.977	0.144	0.005645992	0.025861449
**Serine/arginine-rich splicing factor 6**	1.423	1.108	1.827	0.128	0.005708673	0.026124527
**Rho-related GTP-binding protein RhoC**	1.122	1.034	1.217	0.0416	0.005727343	0.026185896
**UDP-glucose 6-dehydrogenase**	0.875	0.796	0.962	0.0484	0.005733927	0.026191949
**Tubulin-folding cofactor B**	1.123	1.034	1.219	0.042	0.005799262	0.026466109
**E3 ubiquitin-protein ligase RNF34**	0.598	0.415	0.862	0.187	0.005830589	0.026584709
**Secretogranin-3**	0.659	0.49	0.887	0.151	0.005883714	0.026802389
**Matrilin-2**	1.843	1.192	2.848	0.222	0.005955217	0.027046125
**Beta-2-glycoprotein 1**	1.911	1.205	3.03	0.235	0.005947327	0.027046125
**NADPH--cytochrome P450 reductase**	0.77	0.64	0.928	0.0949	0.005955984	0.027046125
**Dipeptidase 1**	1.347	1.089	1.666	0.108	0.005958948	0.027046125
**Cysteine-rich protein 2**	1.367	1.094	1.708	0.114	0.005977267	0.027104565
**Tumor susceptibility gene 101 protein**	1.121	1.033	1.216	0.0415	0.006046512	0.027393615
**Inactive peptidyl-prolyl cis-trans isomerase FKBP6**	0.696	0.538	0.902	0.132	0.006118981	0.027671575
**Secretory carrier-associated membrane protein 5**	0.5	0.305	0.821	0.253	0.006116424	0.027671575
**Enhancer of rudimentary homolog**	1.234	1.062	1.434	0.0767	0.00612802	0.027687307
**Keratocan**	1.464	1.115	1.922	0.139	0.006137469	0.027690855
**NKG2D ligand 2**	1.298	1.077	1.563	0.0951	0.006139929	0.027690855
**Low-density lipoprotein receptor-related protein 4**	1.309	1.08	1.586	0.0982	0.006150608	0.027713916
**Apolipoprotein E**	0.812	0.7	0.943	0.076	0.006160383	0.027732862
**Agouti-related protein**	1.398	1.1	1.777	0.122	0.006165956	0.027732878
**Vitamin K-dependent protein S**	0.641	0.466	0.881	0.163	0.006199139	0.027856963
**Serine protease inhibitor Kazal-type 7**	1.387	1.097	1.754	0.12	0.006234894	0.027967151
**Aldehyde dehydrogenase family 1 member A3**	0.845	0.749	0.953	0.0616	0.006234722	0.027967151
**Protein BTG2**	0.597	0.412	0.864	0.189	0.006298533	0.028227178
**Pyrroline-5-carboxylate reductase 1, mitochondrial**	1.248	1.065	1.463	0.0812	0.006335705	0.028368234
**Neural cell adhesion molecule 1, 120 kDa isoform**	0.593	0.407	0.863	0.192	0.006387036	0.028572374
**DNA polymerase epsilon subunit 3**	0.448	0.252	0.798	0.294	0.006399526	0.028602548
**Nuclear receptor ROR-gamma**	1.284	1.073	1.538	0.0918	0.006418906	0.028663438
**T-cell surface antigen CD2**	1.111	1.03	1.199	0.0387	0.006484144	0.028928812
**Small ubiquitin-related modifier 3**	1.158	1.042	1.287	0.0539	0.006535657	0.02913253
**Growth/differentiation factor 3**	0.569	0.379	0.854	0.208	0.006580425	0.029305848
**Interferon-induced protein with tetratricopeptide repeats 2**	0.692	0.53	0.902	0.136	0.006598211	0.029358797

**Alpha-N-acetylgalactosaminide alpha-2,6-sialyltransferase 6**	1.54	1.127	2.103	0.159	0.006650558	0.029558746
**Nicotinate-nucleotide pyrophosphorylase [carboxylating]**	1.413	1.101	1.814	0.127	0.006655022	0.029558746
**Interleukin-6 receptor subunit alpha**	0.662	0.491	0.892	0.152	0.00668293	0.029656245
**SWI/SNF-related matrix-associated actin-dependent regulator of chromatin subfamily E member 1-related**	1.346	1.086	1.668	0.11	0.006717959	0.029785145
**Protein FAM19A2**	0.679	0.513	0.898	0.143	0.006734291	0.029830994
**Ras-related protein Rab-6B**	1.095	1.025	1.169	0.0335	0.006748254	0.029866272
**Transmembrane protein 9**	1.267	1.067	1.503	0.0873	0.006790357	0.030025923
**Teneurin-2**	0.54	0.346	0.844	0.228	0.006823618	0.030146221
**BMP-binding endothelial regulator protein**	1.452	1.108	1.903	0.138	0.006835094	0.030170155
**Serine/threonine-protein kinase 24**	1.102	1.027	1.183	0.036	0.006847198	0.030196809
**Collagen alpha-1(X) chain**	1.211	1.054	1.391	0.0709	0.006925991	0.030517263
**ETS-related transcription factor Elf-5**	1.43	1.103	1.854	0.133	0.006933606	0.030523806
**Solute carrier family 41 member 2**	0.686	0.521	0.902	0.14	0.006946386	0.030553053
**Semaphorin-6C**	0.646	0.47	0.887	0.162	0.0069716	0.03063689
**Protachykinin-1**	1.339	1.083	1.655	0.108	0.006986972	0.030677368
**Apolipoprotein C-I**	2.071	1.22	3.514	0.27	0.006994275	0.030682374
**Succinate-semialdehyde dehydrogenase, mitochondrial**	0.798	0.677	0.94	0.0839	0.007027512	0.030801039
**Ornithine decarboxylase antizyme 1**	1.242	1.061	1.454	0.0805	0.007047185	0.030860099
**Serine protease inhibitor Kazal-type 2**	1.255	1.064	1.481	0.0844	0.007055933	0.030871258
**Ornithine carbamoyltransferase, mitochondrial**	0.864	0.777	0.961	0.0541	0.007078471	0.030942677
**Nucleoside diphosphate kinase B**	1.114	1.03	1.205	0.0401	0.007091072	0.030970568
**Kelch-like protein 12**	1.305	1.075	1.584	0.0988	0.007117936	0.031060653
**C-C motif chemokine 4-like**	1.221	1.056	1.412	0.0741	0.007128659	0.031080203
**Stromal cell-derived factor 1**	1.378	1.091	1.741	0.119	0.007159677	0.031188128
**Coiled-coil domain-containing protein 167**	0.64	0.462	0.886	0.166	0.007240127	0.031511009
**Kynureninase**	0.647	0.471	0.889	0.162	0.007292708	0.031684461
**GDP-fucose protein O-fucosyltransferase 1**	1.315	1.077	1.606	0.102	0.007288407	0.031684461
**Macrophage receptor MARCO**	0.6	0.413	0.872	0.191	0.007337669	0.031852008
**Prenylcysteine oxidase 1**	0.678	0.511	0.901	0.145	0.007354335	0.031868785
**Insulin**	1.151	1.039	1.276	0.0526	0.007349955	0.031868785
**Beta-1,4-galactosyltransferase 2**	0.507	0.308	0.833	0.254	0.00736517	0.031887986
**Serine palmitoyltransferase 2**	0.667	0.496	0.897	0.151	0.007392255	0.031977444
**Myelin protein zero-like protein 1**	1.372	1.088	1.73	0.118	0.007434728	0.032133256
**BPI fold-containing family B member 1**	1.25	1.062	1.472	0.0833	0.007444069	0.032145723

**Peptidyl-prolyl cis-trans isomerase FKBP3**	1.138	1.035	1.251	0.0483	0.007473423	0.032244519
**Fc receptor-like protein 1**	1.278	1.068	1.531	0.0919	0.007561679	0.032597057
**Pancreatic triacylglycerol lipase**	0.845	0.747	0.956	0.0629	0.007574886	0.032625742
**Down syndrome cell adhesion molecule-like protein 1**	1.392	1.092	1.775	0.124	0.007622942	0.032804348
**Peroxisome assembly protein 26**	0.638	0.459	0.888	0.168	0.007696998	0.033094433
**Interleukin-12 subunit beta**	1.295	1.071	1.567	0.0971	0.007719338	0.033161851
**14-3-3 protein zeta/delta**	1.129	1.032	1.234	0.0455	0.007762833	0.033319955
**Chordin-like protein 2**	1.368	1.086	1.724	0.118	0.007799119	0.033446866
**Vacuolar protein sorting-associated protein 4B**	0.689	0.523	0.907	0.14	0.007848732	0.033630668
**Docking protein 2**	1.093	1.024	1.167	0.0335	0.00786696	0.03367979
**SHC-transforming protein 1**	1.167	1.041	1.308	0.0582	0.007895373	0.03377239
**Metalloproteinase inhibitor 4**	1.342	1.08	1.667	0.111	0.007940624	0.033936794
**DnaJ homolog subfamily B member 11**	1.267	1.064	1.51	0.0893	0.007959355	0.033987673
**Protein kinase C-binding protein NELL1**	0.775	0.642	0.936	0.096	0.008029163	0.034256387
**Eukaryotic translation initiation factor 5A-2**	1.16	1.039	1.296	0.0562	0.008066554	0.03438645
**RNA-binding protein 3**	1.219	1.053	1.411	0.0748	0.0080908	0.034466085
**Ubiquitin-conjugating enzyme E2 K**	1.178	1.044	1.33	0.0619	0.008093815	0.034466085
**Ephrin type-B receptor 1**	1.246	1.059	1.467	0.0832	0.00814008	0.034581448
**Deoxynucleoside triphosphate triphosphohydrolase SAMHD1**	0.563	0.368	0.862	0.217	0.008135331	0.034581448
**Protein canopy homolog 3**	1.188	1.046	1.35	0.0652	0.008153574	0.034609243
**Dynamin-2**	1.116	1.029	1.21	0.0414	0.008187887	0.034725288
**Interferon alpha-16**	0.676	0.505	0.904	0.148	0.008378261	0.035502434
**Anthrax toxin receptor 1**	0.654	0.477	0.897	0.161	0.008404763	0.035584452
**T-complex protein 1 subunit theta**	1.128	1.031	1.234	0.0458	0.0084822 6	0.035882051
**Peroxisomal carnitine O-octanoyltransferase**	1.166	1.04	1.307	0.0583	0.008554752	0.036157987
**Interleukin-17F**	0.655	0.477	0.898	0.161	0.008582957	0.036246432
**Growth hormone variant**	1.186	1.044	1.347	0.065	0.008763069	0.036975695
**D-glucuronyl C5-epimerase**	0.671	0.498	0.904	0.152	0.008813531	0.037157131
**Carboxypeptidase B2**	0.49	0.288	0.836	0.272	0.008863445	0.037335949
**Cytoplasmic protein NCK2**	1.09	1.022	1.163	0.033	0.008874107	0.037349265
**Kremen protein 1**	1.87	1.17	2.991	0.24	0.008950907	0.037640682
**Tyrosine-protein kinase BTK**	1.089	1.021	1.16	0.0325	0.008961603	0.037653857
**Suppressor of cytokine signaling 3**	0.639	0.457	0.894	0.171	0.008972039	0.03766592
**3-keto-steroid reductase**	1.137	1.032	1.251	0.049	0.008994587	0.037728771

**Neurexin-2-beta**	0.583	0.389	0.874	0.207	0.009055627	0.037952835
**Tubulin-specific chaperone E**	1.097	1.023	1.176	0.0355	0.009071796	0.037988623
**RNA binding protein fox-1 homolog 2**	1.396	1.087	1.794	0.128	0.009081908	0.037999006
**Glutathione reductase, mitochondrial**	0.738	0.587	0.928	0.117	0.009253999	0.038686532
**Leucine-rich repeat transmembrane neuronal protein 4**	0.526	0.324	0.853	0.247	0.009267554	0.038710696
**Small glutamine-rich tetratricopeptide repeat-containing protein alpha**	1.12	1.028	1.219	0.0435	0.009283242	0.038743725
**Nectin-2**	1.634	1.129	2.365	0.189	0.009309549	0.038820974
**Growth/differentiation factor 7**	1.298	1.066	1.579	0.1	0.009323782	0.038847791
**VPS10 domain-containing receptor SorCS1**	1.532	1.11	2.112	0.164	0.009360538	0.038968325
**Angiotensinogen**	0.699	0.534	0.916	0.138	0.009384167	0.039034057
**cGMP-specific 3',5'-cyclic phosphodiesterase**	1.1	1.024	1.182	0.0367	0.009412242	0.039118159
**BRISC complex subunit Abro1**	1.239	1.054	1.456	0.0825	0.009443309	0.039214542
**Vacuolar protein sorting-associated protein 4A**	1.155	1.036	1.288	0.0555	0.00946437	0.039269247
**Arsenite methyltransferase**	0.662	0.485	0.904	0.159	0.00947254	0.03927042
**Myeloid zinc finger 1**	0.668	0.492	0.906	0.156	0.009544851	0.039537283
**Proenkephalin-A**	1.282	1.062	1.548	0.0961	0.009617029	0.03980315
**Bromodomain testis-specific protein**	0.605	0.413	0.885	0.194	0.009626941	0.039811079
**Pro-neuregulin-2, membrane-bound isoform**	1.299	1.065	1.583	0.101	0.009643965	0.039848384
**Glutaredoxin-3**	1.144	1.033	1.267	0.0521	0.009669587	0.039914964
**Phosphatidylinositol transfer protein alpha isoform**	0.52	0.316	0.853	0.253	0.009676112	0.039914964
**G antigen 2A**	1.243	1.054	1.465	0.0841	0.009748269	0.04017933
**Inter-alpha-trypsin inhibitor heavy chain H5**	1.411	1.087	1.831	0.133	0.009772735	0.040246855
**Receptor-type tyrosine-protein phosphatase H**	0.663	0.486	0.906	0.159	0.009785211	0.040264932
**Platelet glycoprotein 4**	0.804	0.681	0.949	0.0846	0.00981546	0.04035605
**TATA-binding protein-associated factor 2N**	1.21	1.047	1.398	0.0737	0.009842638	0.040401068
**EKC/KEOPS complex subunit TPRKB**	0.627	0.44	0.894	0.181	0.009834639	0.040401068
**ADP-ribosylation factor GTPase-activating protein 1**	0.578	0.381	0.877	0.212	0.009874699	0.04049928
**Steroid receptor RNA activator 1**	1.309	1.067	1.606	0.104	0.009887837	0.040519787
**Osteopontin**	1.267	1.058	1.516	0.0917	0.009942058	0.040708475
**Pirin**	1.399	1.084	1.806	0.13	0.010002187	0.040921027
**Ubiquitin-conjugating enzyme E2 A**	0.555	0.354	0.869	0.229	0.01002954	0.040999243
**ATP synthase subunit O, mitochondrial**	1.093	1.021	1.17	0.0347	0.010115333	0.041248356
**Chymase**	0.698	0.531	0.918	0.14	0.010110361	0.041248356
**NAD-dependent protein deacetylase sirtuin-2**	0.786	0.654	0.944	0.0936	0.010106569	0.041248356

**Protein CEI**	0.75	0.602	0.934	0.112	0.010151874	0.041363489
**Interleukin-27 receptor subunit alpha**	1.343	1.073	1.683	0.115	0.010189948	0.04148467
**Tyrosine-protein kinase Fer**	1.08	1.018	1.146	0.0301	0.010234613	0.041616664
**Forkhead box protein L2**	1.243	1.053	1.468	0.0847	0.010239087	0.041616664
**Mitotic spindle assembly checkpoint protein MAD1**	0.622	0.433	0.894	0.185	0.010276749	0.041735673
**Stabilin-1**	0.542	0.339	0.865	0.239	0.01033585	0.041941482
**Nuclear receptor-binding protein**	0.674	0.499	0.911	0.154	0.010355111	0.041985422
**TIR domain-containing adapter molecule 2**	1.24	1.052	1.462	0.084	0.010429369	0.042252098
**Dual specificity tyrosine-phosphorylation-regulated kinase 3**	0.564	0.364	0.874	0.224	0.0104682	0.042374934
**Epithelial discoidin domain-containing receptor 1**	1.298	1.063	1.584	0.102	0.010490758	0.042431748
**E3 ubiquitin-protein ligase RNF13**	0.561	0.36	0.874	0.226	0.010533381	0.042569565
**High mobility group protein B3**	0.719	0.558	0.926	0.129	0.010610772	0.042847552
**Protein S100-A4**	0.723	0.564	0.927	0.127	0.010642484	0.042940783
**PEST proteolytic signal-containing nuclear protein**	1.137	1.03	1.254	0.0502	0.010656814	0.042963787
**cGMP-specific 3',5'-cyclic phosphodiesterase**	1.087	1.02	1.16	0.0328	0.010667326	0.042971372
**Synaptotagmin-like protein 4**	1.078	1.018	1.142	0.0295	0.01069561	0.043050477
**AP-1 complex subunit gamma-like 2**	1.111	1.025	1.204	0.0411	0.010743287	0.043207452
**Hematological and neurological expressed 1 protein**	0.842	0.738	0.961	0.0674	0.010796085	0.043362209
**Copper chaperone for superoxide dismutase**	0.769	0.628	0.941	0.103	0.010799185	0.043362209
**Mimecan**	1.295	1.061	1.58	0.102	0.010853015	0.04354324
**Angiopoietin-related protein 3**	1.43	1.086	1.885	0.141	0.010955862	0.043920482
**Interleukin-13**	0.574	0.374	0.881	0.219	0.011041551	0.044228385
**Membrane cofactor protein**	1.76	1.138	2.723	0.223	0.011068614	0.044270683
**TGF-beta receptor type-2**	0.737	0.583	0.933	0.12	0.011069894	0.044270683
**Leucine-rich repeat-containing protein 59**	1.071	1.016	1.129	0.0269	0.011096287	0.04434062
**Ras-related protein Rab-5B**	1.14	1.03	1.262	0.0517	0.011115708	0.044351371
**Hemoglobin subunit zeta**	0.661	0.481	0.91	0.163	0.011116793	0.044351371
**Exosome complex component RRP43**	1.207	1.044	1.395	0.0741	0.011211533	0.044693532
**Cytochrome b5**	1.257	1.053	1.5	0.0903	0.011289348	0.044967731
**Heterogeneous nuclear ribonucleoprotein A/B**	1.301	1.061	1.594	0.104	0.011299318	0.044971465
**Tumor necrosis factor receptor superfamily member 12A**	1.187	1.04	1.356	0.0678	0.011334495	0.045039466
**Beta-casein**	0.804	0.679	0.952	0.0863	0.011329565	0.045039466
**Pre-mRNA-processing factor 6**	0.653	0.47	0.908	0.168	0.01135293	0.045076745
**Malate dehydrogenase, mitochondrial**	1.11	1.024	1.204	0.0414	0.011486617	0.045571207

**T-cell surface glycoprotein CD5**	1.383	1.075	1.779	0.128	0.011516893	0.045654943
**Lysine--tRNA ligase**	1.118	1.025	1.219	0.0441	0.011531382	0.045676016
**Transmembrane and coiled-coil domains protein 3**	0.795	0.665	0.95	0.0908	0.011564643	0.045771348
**Bis(5'-nucleosyl)-tetraphosphatase [asymmetrical]**	1.105	1.023	1.194	0.0395	0.011610411	0.045915993
**Calcium-binding protein 7**	0.769	0.627	0.943	0.104	0.011655214	0.046056597
**NKG2-E type II integral membrane protein**	1.386	1.075	1.786	0.129	0.011729366	0.046276159
**Glycine amidinotransferase, mitochondrial**	1.125	1.026	1.232	0.0467	0.011729314	0.046276159
**2-phosphoxylose phosphatase 1**	0.581	0.381	0.886	0.216	0.011770511	0.046401723
**Cold-inducible RNA-binding protein**	1.129	1.027	1.241	0.0483	0.011867439	0.04674682
**Heat shock 70 kDa protein 1B**	1.325	1.064	1.649	0.112	0.011913212	0.046890025
**Cadherin-8**	0.786	0.652	0.948	0.0958	0.011988496	0.04714907
**Neurotrimin**	0.65	0.464	0.911	0.172	0.012309833	0.04837463
**Eukaryotic translation initiation factor 3 subunit G**	1.136	1.028	1.256	0.0511	0.012355834	0.048402596
**Toll-interacting protein**	1.1	1.021	1.185	0.038	0.012348544	0.048402596
**BTB/POZ domain-containing protein KCTD5**	0.569	0.366	0.885	0.225	0.012338795	0.048402596
**Neuron-specific vesicular protein calcyon**	1.344	1.066	1.693	0.118	0.012347364	0.048402596
**Chloride channel protein ClC-Kb**	1.15	1.031	1.283	0.0558	0.012420767	0.048618712
**Extracellular superoxide dismutase [Cu-Zn]**	1.268	1.052	1.527	0.095	0.012484808	0.048830996
**Keratin, type II cytoskeletal 7**	0.713	0.546	0.93	0.136	0.012498733	0.048847089
**Ubiquitin thioesterase OTUB2**	0.667	0.486	0.917	0.162	0.012652609	0.049409677
**PIK3CA/PIK3R1**	1.211	1.042	1.407	0.0767	0.012718267	0.049627155
**Acyl-CoA-binding protein**	1.216	1.043	1.419	0.0787	0.012786465	0.049854196
**Pregnancy-specific beta-1-glycoprotein 3**	1.285	1.055	1.566	0.101	0.012822956	0.049957353
**NmrA-like family domain-containing protein 1**	1.173	1.034	1.33	0.0641	0.012841856	0.049991868

Supplemental Figure 1: *Barchart of absolute weights from 140 proteins prioritized by LASSO model. Tetranectin and fibulin-1 model weights were significantly higher than other protein weights in developed LASSO on EXSCEL proteomics cohort.* Barchart of absolute weights from 140 LASSO model proteins. Tetranectin and fibulin-1 (blue) had significantly higher absolute weight values compared to the other heavily ranked proteins that were predictive

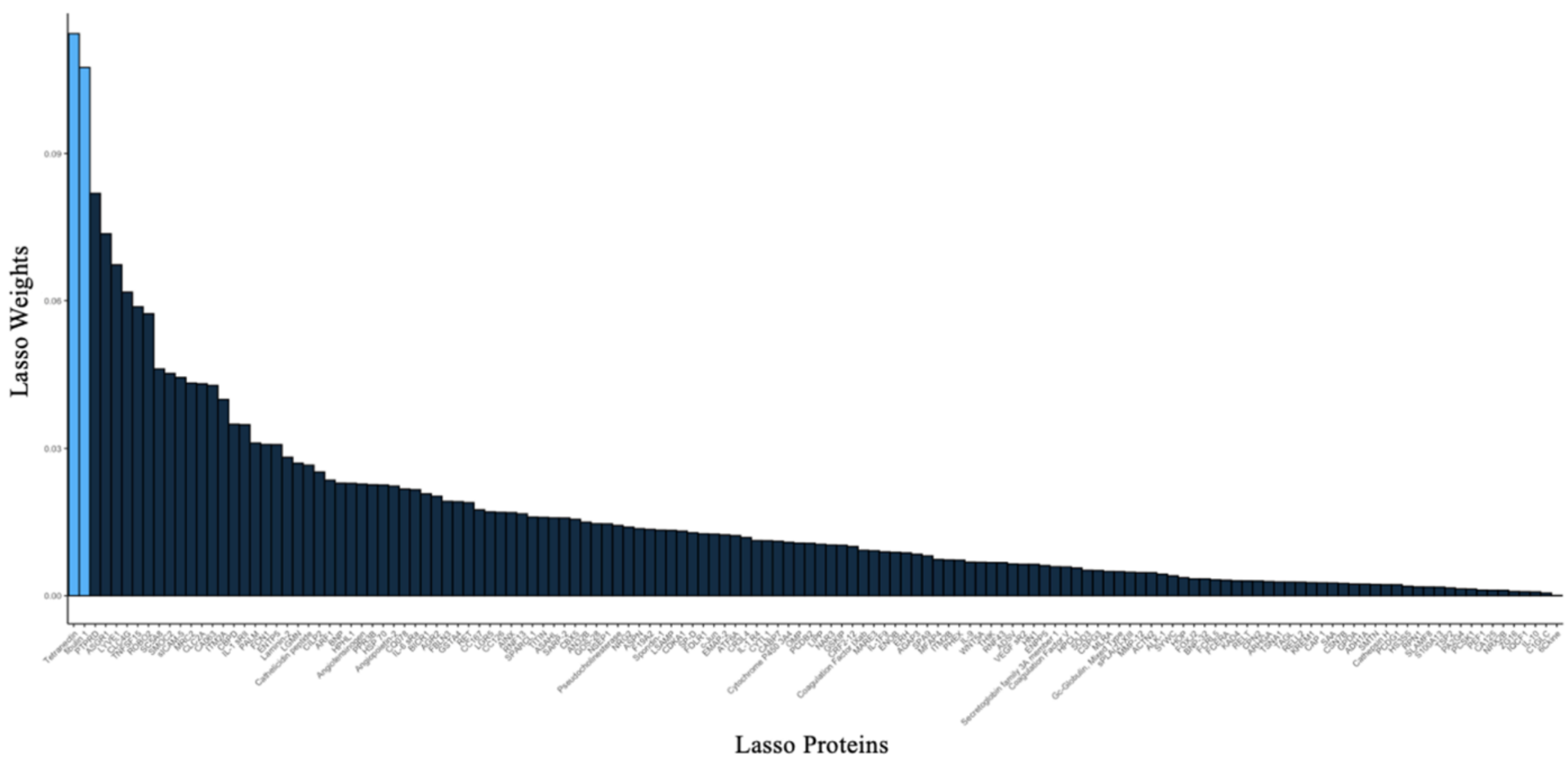

Supplement Table 5: *LASSO model weights of 140 prioritized proteins in EXSCEL proteomics cohort*. These weights were used to calculate the EXSCEL protein score. Bolded proteins are significantly modified by GLP-1 RA treatment at 12 months in linear mixed modeling (p-value <0.05). KEGG pathway annotation represents curated annotations based on established gene/protein function. Formal statistical pathway enrichment analysis was underpowered at this set size and did not achieve FDR-corrected significance.

Protein	LASSO Coefficient	GLP-1 RA Modified	Coefficient Direction	KEGG Pathway Annotation
**Tetranectin**	**-0.1144036**	**Yes**	**Protective**	**hsa04512: ECM-receptor interaction**
Receptor-type tyrosine-protein phosphatase delta	-0.0819269	No	Protective	hsa04014: Ras signaling pathway
**Lymphatic vessel endothelial hyaluronic acid receptor 1**	**-0.0673018**	**Yes**	**Protective**	**hsa04151: PI3K-Akt signaling pathway**
Carboxypeptidase D	-0.0349034	No	Protective	hsa04614: Renin-angiotensin system
**Interleukin-1 receptor type 2**	**-0.0348093**	**Yes**	**Protective**	**hsa04060: Cytokine-cytokine receptor interaction; hsa04657: IL-17 signaling pathway**

**Ectonucleoside triphosphate diphosphohydrolase 5**	**-0.0307437**	**Yes**	**Protective**	**hsa04141: Protein processing in endoplasmic reticulum; hsa00230: Purine metabolism**
Legumain	-0.0269726	No	Protective	hsa04142: Lysosome
**Cathelicidin antimicrobial peptide**	**-0.0265670**	**Yes**	**Protective**	**hsa04620: Toll-like receptor signaling pathway; hsa04062: Chemokine signaling pathway**
Cartilage intermediate layer protein 2	-0.0251522	No	Protective	hsa04512: ECM-receptor interaction
Hephaestin-like protein 1	-0.0228667	No	Protective	hsa04216: Ferroptosis
Angiotensinogen	-0.0227303	No	Protective	hsa04614: Renin-angiotensin system
Protein phosphatase 1 regulatory subunit 3B	-0.0225943	No	Protective	hsa04910: Insulin signaling pathway; hsa00500: Starch and sucrose metabolism
**Heat shock 70 kDa protein 1A**	**-0.0225457**	**Yes**	**Protective**	**hsa04141: Protein processing in endoplasmic reticulum; hsa04010: MAPK signaling pathway**
Uncharacterized protein C17orf78	-0.0216699	No	Protective	Not annotated in KEGG
Interleukin-6 receptor subunit alpha	-0.0215328	No	Protective	hsa04060: Cytokine-cytokine receptor interaction; hsa04630: JAK-STAT signaling pathway
Anterior gradient protein 2 homolog	-0.0202313	No	Protective	hsa04141: Protein processing in endoplasmic reticulum
**Proto-oncogene tyrosine-protein kinase receptor Ret**	**-0.0189000**	**Yes**	**Protective**	**hsa04010: MAPK signaling pathway; hsa04151: PI3K-Akt signaling pathway; hsa05200: Pathways in cancer**
Coiled-coil domain-containing protein 167	-0.0174728	No	Protective	Not annotated in KEGG
Leucine-rich repeat-containing G-protein coupled receptor 5	-0.0170453	No	Protective	hsa04310: Wnt signaling pathway
Coiled-coil domain-containing protein 126	-0.0169396	No	Protective	Not annotated in KEGG
E3 ubiquitin-protein ligase RNF13	-0.0166299	No	Protective	hsa04120: Ubiquitin mediated proteolysis
SPARC-like protein 1	-0.0159788	No	Protective	hsa04512: ECM-receptor interaction
**N-acylethanolamine-hydrolyzing acid amidase**	**-0.0158083**	**Yes**	**Protective**	**hsa00591: Linoleic acid metabolism; hsa04726: Serotonergic synapse**
**Cholinesterase**	**-0.0142508**	**Yes**	**Protective**	**hsa00564: Glycerophospholipid metabolism;**

				**hsa04725: Cholinergic synapse**
**Asporin**	**-0.0136156**	**Yes**	**Protective**	**hsa04512: ECM-receptor interaction; hsa04350: TGF-beta signaling pathway**
Protein FAM19A2	-0.0134619	No	Protective	Not annotated in KEGG
Endothelial monocyte-activating polypeptide 2	-0.0123383	No	Protective	hsa04141: Protein processing in endoplasmic reticulum
**Cyclic AMP-dependent transcription factor ATF-6 alpha**	**-0.0121858**	**Yes**	**Protective**	**hsa04141: Protein processing in endoplasmic reticulum; hsa04932: Non-alcoholic fatty liver disease**
Calcium-binding protein 7	-0.0110565	No	Protective	hsa04020: Calcium signaling pathway
Cytochrome P450 3A4	-0.0108413	No	Protective	hsa00140: Steroid hormone biosynthesis; hsa00590: Arachidonic acid metabolism
Olfactory marker protein	-0.0106942	No	Protective	Not annotated in KEGG
Protocadherin beta-2	-0.0106625	No	Protective	hsa04514: Cell adhesion molecules
**Pancreatic triacylglycerol lipase**	**-0.0104663**	**Yes**	**Protective**	**hsa00561: Glycerolipid metabolism; hsa04972: Pancreatic secretion**
**Ecto-ADP-ribosyltransferase 3**	**-0.0102957**	**Yes**	**Protective**	**Not annotated in KEGG**
Coagulation factor IXab	-0.0092280	No	Protective	hsa04610: Complement and coagulation cascades
**Beta-enolase**	**-0.0088046**	**Yes**	**Protective**	**hsa00010: Glycolysis / Gluconeogenesis; hsa01200: Carbon metabolism**
Arf-GAP with GTPase, ANK repeat and PH domain-containing protein 3	-0.0084468	No	Protective	hsa04144: Endocytosis
3-ketoacyl-CoA thiolase, peroxisomal	-0.0067187	No	Protective	hsa00071: Fatty acid degradation; hsa00280: Valine, leucine and isoleucine degradation
E3 ubiquitin-protein ligase RNF43	-0.0067051	No	Protective	hsa04310: Wnt signaling pathway; hsa04120: Ubiquitin mediated proteolysis
Vascular endothelial growth factor receptor 2	-0.0063902	No	Protective	hsa04370: VEGF signaling pathway; hsa04066: HIF-1 signaling pathway
**Hematological and neurological expressed 1 protein**	**-0.0063893**	**Yes**	**Protective**	**Not annotated in KEGG**
Ectonucleotide pyrophosphatase/phospho	-0.0061387	No	Protective	hsa00230: Purine metabolism

diesterase family member 5				
Coagulation Factor V	-0.0058480	No	Protective	hsa04610: Complement and coagulation cascades
Hippocalcin-like protein 1	-0.0056220	No	Protective	hsa04020: Calcium signaling pathway
Neurocan core protein	-0.0051238	No	Protective	hsa04514: Cell adhesion molecules; hsa05205: Proteoglycans in cancer
Myosin regulatory light chain 2, atrial isoform	-0.0049108	No	Protective	hsa04260: Cardiac muscle contraction; hsa05410: Hypertrophic cardiomyopathy
**Vitamin D-binding protein**	**-0.0048845**	**Yes**	**Protective**	**hsa04978: Mineral absorption; hsa04961: Endocrine and other factor-regulated calcium reabsorption**
**Group XIIB secretory phospholipase A2-like protein**	**-0.0047474**	**Yes**	**Protective**	**hsa00564: Glycerophospholipid metabolism; hsa04726: Serotonergic synapse**
Homeodomain-only protein	-0.0036615	No	Protective	Not annotated in KEGG
Tumor necrosis factor receptor superfamily member 19L	-0.0030058	No	Protective	hsa04060: Cytokine-cytokine receptor interaction
Carboxypeptidase N subunit 2	-0.0029891	No	Protective	hsa04610: Complement and coagulation cascades
**Rho guanine nucleotide exchange factor 10**	**-0.0028605**	**Yes**	**Protective**	**hsa04014: Ras signaling pathway; hsa04810: Regulation of actin cytoskeleton**
t-SNARE domain-containing protein 1	-0.0027782	No	Protective	hsa04130: SNARE interactions in vesicular transport
RELT-like protein 2	-0.0027351	No	Protective	hsa04060: Cytokine-cytokine receptor interaction
COP9 signalosome complex subunit 7b	-0.0024822	No	Protective	hsa04120: Ubiquitin mediated proteolysis
Glycerol-3-phosphate dehydrogenase [NAD(+)], cytoplasmic	-0.0023579	No	Protective	hsa00561: Glycerolipid metabolism; hsa00010: Glycolysis / Gluconeogenesis
**Alcohol dehydrogenase 1A**	**-0.0023449**	**Yes**	**Protective**	**hsa00830: Retinol metabolism; hsa00010: Glycolysis / Gluconeogenesis**
Heparan sulfate glucosamine 3-O-sulfotransferase 5	-0.0019362	No	Protective	hsa00534: Glycosaminoglycan biosynthesis — heparan sulfate / heparin
Neurexin-2-beta	-0.0010892	No	Protective	hsa04514: Cell adhesion molecules; hsa04723: Retrograde

				endocannabinoid signaling
**IQ domain-containing protein F1**	**-0.0008098**	**Yes**	**Protective**	**Not annotated in KEGG**
**C1GALT1-specific chaperone 1**	**-0.0005396**	**Yes**	**Protective**	**hsa00512: Mucin type O-glycan biosynthesis**
C-C motif chemokine 21	-0.0000495	No	Protective	hsa04060: Cytokine-cytokine receptor interaction; hsa04062: Chemokine signaling pathway
Interleukin-10	0.0007721	No	Risk-associated	hsa04060: Cytokine-cytokine receptor interaction; hsa04630: JAK-STAT signaling pathway
**Zymogen granule membrane protein 16**	**0.0008521**	**Yes**	**Risk-associated**	**Not annotated in KEGG**
**Mucin-16**	**0.0010985**	**Yes**	**Risk-associated**	**hsa05205: Proteoglycans in cancer**
**Peflin**	**0.0011325**	**Yes**	**Risk-associated**	**Not annotated in KEGG**
ProSAAS	0.0013540	No	Risk-associated	hsa04024: cAMP signaling pathway
Proliferation-associated protein 2G4	0.0013894	No	Risk-associated	hsa03008: Ribosome biogenesis in eukaryotes
**Thrombospondin-2**	**0.0015784**	**Yes**	**Risk-associated**	**hsa04512: ECM-receptor interaction; hsa04350: TGF-beta signaling pathway; hsa04151: PI3K-Akt signaling pathway**
**Protein S100-A13**	**0.0017507**	**Yes**	**Risk-associated**	**hsa04020: Calcium signaling pathway; hsa04010: MAPK signaling pathway**
SLAM family member 8	0.0017739	No	Risk-associated	hsa04650: Natural killer cell mediated cytotoxicity
Dolichyl-diphosphooligosaccharide--protein glycosyltransferase subunit 1	0.0017899	No	Risk-associated	hsa00510: N-Glycan biosynthesis; hsa04141: Protein processing in endoplasmic reticulum
**Protocadherin gamma-A1**	**0.0022281**	**Yes**	**Risk-associated**	**hsa04514: Cell adhesion molecules**
Cathepsin H	0.0022339	No	Risk-associated	hsa04142: Lysosome
Smoothelin	0.0022696	No	Risk-associated	hsa04270: Vascular smooth muscle contraction
Serum amyloid A-1 protein	0.0025752	No	Risk-associated	hsa04610: Complement and coagulation cascades; hsa04060: Cytokine-cytokine receptor interaction
Adenylyl cyclase-associated protein 1	0.0026071	No	Risk-associated	hsa04810: Regulation of actin cytoskeleton

**Kremen protein 1**	**0.0026340**	**Yes**	**Risk-associated**	**hsa04310: Wnt signaling pathway**
Transgelin	0.0027647	No	Risk-associated	hsa04270: Vascular smooth muscle contraction
Adenylate kinase 4, mitochondrial	0.0030256	No	Risk-associated	hsa00230: Purine metabolism
High affinity immunoglobulin epsilon receptor subunit alpha	0.0031375	No	Risk-associated	hsa04664: Fc epsilon RI signaling pathway
Fc receptor-like protein 5	0.0032252	No	Risk-associated	hsa04662: B cell receptor signaling pathway
Brain natriuretic peptide 32	0.0034024	No	Risk-associated	hsa04022: cGMP-PKG signaling pathway; hsa04924: Renin secretion
**Forkhead box protein J2**	**0.0034035**	**Yes**	**Risk-associated**	**Not annotated in KEGG**
Tryptophan--tRNA ligase, cytoplasmic	0.0040519	No	Risk-associated	hsa00970: Aminoacyl-tRNA biosynthesis
Serine/threonine-protein kinase receptor R3 (ALK1/ACVRL1)	0.0043671	No	Risk-associated	hsa04350: TGF-beta signaling pathway; hsa04390: Hippo signaling pathway
**Alpha-actinin-2**	**0.0046523**	**Yes**	**Risk-associated**	**hsa04260: Cardiac muscle contraction; hsa04512: ECM-receptor interaction; hsa05410: Hypertrophic cardiomyopathy**
Macrophage metalloelastase (MMP-12)	0.0046596	No	Risk-associated	hsa04657: IL-17 signaling pathway; hsa04668: TNF signaling pathway; hsa05205: Proteoglycans in cancer
**Extracellular superoxide dismutase [Cu-Zn]**	**0.0051657**	**Yes**	**Risk-associated**	**hsa04146: Peroxisome; hsa04066: HIF-1 signaling pathway**
**Secretoglobin family 3A member 1**	**0.0058897**	**Yes**	**Risk-associated**	**Not annotated in KEGG**
Adseverin	0.0064489	No	Risk-associated	hsa04810: Regulation of actin cytoskeleton
**Protein Wnt-5a**	**0.0067667**	**Yes**	**Risk-associated**	**hsa04310: Wnt signaling pathway; hsa04550: Signaling pathways regulating pluripotency of stem cells**
Interleukin-9	0.0067941	No	Risk-associated	hsa04060: Cytokine-cytokine receptor interaction; hsa04630: JAK-STAT signaling pathway
Phosphate-regulating neutral endopeptidase	0.0072363	No	Risk-associated	hsa04978: Mineral absorption

Integral membrane protein 2B	0.0072751	No	Risk-associated	Not annotated in KEGG
**Microfibril-associated glycoprotein 4**	**0.0073704**	**Yes**	**Risk-associated**	**hsa04610: Complement and coagulation cascades; hsa04512: ECM-receptor interaction**
Serpin A9	0.0080908	No	Risk-associated	hsa04610: Complement and coagulation cascades
Enhancer of rudimentary homolog	0.0087087	No	Risk-associated	Not annotated in KEGG
Interleukin-36 gamma	0.0088890	No	Risk-associated	hsa04060: Cytokine-cytokine receptor interaction; hsa04621: NOD-like receptor signaling pathway
Microtubule-associated protein RP/EB family member 3	0.0091291	No	Risk-associated	hsa04810: Regulation of actin cytoskeleton
**Interferon lambda receptor 1**	**0.0099568**	**Yes**	**Risk-associated**	**hsa04060: Cytokine-cytokine receptor interaction; hsa04630: JAK-STAT signaling pathway; hsa04620: Toll-like receptor signaling pathway**
GSK3-beta interaction protein	0.0102466	No	Risk-associated	hsa04310: Wnt signaling pathway
Cytokine-like protein 1	0.0111680	No	Risk-associated	Not annotated in KEGG
**Interleukin-1 receptor-like 1 (ST2)**	**0.0111952**	**Yes**	**Risk-associated**	**hsa04060: Cytokine-cytokine receptor interaction; hsa04657: IL-17 signaling pathway; hsa04010: MAPK signaling pathway**
Cyclic AMP-responsive element-binding protein 3-like protein 4	0.0118293	No	Risk-associated	hsa04141: Protein processing in endoplasmic reticulum; hsa04010: MAPK signaling pathway
Transcription factor AP-1	0.0125082	No	Risk-associated	hsa04010: MAPK signaling pathway; hsa04668: TNF signaling pathway; hsa04620: Toll-like receptor signaling pathway
Folate receptor alpha	0.0125792	No	Risk-associated	hsa00790: Folate biosynthesis; hsa04977: Vitamin digestion and absorption
**Pulmonary surfactant-associated protein D**	**0.0128089**	**Yes**	**Risk-associated**	**hsa04610: Complement and coagulation cascades; hsa04620: Toll-like receptor signaling pathway**

Cyclin-dependent kinase 2-associated protein 1	0.0131103	No	Risk-associated	hsa04110: Cell cycle
Limbic system-associated membrane protein	0.0132564	No	Risk-associated	hsa04514: Cell adhesion molecules
Spondin-1	0.0133010	No	Risk-associated	hsa04512: ECM-receptor interaction
Pro-neuregulin-2, membrane-bound isoform	0.0139333	No	Risk-associated	hsa04012: ErbB signaling pathway; hsa04010: MAPK signaling pathway
Nuclease-sensitive element-binding protein 1	0.0145572	No	Risk-associated	hsa03013: RNA transport
Golgi SNAP receptor complex member 1	0.0145995	No	Risk-associated	hsa04130: SNARE interactions in vesicular transport
**Acidic leucine-rich nuclear phosphoprotein 32 family member B**	**0.0149600**	**Yes**	**Risk-associated**	**hsa04210: Apoptosis**
Chromobox protein homolog 5	0.0155012	No	Risk-associated	Not annotated in KEGG
Secreted frizzled-related protein 1	0.0157873	No	Risk-associated	hsa04310: Wnt signaling pathway
Titin	0.0159108	No	Risk-associated	hsa04260: Cardiac muscle contraction; hsa04512: ECM-receptor interaction; hsa05410: Hypertrophic cardiomyopathy
Alpha-internexin	0.0168776	No	Risk-associated	Not annotated in KEGG
Glutathione S-transferase A4	0.0190983	No	Risk-associated	hsa00480: Glutathione metabolism; hsa04146: Peroxisome
**EGF-containing fibulin-like extracellular matrix protein 1**	**0.0191681**	**Yes**	**Risk-associated**	**hsa04512: ECM-receptor interaction; hsa04151: PI3K-Akt signaling pathway**
Bicaudal D-related protein 1	0.0207053	No	Risk-associated	Not annotated in KEGG
**Angiopoietin-2**	**0.0223188**	**Yes**	**Risk-associated**	**hsa04151: PI3K-Akt signaling pathway; hsa04014: Ras signaling pathway; hsa04066: HIF-1 signaling pathway**
Natriuretic peptides B	0.0228994	No	Risk-associated	hsa04022: cGMP-PKG signaling pathway; hsa04924: Renin secretion
**ADP-ribosylation factor 1**	**0.0235128**	**Yes**	**Risk-associated**	**hsa04144: Endocytosis; hsa04130: SNARE interactions in vesicular transport**
**Laminin-2**	**0.0281958**	**Yes**	**Risk-associated**	**hsa04512: ECM-receptor interaction; hsa04151: PI3K-Akt signaling pathway; hsa04510: Focal adhesion**

**Ficolin-1**	**0.0307750**	**Yes**	**Risk-associated**	**hsa04610: Complement and coagulation cascades**
Paralemmin-1	0.0311002	No	Risk-associated	Not annotated in KEGG
**Integral membrane protein 2A**	**0.0399496**	**Yes**	**Risk-associated**	**Not annotated in KEGG**
Arginine/serine-rich protein 1	0.0428071	No	Risk-associated	Not annotated in KEGG
C-type lectin domain family 2 member A	0.0431430	No	Risk-associated	hsa04650: Natural killer cell mediated cytotoxicity
**C-type mannose receptor 2**	**0.0432888**	**Yes**	**Risk-associated**	**hsa04145: Phagosome; hsa05152: Tuberculosis**
**Intercellular adhesion molecule 5**	**0.0444052**	**Yes**	**Risk-associated**	**hsa04514: Cell adhesion molecules; hsa04510: Focal adhesion**
SPARC-related modular calcium-binding protein 2	0.0452497	No	Risk-associated	hsa04512: ECM-receptor interaction
Sodium-coupled monocarboxylate transporter 1	0.0461607	No	Risk-associated	hsa04978: Mineral absorption
**Roundabout homolog 2**	**0.0573680**	**Yes**	**Risk-associated**	**hsa04360: Axon guidance**
Tumor necrosis factor ligand superfamily member 15	0.0587732	No	Risk-associated	hsa04060: Cytokine-cytokine receptor interaction; hsa04668: TNF signaling pathway
C-type lectin domain family 4 member G	0.0617585	No	Risk-associated	hsa04650: Natural killer cell mediated cytotoxicity
Asialoglycoprotein receptor 1	0.0736696	No	Risk-associated	hsa04144: Endocytosis
Fibulin-1	0.1074668	No	Risk-associated	hsa04512: ECM-receptor interaction; hsa04151: PI3K-Akt signaling pathway

Supplemental Table 6: *Pairwise bootstrap comparisons of ΔAUPRC among proteomic machine-learning models in the EXSCEL held-out test set.* Pairwise differences in area under the precision-recall curve (ΔAUPRC) are shown for LASSO, elastic net, gradient boosted trees, and random forest models. Values are reported as ΔAUPRC with 95% confidence intervals and paired bootstrap p-values derived from stratified nonparametric bootstrap resampling of the held-out test set.

Model comparison	ΔAUPRC (95% CI)	p-value
LASSO vs Elastic Net	0.020 (-0.002, 0.043)	0.074
LASSO vs Gradient Boosted Trees	0.040 (-0.002, 0.081)	0.063
LASSO vs Random Forest	0.089 (0.043, 0.140)	<0.001
Elastic Net vs Gradient Boosted Trees	0.020 (-0.015, 0.052)	0.233
Elastic Net vs Random Forest	0.069 (0.028, 0.113)	<0.001
Gradient Boosted Trees vs Random Forest	0.048 (0.011, 0.092)	0.010

Supplemental Table 7: *Pairwise bootstrap comparisons of ΔAUPRC among clinical and proteomic prediction models in the EXSCEL held-out test set.* Pairwise differences in area under the precision-recall curve (ΔAUPRC) are shown for the clinical-only, protein-only, protein + clinical, and clinical + tetranectin + fibulin-1 models. Values are reported as ΔAUPRC with 95% confidence intervals and paired bootstrap p-values derived from stratified nonparametric bootstrap resampling of the held-out test set.

Model comparison	ΔAUPRC (95% CI)	p-value
Protein only vs Clinical only	0.158 (0.103, 0.214)	<0.001
Protein + Clinical vs Clinical only	0.206 (0.144, 0.265)	<0.001
Clinical + tetranectin + fibulin-1 vs Clinical only	0.044 (0.007, 0.082)	0.017
Protein + Clinical vs Protein only	0.048 (0.022, 0.073)	<0.001
Clinical + tetranectin + fibulin-1 vs Protein only	-0.113 (-0.170, -0.061)	<0.001
Protein + Clinical vs Clinical + tetranectin + fibulin-1	0.162 (0.105, 0.218)	<0.001

Model comparison	ΔAUPRC (95% CI)	p-value
Protein only vs Clinical only	0.158 (0.103, 0.214)	<0.001
Protein + Clinical vs Clinical only	0.206 (0.144, 0.265)	<0.001
Clinical + Tetranectin + Fibulin-1 vs Clinical only	0.044 (0.007, 0.082)	0.017
Protein + Clinical vs Protein only	0.048 (0.022, 0.073)	<0.001
Clinical + Tetranectin + Fibulin-1 vs Protein only	-0.113 (-0.170, -0.061)	<0.001
Protein + Clinical vs Clinical + Tetranectin + Fibulin-1	0.162 (0.105, 0.218)	<0.001

Supplement Figure 2: *Boxplot of tetranectin levels stratified by MACE and history of ASCVD in EXSCEL proteomics cohort.* ANOVA testing of mean tetranectin levels in MACE+ individuals +/- history of ASCVD had significantly lower levels of circulating baseline tetranectin compared to MACE- individuals, p = 0.038. ASCVD+/MACE+ individuals had the lowest mean levels of

circulating tetranectin compared to all other groups. *Significant p-value <0.05

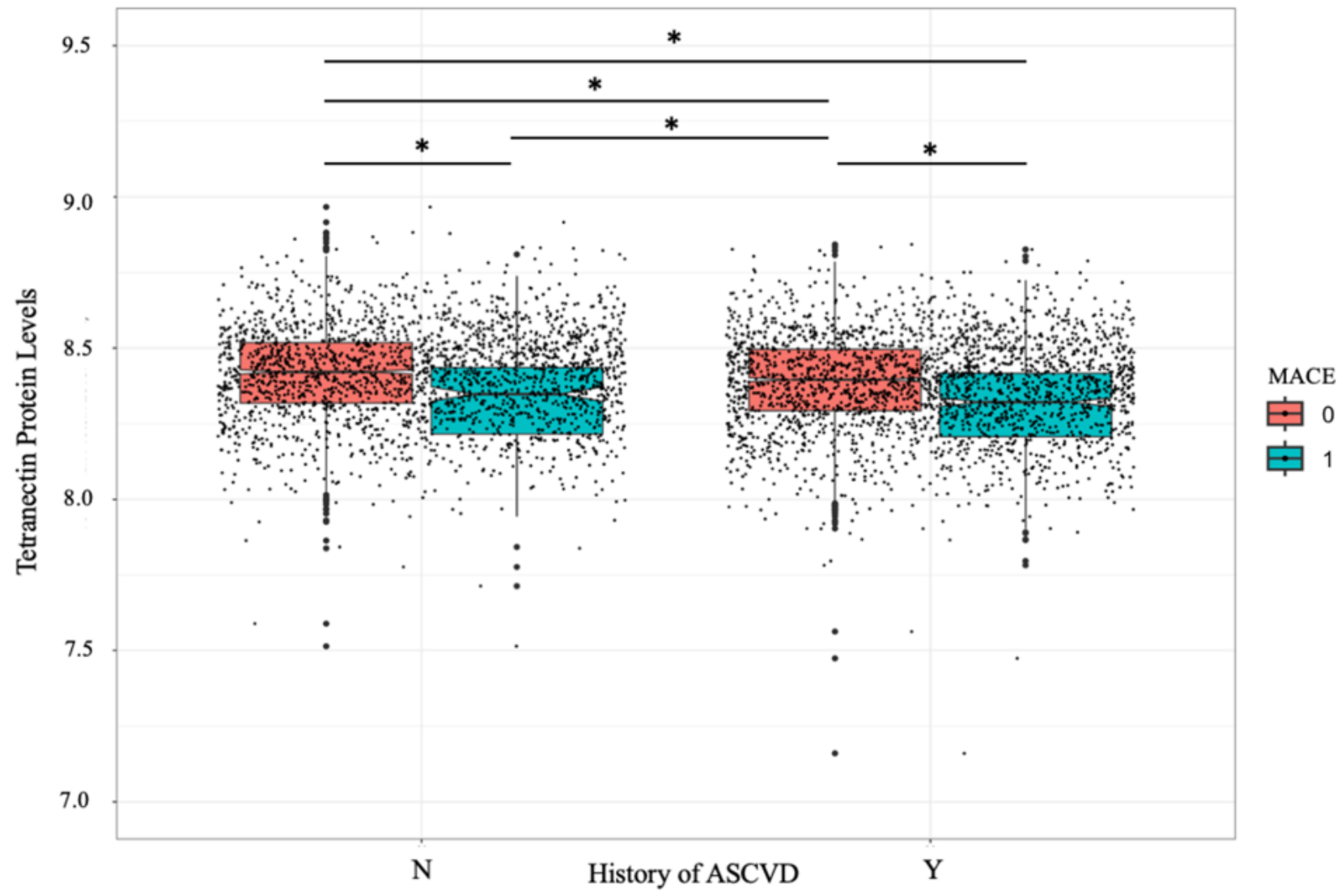

Supplement Table 8: *Post-hoc Tukey honestly significant difference results of baseline tetranectin levels (relative quantification) in cohorts with and without MACE and history of ASCVD in EXSCEL proteomics cohort.* Post-hoc testing demonstrated significantly lower levels of baseline tetranectin levels in those with MACE and a history of ASCVD. *Significant p-value <0.05

Group 1	Group 2	Estimate	95% CI Lower Bound	95% CI Upper Bound	FDR adjusted p-value
ASCVD+/MACE+	ASCVD-/MACE +	0.019	-0.015	0.053	0.49
ASCVD+/MACE+	ASCVD+/MACE-	0.073	0.052	0.093	$4.6e^{-9}$ *
ASCVD+/MACE+	ASCVD-/MACE-	0.10	0.081	0.12	$4.6e^{-9}$ *
ASCVD–/MACE+	ASCVD+/MACE-	0.054	0.024	0.084	$2.7e^{-5}$ *
ASCVD–/MACE+	ASCVD-/MACE-	0.083	0.053	0.11	$4.4e^{-9}$ *
ASCVD+/MACE-	ASCVD-/MACE-	0.029	0.016	0.042	$5.2e^{-8}$ *

Supplement Figure 3: *Boxplot of fibulin-1 levels stratified by MACE and history of ASCVD in EXSCEL proteomics cohort.* MACE+ cohorts +/- history of ASCVD have significantly higher levels of circulating baseline fibulin-1 compared to MACE- individuals, p = 0.029. ASCVD+/MACE+ individuals have the highest mean levels of circulating fibulin-1 compared to all other groups. *Significant p-value <0.05

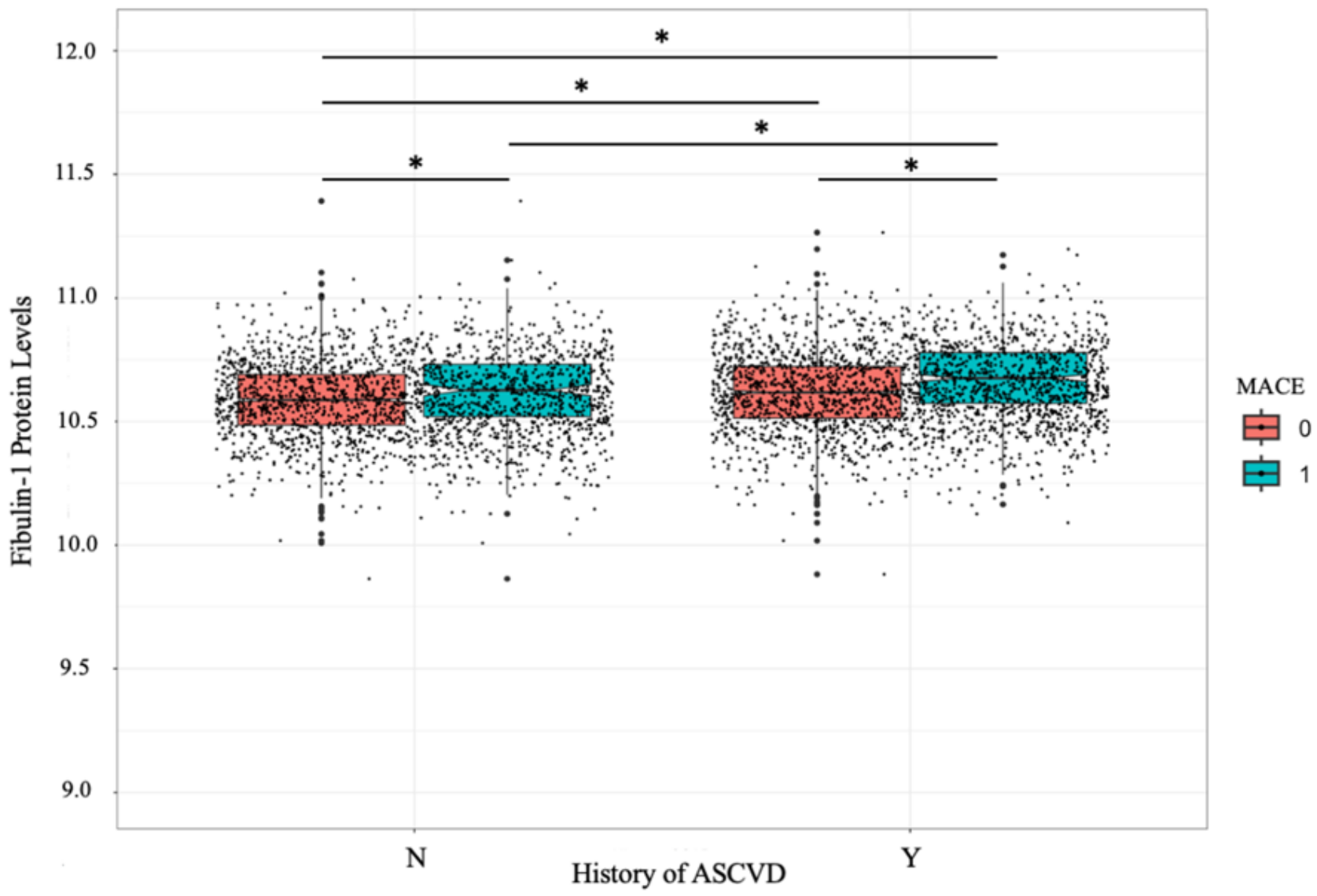

Supplement Table 9: *Post-hoc Tukey honestly significant difference results of baseline fibulin-1 levels (relative quantification) in history of ASCVD and MACE subsets in EXSCEL proteomics cohort.* Post-hoc testing demonstrated significantly higher levels of baseline fibulin-1 levels in people with MACE and history of ASCVD. *Significant p-value <0.05

Group 1	Group 2	Estimate	95% CI Lower Bound	95% CI Upper Bound	FDR adjusted p-value
ASCVD+/MACE+	ASCVD-/MACE +	-0.047	-0.080	-0.014	0.0015*
ASCVD+/MACE+	ASCVD+/MACE-	-0.060	-0.080	-0.040	$4.6e^{-9}$ *
ASCVD+/MACE+	ASCVD-/MACE-	-0.090	-0.11	-0.070	$4.6e^{-9}$ *
ASCVD–/MACE+	ASCVD+/MACE-	-0.013	-0.042	0.017	0.68
ASCVD–/MACE+	ASCVD-/MACE-	-0.043	-0.072	-0.013	0.0012 *
ASCVD+/MACE-	ASCVD-/MACE-	-0.030	-0.042	-0.017	$1.2e^{-8}$ *

Supplement Figure 4: *Boxplots of tetranectin and fibulin-1 levels at baseline and 12-months stratified by treatment arm in the EXSCEL proteomics cohort.* Visual differences in raw protein levels over time were modest. However, linear mixed-effect modeling demonstrated a significant treatment-associated change over time for tetranectin ($p < 0.05$), whereas fibulin-1 did not show significant modification by GLP-1 RA treatment.

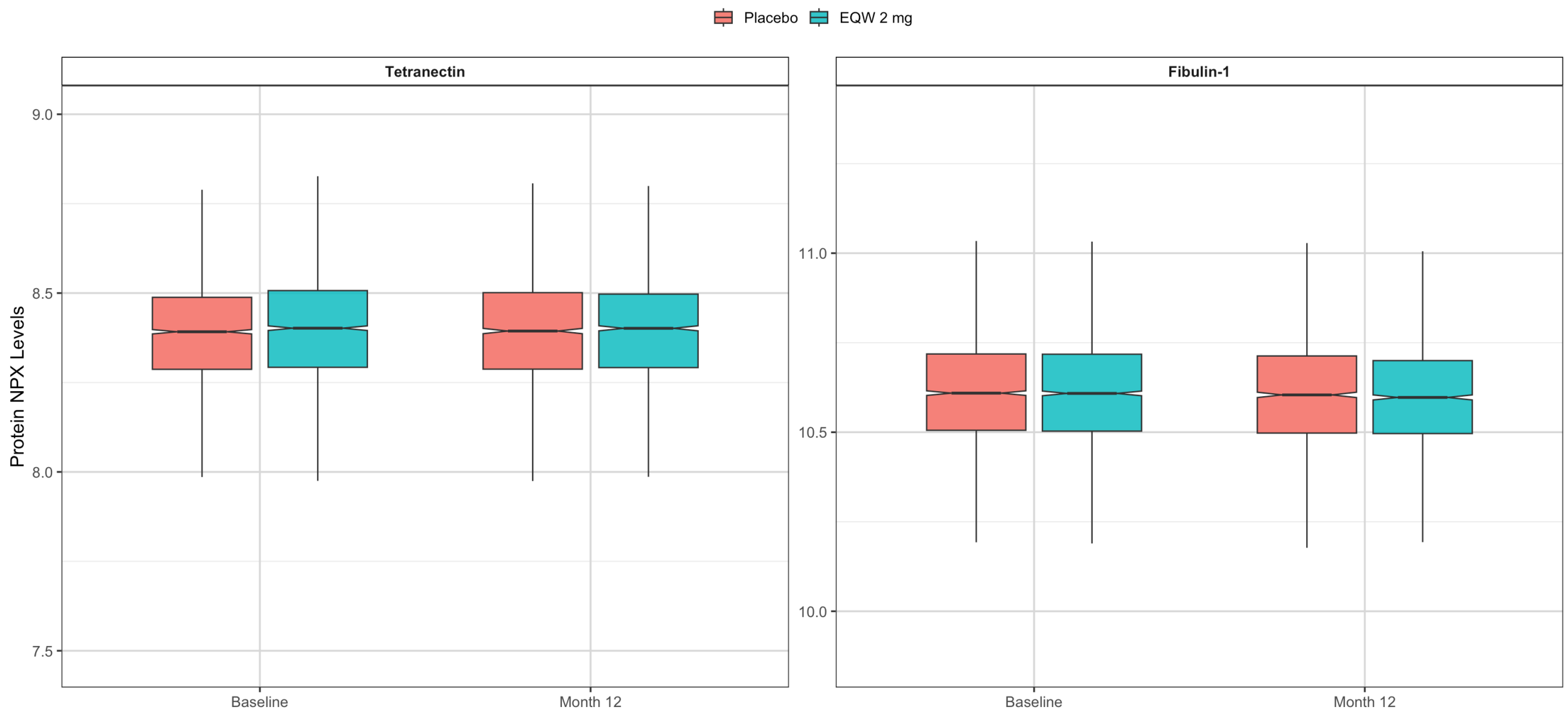

Supplemental Table 10: *Mean change in log-transformed protein NPX values from baseline to Month 12 by treatment arm in the EXSCEL proteomics cohort.* Values are shown as mean change (Month 12 − baseline) with standard deviation. Negative values reflect decreasing protein levels over time and positive values reflect increasing levels. Tetranectin demonstrated a modest directional difference between treatment arms, with a mean decrease in the GLP-1 RA group and a mean increase in the placebo group, whereas fibulin-1 showed similarly small mean decreases in both groups.

	Placebo (Mean Change, SD)	GLP-1 RA (Mean Change, SD)
Tetranectin	+0.00676 (0.129)	-0.00628 (0.134)
Fibulin-1	-0.0132 (0.144)	-0.0114 (0.145)

Supplement Table 11: *Causal mediation analysis of weight loss for GLP-1 RA–modified proteins*. Displayed are the average causal mediation effect (ACME), average direct effect (ADE), total effect (TE), proportion mediated for each protein, with 95% confidence intervals and corresponding p-values. Significant ACME values indicate proteins for which part of the treatment-associated change is mediated by weight loss from baseline to 12	ACME estimate (95% CI)	ACME p-value	ADE estimate (95% CI)	ADE p-value	TE estimate (95% CI)	TE p-value	Proportion mediated (95% CI)	Proportion mediated p-value

months. The proportion mediated represents the estimated fraction of the total treatment effect attributable to weight loss.Protein							
Kremen protein 1	-0.011 (-0.019, -0.005)	<0.001	-0.165 (-0.213, -0.119)	<0.001	-0.176 (-0.225, -0.131)	<0.001	0.064 (0.026, 0.115)	<0.001
Roundabout homolog 2	-0.010 (-0.016, -0.005)	<0.001	-0.113 (-0.155, -0.072)	<0.001	-0.123 (-0.165, -0.084)	<0.001	0.084 (0.040, 0.153)	<0.001
C-type mannose receptor 2	-0.018 (-0.027, -0.011)	<0.001	-0.188 (-0.236, -0.143)	<0.001	-0.206 (-0.253, -0.159)	<0.001	0.085 (0.050, 0.131)	<0.001
Pulmonary surfactant-associated protein D	-0.007 (-0.011, -0.002)	<0.001	-0.058 (-0.091, -0.025)	<0.001	-0.065 (-0.097, -0.032)	<0.001	0.103 (0.036, 0.230)	<0.001
Extracellular superoxide dismutase [Cu-Zn]	0.011 (0.006, 0.017)	<0.001	0.083 (0.040, 0.122)	<0.001	0.094 (0.050, 0.134)	<0.001	0.112 (0.056, 0.234)	<0.001
N-acylethanolamine-hydrolyzing acid amidase	0.009 (0.004, 0.014)	<0.001	0.069 (0.028, 0.107)	<0.001	0.077 (0.038, 0.114)	<0.001	0.113 (0.044, 0.252)	<0.001
Laminin-2	-0.0	<0.	-0.1	<0.	-0.1	<0	0.117	<0.001

	14 (-0.021, -0.008)	001	07 (-0.145, -0.069)	001	22 (-0.160, -0.084)	.001	(0.063, 0.197)	
Peflin	0.012 (0.006, 0.019)	<0.001	0.089 (0.046, 0.136)	<0.001	0.101 (0.058, 0.146)	<0.001	0.119 (0.052, 0.241)	<0.001
Thrombospondin-2	-0.014 (-0.021, -0.008)	<0.001	-0.096 (-0.138, -0.053)	<0.001	-0.110 (-0.153, -0.068)	<0.001	0.126 (0.065, 0.244)	<0.001
Microfibril-associated glycoprotein 4	-0.016 (-0.024, -0.010)	<0.001	-0.109 (-0.153, -0.065)	<0.001	-0.125 (-0.169, -0.082)	<0.001	0.130 (0.074, 0.224)	<0.001
Interleukin-1 receptor type 2	-0.018 (-0.025, -0.012)	<0.001	-0.116 (-0.153, -0.083)	<0.001	-0.134 (-0.171, -0.102)	<0.001	0.135 (0.087, 0.210)	<0.001
Heat shock 70 kDa protein 1A	-0.015 (-0.024, -0.008)	<0.001	-0.074 (-0.127, -0.021)	0.01	-0.090 (-0.142, -0.036)	<0.001	0.168 (0.078, 0.451)	<0.001
Heat shock 70 kDa protein 1A	-0.018 (-0.026,	<0.001	-0.083 (-0.136,	0.004	-0.101 (-0.154,	<0.001	0.175 (0.087, 0.386)	<0.001

	-0.010)		-0.029)		-0.047)			
Heat shock 70 kDa protein 1A	-0.016 (-0.025, -0.008)	<0.001	-0.069 (-0.123, -0.014)	0.016	-0.085 (-0.139, -0.032)	0.002	0.189 (0.083, 0.535)	0.002
Secretoglobin family 3A member 1	0.017 (0.012, 0.023)	<0.001	0.067 (0.039, 0.094)	<0.001	0.084 (0.057, 0.111)	<0.001	0.204 (0.134, 0.319)	<0.001
Heat shock 70 kDa protein 1A	-0.019 (-0.027, -0.011)	<0.001	-0.069 (-0.124, -0.013)	0.016	-0.088 (-0.143, -0.032)	0.002	0.209 (0.105, 0.602)	0.002
Cyclic AMP-dependent transcription factor ATF-6 alpha	-0.027 (-0.036, -0.020)	<0.001	-0.083 (-0.117, -0.046)	<0.001	-0.110 (-0.146, -0.073)	<0.001	0.244 (0.163, 0.388)	<0.001
Tetranectin	-0.013 (-0.020, -0.007)	<0.001	-0.039 (-0.087, 0.005)	0.106	-0.052 (-0.100, -0.009)	0.024	0.247 (0.086, 1.236)	0.024
Cholinesterase	-0.022 (-0.030, -0.015)	<0.001	-0.040 (-0.079, -0.001)	0.044	-0.061 (-0.100, -0.024)	<0.001	0.353 (0.195, 0.968)	<0.001

Beta-enolase	-0.036 (-0.047, -0.025)	< 0.001	-0.029 (-0.076, 0.025)	0.25	-0.064 (-0.111, -0.011)	0.018	0.535 (0.284, 2.396)	0.018
EGF-containing fibulin-like extracellular matrix protein 1	-0.007 (-0.013, -0.003)	0.004	-0.141 (-0.179, -0.106)	< 0.001	-0.148 (-0.186, -0.113)	< 0.001	0.050 (0.017, 0.091)	0.004
Lymphatic vessel endothelial hyaluronic acid receptor 1	-0.007 (-0.012, -0.001)	0.004	-0.110 (-0.149, -0.071)	< 0.001	-0.117 (-0.155, -0.076)	< 0.001	0.056 (0.013, 0.123)	0.004
Protocadherin gamma-A1	0.004 (0.001, 0.008)	0.006	-0.048 (-0.072, -0.023)	< 0.001	-0.043 (-0.068, -0.019)	< 0.001	-0.097 (-0.255, -0.024)	0.006
Heat shock 70 kDa protein 1A	-0.008 (-0.016, -0.001)	0.026	-0.092 (-0.150, -0.038)	0.002	-0.100 (-0.157, -0.047)	0.002	0.082 (0.008, 0.228)	0.028
Interferon lambda receptor 1	-0.006 (-0.013, -0.000)	0.036	-0.067 (-0.118, -0.019)	0.002	-0.073 (-0.122, -0.026)	< 0.001	0.084 (0.005, 0.288)	0.036

IQ domain-containing protein F1	-0.008 (-0.016, -0.000)	0.05	0.134 (0.075, 0.198)	<0.001	0.126 (0.068, 0.189)	<0.001	-0.062 (-0.162, -0.000)	0.05
Heat shock 70 kDa protein 1A	-0.007 (-0.014, 0.000)	0.054	-0.085 (-0.144, -0.027)	0.002	-0.092 (-0.152, -0.032)	<0.001	0.076 (-0.001, 0.258)	0.054
Pancreatic triacylglycerol lipase	0.005 (-0.001, 0.012)	0.102	0.451 (0.403, 0.497)	<0.001	0.456 (0.410, 0.502)	<0.001	0.011 (-0.002, 0.026)	0.102
Angiopoietin-2	-0.005 (-0.012, 0.001)	0.122	-0.150 (-0.193, -0.102)	<0.001	-0.155 (-0.198, -0.107)	<0.001	0.031 (-0.008, 0.077)	0.122
Asporin	-0.005 (-0.012, 0.001)	0.124	-0.088 (-0.141, -0.035)	0.002	-0.093 (-0.146, -0.042)	0.002	0.054 (-0.014, 0.184)	0.126
Zymogen granule membrane protein 16	-0.005 (-0.012, 0.002)	0.134	-0.081 (-0.136, -0.028)	0.01	-0.086 (-0.141, -0.033)	0.004	0.060 (-0.028, 0.210)	0.138
Mucin-16	-0.002 (-	0.432	-0.082 (-	<0.0	-0.084 (-	<0.0	0.028 (-0.041,	0.432

	0.008, 0.003)		0.128, -0.037)	01	0.128, -0.039)	01	0.130)	
ADP-ribosylation factor 1	0.003 (-0.004, 0.009)	0.44	0.068 (0.014, 0.121)	0.008	0.071 (0.018, 0.123)	0.006	0.038 (-0.066, 0.256)	0.434

Supplement Table 12: *Results from multivariable logistic regression analyses between tetranectin, fibulin-1, MACE and history of CAD subset by T2DM and non-T2DM in the PROMISE cohort.* Multivariable logistic regression analyses demonstrated significantly lower levels of circulating tetranectin in cohorts with T2DM as well as non-T2DM. T2DM only cohort results and Fibulin-1 results did not meet significance. Covariates included age, sex, race, BMI, HTN, smoking status, statin, history of cardiovascular disease, history of heart failure, history of hyperlipidemia, glomerular filtration rate. *Significant p-value <0.05

Promise Cohort	Analysis	OR (95% CI)	p-value
Full Cohort (N=860)	CAD ~ tetranectin + covariates	0.80 (0.68, 0.93)	0.0041*
Full Cohort (N=860)	CAD ~ fibulin-1 + covariates	1.13 (0.88, 1.19)	0.38
Full Cohort (N=860)	MACE ~ tetranectin + covariates	0.88 (0.80, 0.98)	0.013 *
Full Cohort (N=860)	MACE ~ fibulin-1 + covariates	0.98 (0.90, 1.07)	0.33
T2DM Only (N=178)	CAD ~ tetranectin + covariates	0.78 (0.52, 1.15)	0.20
T2DM Only (N=178)	CAD ~ fibulin-1 + covariates	1.10 (0.75,1.24)	0.36
T2DM Only (N=178)	MACE ~ tetranectin + covariates	0.99 (0.79, 1.24)	0.94
T2DM Only (N=178)	MACE ~ fibulin-1 + covariates	0.99 (0.85, 1.14)	0.88
Non-T2DM Only (N=681)	CAD ~ tetranectin + covariates	0.79 (0.67, 94)	0.0082 *
Non-T2DM Only (N=681)	CAD ~ fibulin-1 + covariates	1.12 (0.78 ,1.23)	0.32

Non-T2DM Only (N=681)	MACE ~ tetranectin + covariates	0.85 (0.76,0.94)	0.002 *
Non-T2DM Only (N=681)	MACE ~ fibulin-1 + covariates	0.99 (0.88, 1.3)	0.21

Supplement Figure 5: *Boxplot of tetranectin levels stratified by T2DM and MACE in the PROMISE cohort.* ANOVA testing of mean circulating tetranectin levels in cohorts +/- T2DM and +/- MACE in Promise cohort demonstrated significant lower levels of tetranectin in T2DM+ compared to T2DM- and significant lower levels of tetranectin in MACE+ compared to MACE-, p = 0.049. *Significant p-value <0.05

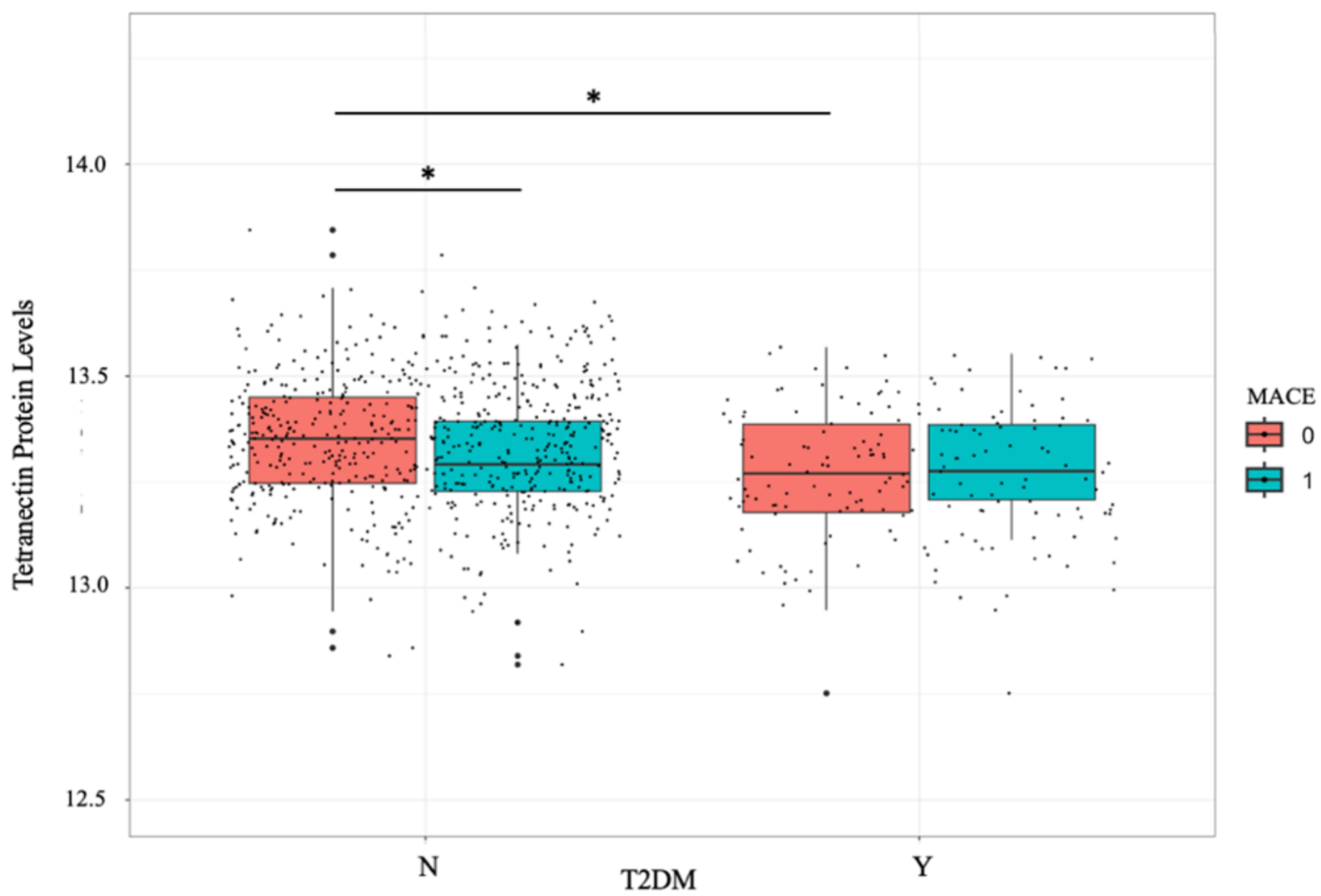

Supplement Table 13: *Post-hoc Tukey honestly significant difference results of tetranectin levels (relative quantification) in MACE and T2DM subsets of the PROMISE cohort.* Post-hoc testing demonstrated significantly lower levels of tetranectin in people with T2DM and MACE compared to non-T2DM. *Significant p-value <0.05

Group 1	Group 2	Estimate	95% CI Lower Bound	95% CI Upper Bound	FDR adjusted p-value
T2DM+/MACE+	T2DM-/MACE+	-0.035	-0.19	0.11	0.94
T2DM+/MACE+	T2DM+/MACE-	-0.035	-0.18	0.11	0.92

T2DM+/MACE+	T2DM-/MACE-	0.046	-0.092	0.18	0.83
T2DM–/MACE+	T2DM+/MACE-	0.00063	-0.076	0.078	1
T2DM–/MACE+	T2DM-/MACE-	0.081	0.0088	0.15	0.021 *
T2DM+/MACE-	T2DM-/MACE-	0.081	0.047	0.12	$4.9e^{-9}$ *
